# Effect of Temperature and Genetic Inheritance on the Number of Mycangium Pits in Female *Platypus quercivorus* (Coleoptera: Curculionidae: Platypodinae)

**DOI:** 10.3390/insects17060536

**Published:** 2026-05-22

**Authors:** Gabriel Fiakpornu, Naoto Kamata

**Affiliations:** 1 International Program in Agricultural Development Studies, Graduate School of Agricultural and Life Sciences, The University of Tokyo, Bunkyo, Tokyo 113-8657, Japan; 2 The University of Tokyo Chiba Forest, Graduate School of Agricultural and Life Sciences, The University of Tokyo, Kamogawa 299-5503, Japan; kamatan@uf.a.u-tokyo.ac.jp

**Keywords:** ambrosia beetle, Japanese oak wilt, maternal effects, phenotypic plasticity, *Dryadomyces quercivorus*, upper developmental threshold

## Abstract

Ambrosia beetle *Platypus quercivorus* is a vector of Japanese oak wilt. Only females possess 4−12 mycangial pits on the pronotum, which are essential for carrying symbiotic fungi and are thought to be a significant determinant of beetle fitness. However, the factors influencing the pit numbers remain poorly understood. To elucidate the effects of environmental conditions and parental traits on the pit number, we conducted a controlled breeding experiment. Collected *P. quercivorus* broods were categorized into three groups (large, middle and small) based on their average number of maternal mycangial pits. Excluding the middle groups, male−female pairs from the same group (large or small) were inoculated into *Quercus crispula* logs and incubated under four temperature regimes: 18, 22, 26, and 30 °C. We analyzed offspring pit number and body weight relative to temperature and parental traits. Both traits increased at lower temperatures likely because accelerated metabolism and developmental rates at higher temperatures, reduces the time and resources available for body growth and pit development. Notably, the traits exhibited distinct inheritance patterns: offspring pit number was significantly influenced by maternal pit number but not by the body weight of either parent. In contrast, offspring body weight was significantly correlated with maternal pit number, paternal body weight, and maternal body weight. Thus, we conclude that temperature plays a critical role in shaping these traits. Parental effects indicate that both mycangium pit number and body weight are heritable in *P. quercivorus*. However, their distinct inheritance patterns suggest a weak genetic association between the traits, implying that they may evolve largely independently.

## Simple Summary

Japan’s oak forests are under attack by a specific ambrosia beetle that spreads a fungus causing Japanese oak wilt. Female beetles of this species carry the fungus in special structures on the surface of their bodies. However, it is not known why some females have more of these structures than others. As these structures enable the fungus to be transported, identifying the factors that influence their number could help us better understand how the disease spreads. In this study, we examined whether temperature and parental traits influence the number of pits in the next generation of beetles. We raised beetles in controlled conditions at different temperatures and compared the offspring of parents that had either many or few pits. We found that beetles raised at cooler temperatures developed larger bodies and more pits, while those raised at warmer temperatures were smaller and had fewer pits. In addition, daughters of mothers with more pits tended to develop more pits themselves, indicating a genetic influence. These results show that both environmental conditions and inheritance shape this important trait. Understanding how climate and genetics affect beetle development will help scientists better predict the spread of oak wilt and support efforts to protect forests.

## 1. Introduction

Forests contain the largest biomass of any terrestrial ecosystem [1], but much of this biomass is made up of polymeric compounds called cellulose, lignin, and hemicellulose that are extremely difficult to digest [2]. To exploit these substrates, many insects rely on intimate symbioses with microorganisms, including bacteria, fungi, yeasts, and protozoa. An example is the mutualism between lower termites (Infraorder Isoptera) and hindgut flagellate protozoa (Order Hypermastigida), which degrade cellulose and hemicellulose into utilizable compounds and thereby enable wood feeding [3,4,5], while the host provides a stable anaerobic habitat and nutrients for the symbionts [6]. Fungi likewise form diverse mutualistic associations with insects, contributing to nutrition in numerous insect lineages [7]. In some wood associated insects, these fungal partners are environmentally acquired and assist in cellulose decomposition without being housed in specialized transport structures [8]. For example, passalid beetles (Coleoptera: Passalidae) harbor fungi within their galleries, where fungal growth partially degrades structural polymers and renders the wood more accessible to the beetles [9]. However, in some groups of insects, the fungal partners are transported in specialized structures termed mycangia, which are cuticular invaginations such as pits, tubes, or pouches typically associated with secretory gland cells [10,11]. These glands produce secretions that maintain fungal viability and purity during dispersal [12,13]. Mycangia occur across diverse xylomycetophagous lineages, including wood wasps (Hymenoptera: Siricidae) [11,14] and certain beetles (Coleoptera) such as stag beetles (Lucanidae) [15] and ship timber beetles (Lymexylidae) [16] in which fungi are associated with conditioning woods as their food. However, the mycangia are most specialized in ambrosia beetles, where the fungi serve as nutritional resource [17]. The structure and position of the mycangia vary widely, occurring in the head, thorax including pronotal or mesonotal regions, abdomen, or coxa and ranging from simple cuticular pits to complex gland lined sacs, reflecting repeated evolutionary origins and functional specialization [10,18].

Historically, ambrosia beetles were regarded as secondary colonizers of stressed or newly dead trees [19,20] and play an important role in early stage of wood decomposition [21]. However, certain beetle–fungus symbioses are now recognized for their ability to induce systemic disease and cause large-scale forest declines worldwide. Notable examples include laurel wilt in the United States [22], Fusarium dieback in California and Israel [23]. In East Asia, similar threats are posed by *Platypus koryoensis* (Murayama, 1930), which has caused mortality of *Quercus mongolica* Fisch. ex Ledeb. in Korea [24]. Most significantly in Japan, Japanese oak wilt (JOW) represents a similar threat [25]; it is caused by the fungus *Dryadomyces quercivorus* (Kubono & Shin. Ito) M. Procter & Z.W. de Beer, 2022 (formerly *Raffaelea quercivora*) [26] which is transmitted to trees belonging to the family Fagaceae (with the exception of the genus Fagus L.) by the native Platypodine ambrosia beetle *Platypus quercivorus* (Murayama, 1925) functioning as a vector [25,27].

In Japan, seasonal temperature fluctuations strongly influence the phenology of *P. quercivorus* [28]. The beetle primarily overwinters as late-instar larvae within host xylem galleries and resumes development in spring as temperatures rise [29]. Adult emergence and flight activity typically occur from late May through July [28], broadly overlapping with spring leaf expansion and renewed growth activity of deciduous *Quercus* hosts.

Among the Platypodinae, pronotal pit mycangia are characteristic and show marked interspecific variation in number and arrangement [30]. In *P. quercivorus*, these mycangia are sexually dimorphic, occurring only in females as five to ten pits located centrally on the pronotum [31]. During gallery excavation, females inoculate the wood with their fungal symbionts. Subsequent growth of *D. quercivorus* hyphae into the sapwood intercellular spaces induces cellular necrosis and disrupts vessel function [32]. While *P. quercivorus* can reproduce in both living and dead hosts [33], it crucially differs from secondary ambrosia beetles by being able to target healthy trees. When numerous infections occur during mass attacks, impairment of the conductive system results in tree-level wilting [32].

Because mycangia serve as the primary mechanism for symbiont delivery, it is expected that variation in pit numbers may directly influence the efficiency of fungal transmission and disease incidence. However, it remains unclear whether the observed variation in the pit number is environmentally induced or genetically determined. Understanding how environmental factors—specifically temperature—interact with genetic inheritance to determine the mycangial pit number in *P. quercivorus* is essential for explaining the JOW incidence.

In this study, we examined the effects of temperature and genetic inheritance on mycangial pit number in female *P. quercivorus*. Using parental lines established by pit number, we reared offspring beetles under controlled temperatures to evaluate the relative contributions of maternal and paternal traits, alongside temperature, to the phenotypic variation in the number of the pits and body weight. We tested three primary hypotheses: (1) lower developmental temperatures result in an increased number of mycangial pits and larger body size; (2) phenotypic variation in mycangial pits is significantly influenced by parental (specifically maternal) pit numbers, indicating heritability of the trait; and (3) temperature exerts a stronger influence on pit number variation than genetic inheritance.

## 2. Materials and Methods

### 2.1. Study Sites and Insect Collection

The experiment was conducted at the University of Tokyo Tanashi Forest (UTTF; 35.7297° N, 139.5383° E) between May 2023 and August 2023 and the University of Tokyo Chichibu Forest (UTCF; 35.9833° N, 139.0763° E) between August 2023 and May 2024.

**Figure 1:**
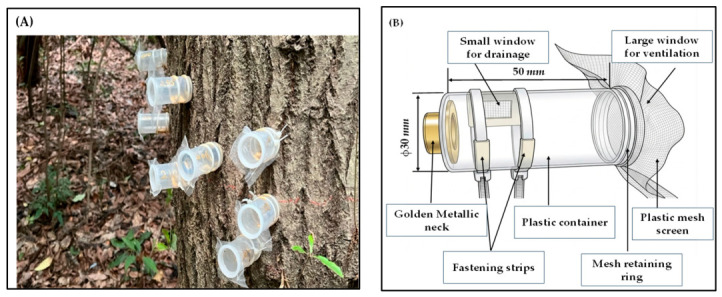
Emergence trap used to collect newly emerged adults of *Platypus quercivorus* (Murayama, 1925) from galleries in infested trees. (**A**) Traps were installed on tree trunks, each positioned to enclose a single active gallery characterized by fresh powdery frass. (**B**) The trap has two distinct windows: a smaller window for drainage and a larger window for ventilation. A metallic component anchors the trap body to the tree trunk.

Adult *P. quercivorus* were collected from JOW-killed *Quercus serrata* trees at UTTF. To capture adults emerging from single galleries, emergence traps were installed on 24 May 2023, over entrance holes created by individual pairs of adults (Figure 1A). Each trap consisted of a compact plastic sample container (ϕ30 × 50 mm in depth), plastic mesh, a circular ring, and a metallic neck component hammered into the trunk (Figure 1B). The design featured two windows: a small lower opening for drainage and a larger upper opening for ventilation.

**Figure 2:**
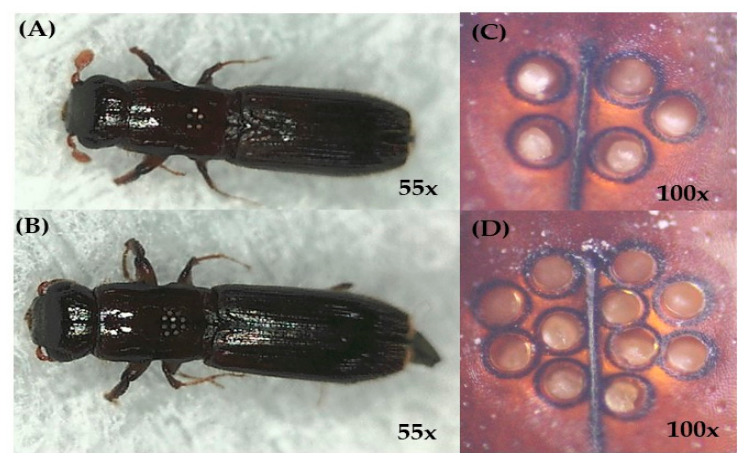
Body size of *Platypus quercivorus* (Murayama) females (**A**,**B**) and their mycangia (**C**,**D**). Adult females were photographed at 55× magnification and mycangia at 100× magnification. Panels (**A**,**C**) show a female with five mycangial pits, whereas panels (**B**,**D**) show a female with eleven pits, illustrating variation in body size and pit number (photos by Andreas Ade Kristian).

Traps were inspected on weekdays (Monday to Friday, excluding Japanese national holidays) from 31 May to 2 August 2023. A reference set of adults collected by 30 June was preserved separately in microtubes with 99% ethanol, organized by brood and collection date (Table S1). From these samples, sexing was performed based on the presence or absence of mycangial pits on the pronotum using a magnifier. The pits on each female were then counted to calculate the mean pit number for each brood (Figure 2).

### 2.2. Classification of Size Groups

Using the mean pit number per brood (Table S2), we determined the boundary between small and middle-sized groups and that between middle and large-sized groups as small group (≤6.57), medium group (>6.57 and <7.90), and large group (≥7.90). While including later-captured data would have increased the reliability of the three-group classification, we had to find a compromise between classification accuracy and ensuring a sufficient number of surviving individuals for subsequent experiments. Analysis conducted through 15 July 2023, revealed that females captured by 30 June provided the effective criteria for classifying individuals into these three size groups posteriori (Figure S1).

To evaluate the genetic effects on the number of mycangium pits, we paired a male and a female from different broods within the same size category (small or large). These pairs were inoculated into logs starting 3 July 2023. The medium-sized group was subsequently excluded, as we intended to enhance the detection of genetic effects by focusing on the extreme size categories.

### 2.3. Log Preparation and Inoculation

Logs of *Q. crispula* Fisch. ex Ledeb (free of JOW) were prepared at the University of Tokyo Hokkaido Forest and transported to UTTF (*n* = 16; length ≈ 140 cm, diameter range: 13.1–28.2 cm). To prevent desiccation, logs were wrapped in plastic film, and the cut ends were sealed with wax. Six inoculation points were marked longitudinally on each log at 20 cm intervals, maintaining a 20 cm margin from the ends. Following the mounting of emergence traps at each point, logs were positioned horizontally on the ground for inoculation.

As *P. quercivorus* is monogynous and males only accept females after initiating gallery construction [34,35], males were introduced to the logs first via the traps. Once entrance holes were confirmed by the presence of stringy wood fibers, a female was introduced. Each log received six treatments, alternating between three small-group (S1–S3) and three large-group (L1–L3) inoculations. Gallery extension and successful reproduction were distinguished by a transition from limited male-produced fibrous frass to fibrous aggregates (rolled into balls) by the female, and finally to powdery larval frass [36,37,38].

### 2.4. Incubation and Data Collection

Following mating, the inoculated logs were transferred on 20 August 2023, to the Kagemori Nursery of UTCF for incubation. Four incubation chambers were maintained at 18, 22, 26, 30 °C under a 14L-10D photoperiod, and 75% relative humidity. During incubation, frass was appropriately cleared from the traps not to prevent gallery construction and adult emergence. Newly emerged adults were collected daily, and sexing was performed. Females were preserved individually in microtubes containing 99.9% ethanol, each labeled with the collection date and the number of mycangial pits. Conversely, males from the same brood and collection date were pooled in a single microtube of 99.9% ethanol. These microtubes were then organized by brood and date into small plastic bags, which were subsequently grouped by brood ID into larger plastic bags.

To measure body weight, both the parental reference group and the newly emerged adults were removed from ethanol, air-dried on filter paper for 6 h, and then stored overnight in a desiccator with silica gel. Each adult was weighed individually using an ultra microbalance (UMT2, METTLER TOLEDO, Columbus, OH, USA) to record its dry body weight to the nearest 0.0001 mg (=0.1 μg).

### 2.5. Statistical Analysis

All statistical analyses were conducted using R version 4.5.1 [39]. The number of emerged females per gallery was analyzed using a generalized linear model (GLM), with a Poisson distribution and a log-link function, following the form:

(1) $$Log\left(µ\right)=\beta_{0}+\beta_{1}X_{1}+\ldots +\beta_{n}X_{n}+log(offset)$$

where *µ* represents the expected count. A log-transformed offset of the number of successful galleries was included to account for differences in gallery counts. Explanatory variables included temperature, the parental group (large or small), and their interaction. Offspring body weight was analyzed using a Gaussian GLM with the same explanatory variables, allowing assessment of both temperature and parental groups on body size. Additionally, simple linear regression was used to quantify the relationship between offspring number of mycangium pits (*Y*) and the body weight (*X*), expressed as;

(2) $$Ƴ=\beta_{0}+\beta_{1}X+ɛ$$

Poisson generalized linear models were applied to count data assuming independent observations, no substantial overdispersion, and an appropriate residual structure. Overdispersion was evaluated using the ratio of residual deviance to residual degrees of freedom, and residual patterns were examined graphically. Gaussian generalized linear models and linear regression analyses assumed linearity, normality of residuals, homogeneity of variance, and independence of observations. Normality and homogeneity of variance were evaluated using Q–Q plots and residual-versus-fitted plots. Multicollinearity among explanatory variables was assessed using variance inflation factors (VIFs). Because multiple offspring originated from the same brood, potential non-independence among observations was assessed by comparing brood-level variation with overall population variation. Model diagnostics showed no major violations of assumptions, including substantial overdispersion, heteroscedasticity, multicollinearity, or strong within-brood dependence. Comparative distribution analyses were presented using box plots across three temperature treatments (18 °C, 22 °C, and 26 °C) and two maternal pit-number groups (large or small), incorporating the median, mean, and sample sizes for successful galleries and individuals. Additionally, histograms were used to illustrate the frequency distribution of mycangium pit numbers. To evaluate factors affecting the number of mycangium pits in female offspring, seven Poisson GLMs using the same offset as mentioned above were developed representing different combinations of explanatory variables, including temperature, number of maternal pits, maternal weight, and paternal weight, with composite models combining these variables. Model selection was conducted based on the Akaike Information Criterion (AIC), with the model having the lowest AIC considered the most parsimonious for explaining offspring pit numbers.

## 3. Results

### 3.1. Reproductive Output

**Figure 3:**
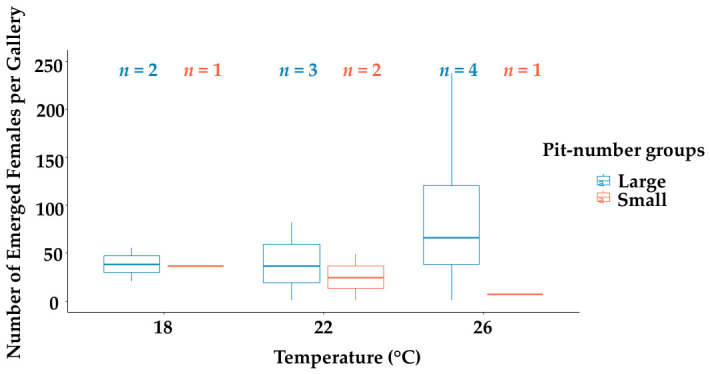
Female reproductive success of *Platypus quercivorus* across temperature and pit-number groups. The number of emerged female offspring per successful gallery is compared across three temperature treatments (18 °C, 22 °C, and 26 °C) and two maternal pit-number categories (Large and Small). Data are presented as box plots where the horizontal line represents the median, the box indicates the interquartile range (25th–75th percentiles), and the whiskers extend to the minimum and maximum values. The “*n*” values above the boxes indicate the number of successful galleries per treatment group. No offspring were obtained at 30 °C.

New adults of *P. quercivorus* emerged from all temperature treatments except 30 °C, although pairing and inoculation were successful. Therefore, only individuals from the three successful temperature treatments (18, 22, and 26 °C) were included in subsequent analyses (Table S3). Female reproductive output varied across temperatures and between pit-number groups (Figure 3). Temperature had a strong positive effect on female reproductive output in pairs from the large pit-number group using a Poisson GLM with the number of successful galleries as an offset, with higher temperatures resulting in more females per gallery (GLM, coefficient = 0.486, *p* < 0.001) (Table 1). However, pairs from small pit-number group produced significantly fewer females per gallery than those from the large pit-number group (GLM, coefficient = −0.892, *p* < 0.001). Furthermore, a significant interaction between temperature and pit-number groups on reproductive output was detected, with a negative interaction coefficient (GLM, coefficient = −1.078, *p* < 0.001). This indicates that the temperature slope was smaller for pairs in the small pit-number group than for those in the large pit-number group. Comparing this with the main effect of temperature (0.486) shows that reproductive output decreased with increasing temperature in pairs from the small pit-number group, because the estimated slope for the small pit-number group was negative (0.486 − 1.078 = −0.592). The number of successful galleries exhibited a pattern similar to reproductive output per gallery; more successful galleries were found in the large pit-number group than the small ones (Figure 3). The number of successful galleries increased with temperature in the large pit-number group but showed no clear temperature-related trend in the small pit-number group.

### 3.2. Offspring Body Weight

**Figure 4:**
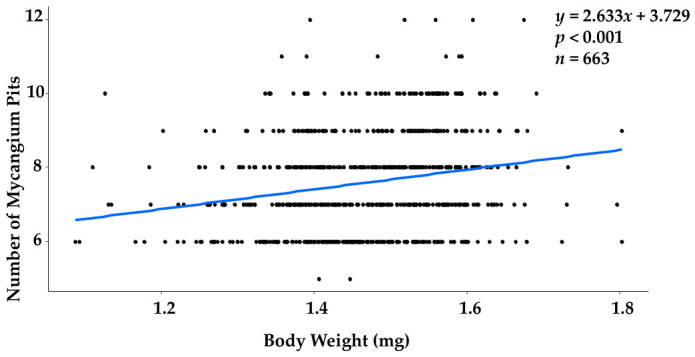
Relationship between offspring body weight (mg) and the number of mycangium pits in female *Platypus quercivorus.* Blackpoints represent individuals; the blue line shows the linear regression. Regression equation, *p*-value, and sample size (*n*) are shown.

A significantly positive relationship was found between offspring body weight and the number of mycangium pits (r = 0.211, *p* < 0.001 (Figure 4)).

**Figure 5:**
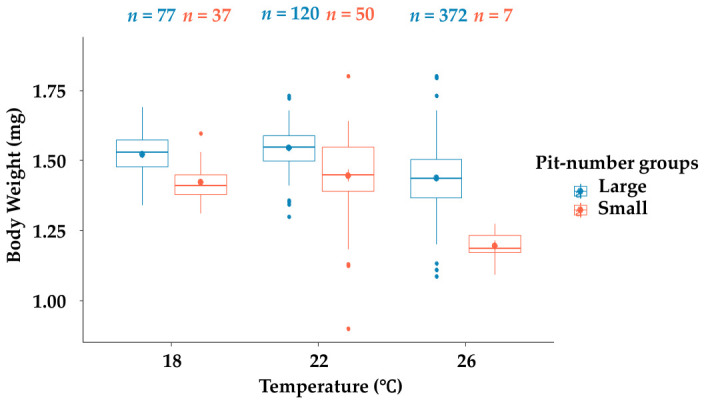
Body weight of female *Platypus quercivorus* offspring across temperature and maternal pit-number categories. The distribution of female offspring body weight (mg) is compared across three temperature regimes (18 °C, 22 °C, and 26 °C) for both “Large” and “Small” maternal pit-number groups. Data are presented as box plots where the horizontal line represents the median, and the box indicates the interquartile range (25th–75th percentiles). The whiskers extend to the minimum and maximum values. Red and blue dots indicate the mean values for the large and small groups, respectively. Sample sizes (*n*) representing the total number of measured offspring are provided above each box. No offspring were obtained at 30 °C. For overall statistical significance and model-derived trends, please refer to Table 2 (where temperature is evaluated as a continuous variable).

Offspring body weight differed among temperatures in both large and small groups (Figure 5). Although mean body weight was similar between 22 °C and 18 °C in both large and small groups, temperature had a significant negative effect on offspring body weight (GLM, coefficient = −0.043, *p* < 0.001) (Table 2), indicating that body weight decreased with increasing temperature. Offspring from pairs in the small group were also significantly lighter than those from the large group (GLM, coefficient = −0.087, *p* < 0.001) (Table 2). The interaction between temperature and groups was not significant (GLM, coefficient = 0.009, *p* = 0.543) (Table 2), indicating that the slope of the relationship between temperature and body weight did not differ significantly between the two groups.

### 3.3. Effect of Temperature and Parental Traits on Mycangial Pits

**Figure 6:**
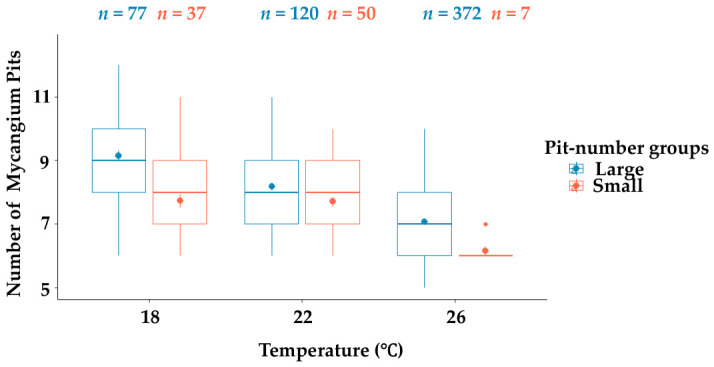
Distribution of mycangium pits numbers in female *Platypus quercivorus* offspring under varying temperature regimes. Comparison of offspring pit numbers across three temperature treatments (18 °C, 22 °C, and 26 °C) for both “Large” and “Small” maternal pit-number categories. Data are illustrated using box plots, where the central horizontal line denotes the median and the box boundaries represent the interquartile range (25th to 75th percentiles). Whiskers extend to the minimum and maximum observed values. Red and blue dots within the boxes indicate the mean values for the large and small groups, respectively. The total number of offspring evaluated (*n*) is specified above each corresponding box. The results at No offsprings were obtained at 30 °C. For overall statistical significance and model-derived trends, please refer to Table 3 (where temperature is evaluated as a continuous factor).

Number of offspring mycangial pits differed among temperatures in both large and small groups (Figure 6). Although mean number of offspring mycangial pits was similar between 22 °C and 18 °C in both large and small groups, a Poisson generalized linear models evaluating the effects of temperature and parental traits on number of offspring mycangial pits consistently identified temperature as a strong negative explanatory variable when included in the models (Table 3). The temperature model (Model 1) detected a strong and significant decrease in number of offspring mycangial pits with increasing temperature (GLM, coefficient = −0.083, *p* < 0.001) and had the best fit among all single-predictor models (AIC = 2696.9). Models that included parental traits alone explained less variation. Number of maternal pits (Model 2) had no significant effect (GLM, *p* = 0.187), maternal weight (Model 3) showed only a weak, significant effect (GLM, *p* = 0.052), and paternal weight (Model 4) showed a positive and significant effect (GLM, *p* = 0.003). However, despite its statistical significance, the paternal weight model fit the data much worse than the temperature model based on AIC. Among the parental-trait models, paternal weight provided the best fit, but its contribution was small compared with that of temperature. Models that included temperature consistently performed better than models without temperature. When temperature was combined with parental traits, only the model that included number of maternal pits along with temperature (Model 5) improved on the temperature model (Model 1) and showed a strong negative effect of temperature and a significant positive effect of number of maternal pits on the number of offspring mycangial pits, whereas adding maternal or paternal weight along with temperature did not improve model fit and did not produce significant effects.

### 3.4. Distribution of Mycangial Pits Across Temperatures

**Figure 7:**
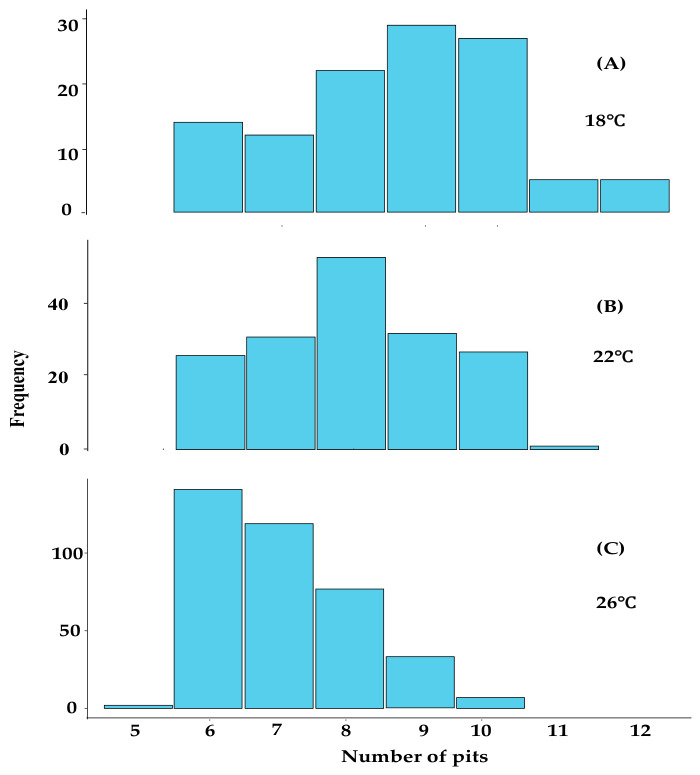
Frequency distribution of the number of mycangium pits in female *Platypus quercivorus* individuals under different temperature treatments: (**A**) 18 °C; (**B**) 22 °C; and (**C**) 26 °C. The panels illustrate the effect of temperature on the variation of the number of pits. No offsprings were obtained at 30 °C.

Distribution of number of mycangial pits in female offspring differed markedly among temperature treatments (Figure 7). The modal number of pits declined from 9 at 18 °C to 6 at 26 °C. In addition, the maximum number of pits decreased from 12 to 10 with increasing temperature. Similarly, the minimum number of pits decreased from 6 to 5.

## 4. Discussion

*Platypus quercivorus* adults become more active at relatively high temperatures [40]: adults start to fly when air temperature reach 19 °C or higher [41], indicating increased behavioral activity. Boring activity of *P. quercivorus* which leads to active tunnel expansion, peaks during the warmer months of June and July [35]. While partial bivoltinism can occur in Japan [29,42], our study focuses on the primary annual generation. Our experimental data support the findings of Sonè et al. [35]; at 18 °C, frass production by females [37], was minimal, indicating limited extension of the maternal galleries. These behavioral constraints likely contributed to reduced egg deposition and lower reproductive output at 18 °C. In contrast, heightened behavioral activity at warmer temperatures, such as 26 °C, enables females to extend galleries more efficiently. This provides a more suitable environment for the development of larger broods, explaining the significantly higher reproductive output observed in our results (Figure 3, Table 1). The increase in gallery success at higher temperatures likely stems from accelerated metabolic rates, which facilitate more rapid gallery excavation and faster colonization by the symbiotic fungus *D. quercivorus*. This increased speed may allow the beetles to overwhelm host physiological defenses more effectively during the critical initial attack phase, leading to a higher proportion of successful establishments despite the smaller individual body size observed in the resulting offspring.

However, larval growth responded to temperature in the opposite manner (Figure 5, Figure 6 and Figure 7). Offspring tended to be smaller in weight and possessed a smaller number of mycangial pits as temperature increased (Table 2 and Table 3, Figure 6 and Figure 7). This pattern may be attributed to differences in developmental rates; while warmer temperatures accelerate development, cooler temperatures prolong the larval period [43]. In ambrosia beetles, body size increases with greater consumption of fungal food [44]. It is possible that lower temperatures facilitate greater biomass accumulation and a higher number of mycangial pits by allowing for an extended feeding duration and a reduced metabolic rate. However, it remains to be determined whether larvae at lower temperatures consume a greater absolute quantity of food or if their total metabolic costs (metabolic rate × time) are significantly lower.

These contrasting responses indicate that the optimal temperature for *P. quercivorus* differs depending on the insect developmental stage. While warmer temperatures (26 °C) appear to favor adult behavioral performance and maximize the number of offspring, cooler temperatures (18 °C) favor larval growth and offspring quality.

In the present study, no offspring emerged at 30 °C, although successful development was observed at 18, 22, and 26 °C. Previous studies have also successfully reared *P. quercivorus* at temperatures below 30 °C (e.g., [36]). Notably, *P. quercivorus* adults were highly active at 30 °C: males readily initiated entrance hole construction, pairing and copulation occurred successfully, and continuous production of powdery frass by larvae indicated that eggs had hatched and larvae had grown to some extent. The absence of offspring, therefore, reflects mortality during post-hatching development. One plausible explanation is that the upper thermal limit for successful larval development of *P. quericovorus* lies below 30 °C. Another possibility is the failure of the larval food source; exposure to 30 °C may have exceeded the physiological tolerance of the fungal symbiont, resulting in the collapse of the fungal resources required for larval growth. *Platypus quercivorus* is commonly associated with *D. quercivorus*, a filamentous fungus that mainly occupies the general gallery system, and with yeasts that dominate the larval cradles [45]. Among these yeasts, *Candida kashinagacola* is a frequently isolated species associate of *P. quercivorus* [45,46], and has been suggested to be a primary dietary fungus for the beetle [45]. Although limited information is available on the upper thermal tolerance of this yeast isolated from *P. quercivorus* and its galleries, *C. kashinagacola* has been successfully incubated at a standard growth temperature of approximately 25 °C [47]. *Candida kashinagacola* isolated from the congeneric beetle *P. koryoensis* was successfully cultured at 28 °C but failed at 37 °C [48]. Because exceeding a yeast’s maximum growth temperature is known to cause loss of membrane integrity and cessation of budding [49], it is possible that *C. kashinagacola* could not grow sufficiently at 30 °C, resulting in the reproductive failure of *P. quercivorus*.

Given these temperature-dependent differences in adult activity and larval growth, the variation in the number of mycangial pits observed among offspring is likely an outcome of larval developmental conditions. Because the number of pits is expressed only after the successful completion of larval development, the number of pits reflects the cumulative effects of temperature on the larval development. Along with these environmental conditions (temperature), the number of mycangial pits in *P. quercivorus* was also influenced by genetic factors; specifically, number of maternal pits, maternal weight, and paternal weight each had a positive influence on offspring. However, temperature exerted a strong and consistent negative effect across all models (Table 3). This effect remained robust even after accounting for parental traits, identifying temperature as the primary driver of the number of pits. Ultimately, the best-fit model incorporated both temperature and maternal pits, revealing that the effect of temperature was 2.6 times stronger than that of maternal inheritance, confirming it as the strongest predictor of the number of mycangial pits in *P. quercivorus*.

Although a significant positive relationship was observed between the number of mycangial pits and body weight, the correlation was weak (Figure 4). Morphological traits related to body size frequently exhibit positive correlations, as various structures often scale with overall body size despite differences in function or developmental pathways [50,51]. However, these allometric relationships are typically weak because distinct traits are rarely governed by identical sets of genes or developmental processes [52,53]. In insects, the size of a specific structure is shaped by both localized developmental regulation and systemic growth, leading to traits that co-vary without being strictly coupled [51,54]. In this context, the weak positive association between female body weight and mycangial pit number in *P. quercivorus* suggests that while pit number is influenced by overall body size, it is also governed by independent genetic and developmental factors.

Regarding the influence of the number maternal pits on the number of offspring mycangial pits, two primary pathways—maternal effects and genetic inheritance—must be considered. Maternal effects are commonly manifested through influences on offspring growth, physiological condition, and resource availability [55,56]. Given that mycangia serve to transport and maintain fungal symbionts [11,17,57], females with more mycangial pits may provide a developmental advantage to their larvae by carrying a larger or more stable fungal inoculum. Indeed, our results showed that both offspring body weight (Table 2; Figure 5) and offspring pit number (Table 3; Figure 6) varied significantly across maternal pit-number groups. This suggests that mothers with higher pit counts may inoculate more fungal symbionts, thereby providing the nutritional resources necessary for larvae to reach the developmental potential required for a higher number of pits.

While the first pathway operates via maternal environmental effects (i.e., resource provisioning), the second pathway involves genetic inheritance. After accounting for temperature, maternal pit number became a significant positive predictor of offspring pit number (Model 5 in Table 3). This aligns with the principle that genetic variation dictates trait expression within environmentally defined limits [51]. Notably, while offspring pit number correlated with their own body weight (Figure 4), maternal body weight itself did not explain number of offspring mycangial pits once temperature was controlled. In contrast, number of maternal pits became a significant predictor. This decoupling of maternal body weight from offspring pit expressions resembles a pattern documented in cross-fostering studies (e.g., [58]) indicating that the transmission of this trait cannot be explained solely by maternal size. Instead, these findings strongly suggest a specific genetic component underlying the development of mycangial pits.

## 5. Conclusions

Our study demonstrates that the mycangial pits number in *P. quercivorus* is primarily a response to environmental temperature. Although warmer conditions (26 °C) boost adult activity and offspring numbers, they lead to a marked reduction in individual pit numbers. In contrast, cooler environments (18 °C) produce offspring with higher pit numbers and greater body weight, likely due to a combination of prolonged larval feeding and lower metabolic demands. Statistical modeling confirms this environmental dominance, showing that temperature’s influence is 2.6 times stronger than maternal inheritance. Crucially, the number of mycangial pits in *P. quercivorus* is not merely a byproduct of body size but is a heritable trait subject to both environmental modulation and independent genetic control, potentially reflecting an evolutionary adaptation for maintaining symbiotic efficiency. Finally, total reproductive failure at 30 °C suggests that the critical upper thermal threshold is lower than 30 °C. Such failure may stem from high-temperature interference with essential symbiotic fungal resources or direct physiological disruptions during larval development.

## Figures and Tables

**Table 1:** Results of the Poisson GLM evaluating the effects of temperature and number of mycangium pits groups (No. of pits grp) on the number of emerged females per gallery (offset = log of successful galleries).

Predictor	Coefficient	Std. Error	z Value	*p*-Value
Intercept	3.9413	0.0528	74.684	<0.001
Temperature	0.4865	0.0553	8.797	<0.001
No. of pits grp (Small)	–0.8917	0.1258	–7.086	<0.001
Temperature × No. of pits grp (Small)	–1.0780	0.1484	–7.267	<0.001

**Table 2:** Effects of temperature and number of pits group on offspring body weight in *Platypus quercivorus* (Gaussian GLM).

Predictor	Coefficient	Std. Error	t Value	*p* Value
Intercept	1.477	0.004	339.49	<0.001
Temperature	−0.043	0.005	−9.47	<0.001
No. of pits group (Small)	−0.087	0.017	−5.11	<0.001
Temperature × No. of pits grp (Small)	0.009	0.014	0.61	0.543

**Table 3:** Effects of temperature and parental traits (Number of maternal pits, Maternal and Paternal weight) on number of offspring mycangium pits in *Platypus quercivorus* females, using Poisson generalized linear models. Coefficients are shown with standard errors (SE).

ID	Model	Predictor	Coefficient	SE	z Value	*p* Value
1	Temperature	Intercept	2.023	0.014	142.93	<0.001
	AIC = 2696.9	Temperature	−0.083	0.014	−6.04	<0.001
2	No. of maternal pits	Intercept	2.026	0.014	143.62	<0.001
	AIC = 2730.9	No. of maternal pits.	0.019	0.014	1.32	0.187
3	Maternal weight	Intercept	2.026	0.014	143.58	<0.001
	AIC = 2729.0	Maternal weight	0.027	0.014	1.94	0.052
4	Paternal weight	Intercept	2.025	0.014	143.48	<0.001
	AIC = 2723.8	Paternal weight	0.041	0.014	2.99	0.003
5	Temperature + No. of maternal pits	Intercept	2.022	0.014	142.81	<0.001
	AIC = 2693.3	Temperature	−0.088	0.014	−6.36	<0.001
		No. of maternal pits	0.034	0.014	2.36	0.018
6	Temperature + Maternal weight	Intercept	2.022	0.014	142.87	<0.001
	AIC = 2696.8	Temperature	−0.081	0.014	−5.91	<0.001
		Maternal weight	0.020	0.014	1.44	0.149
7	Temperature + Paternal weight	Intercept	2.022	0.014	142.91	<0.001
	AIC = 2697.9	Temperature	−0.077	0.015	−5.30	<0.001
		Paternal weight	0.015	0.015	1.00	0.316

## Data Availability

The data presented in this study are available as Supplementary Materials.

## Supplementary Materials

The following supporting information can be downloaded at: https://www.mdpi.com/article/10.3390/insects17060536/s1. Figure S1: Temporal trajectories of the within-brood mean number of mycangial pits in *Platypus quercivorus*, calculated from female individuals in Table S1; Table S1: Raw individual-level observations of number of mycangial pits, sex, body weight and collection timing; Table S2: Estimated brood-level summaries calculated for each distinct brood, including the mean number of mycangial pits (females only), sex-specific mean body weight, and the number of samples for each sex; Table S3: Individual-level measurements of mycangial pit number (No. pits) and body weight (BW) in female *Platypus quercivorus* offspring produced from parental pairings on *Quercus crispula* logs.
