# Supplementary material for: Effect of Temperature and Genetic Inheritance on the Number of Mycangium Pits in Female Platypus quercivorus (Coleoptera: Curculionidae: Platypodinae)
Source: Insects. 2026 May 22;17(6):536. doi: 10.3390/insects17060536 (PMC13300474; doi:10.3390/insects17060536)
Supplement: Supplementary file 1 [file insects-17-00536-s001.zip › Table_S3.pdf]

Table S3. Individual-level measurements of mycangial pit number (No. pits) and body weight (BW) in female *Platypus quercivorus* offspring produced from parental pairings on *Quercus crispula* logs, with pairings restricted within groups defined by brood-level mean mycangial pit number, and reared under three temperature regimes (18, 22, and 26 °C) at the University of Tokyo Chichibu Kagemori Nursery. Established parental pairs from 'Large' (L) and 'Small' (S) groups, defined based on the temporal trajectories of cumulative mean mycangial pit number across 111 broods (see Figure S1), were reared, and the resulting offspring were incubated from egg to adult under constant temperature regimes of 18 °C, 22 °C, and 26 °C to produce the individuals described in this dataset. Each row represents a single female individual identified by a unique brood ID (e.g., L1, S12), where 'L' and 'S' indicate the parental group, enabling direct lineage tracing to the parental pair. Only females were recorded because the mycangium is a sexually dimorphic trait exclusive to this sex. Morphological variables include the total number of mycangial pits (No. pits), quantified using high-resolution microscopy, and individual body weight (BW), recorded to a precision of 0.0001 mg using a micro-analytical balance.

**Broods**	**No pits**	**BW (mg)**	**Temperature**
L10	8	1.3846	26
L10	10	1.4461	26
L10	6	1.3407	26
L10	7	1.3183	26
L10	8	1.4544	26
L10	6	1.3805	26
L10	7	1.3641	26
L10	7	1.3139	26
L10	6	1.5207	26
L10	9	1.5022	26
L10	7	1.4320	26
L10	9	1.3731	26
L10	8	1.3516	26
L10	6	1.3352	26
L10	8	1.4857	26
L10	6	1.4856	26
L10	8	1.4086	26
L10	6	1.4048	26
L10	6	1.4048	26
L10	8	1.4038	26
L10	7	1.4016	26
L10	10	1.3904	26
L10	7	1.3822	26
L10	7	1.3659	26
L10	6	1.3631	26
L10	8	1.3334	26

L10	7	1.3216	26
L10	9	1.4542	26
L10	8	1.4514	26
L10	8	1.4341	26
L10	8	1.4106	26
L10	7	1.4002	26
L10	8	1.3823	26
L10	7	1.3736	26
L10	8	1.3415	26
L10	8	1.5126	26
L10	6	1.4976	26
L10	7	1.4866	26
L10	7	1.4381	26
L10	7	1.4373	26
L10	9	1.3856	26
L10	7	1.3673	26
L10	7	1.3496	26
L10	10	1.3353	26
L10	6	1.3338	26
L10	8	1.5668	26
L10	6	1.4827	26
L10	7	1.4266	26
L10	6	1.3911	26
L10	7	1.4575	26
L10	6	1.5348	26
L11	6	1.0871	26
L12	8	1.2797	26
L12	8	1.3684	26
L12	9	1.2681	26
L12	7	1.2598	26
L12	8	1.2493	26
L12	9	1.3316	26
L12	8	1.3987	26
L12	7	1.2605	26
L12	8	1.2483	26
L12	8	1.4071	26
L12	8	1.3893	26
L12	10	1.3868	26
L12	7	1.3589	26
L12	6	1.3232	26
L12	8	1.3227	26
L12	8	1.3061	26
L12	6	1.2928	26
L12	7	1.2819	26
L12	6	1.2283	26
L12	6	1.3646	26
L12	7	1.3932	26

L12	8	1.3142	26
L12	7	1.2959	26
L12	7	1.2217	26
L12	7	1.4871	26
L12	7	1.4134	26
L12	6	1.3758	26
L12	7	1.3519	26
L12	6	1.3442	26
L12	9	1.2579	26
L12	7	1.3679	26
L12	9	1.3462	26
L12	7	1.3786	26
L12	7	1.3446	26
L12	9	1.3588	26
L12	8	1.3027	26
L12	8	1.3007	26
L12	7	1.3854	26
L12	6	1.3708	26
L12	7	1.3543	26
L12	7	1.2613	26
L12	7	1.3219	26
L12	6	1.2684	26
L12	9	1.4233	26
L12	6	1.3521	26
L12	7	1.2815	26
L12	6	1.2679	26
L12	6	1.2506	26
L12	7	1.3884	26
L12	8	1.3729	26
L12	7	1.3580	26
L12	7	1.4413	26
L12	8	1.3651	26
L12	7	1.3072	26
L12	8	1.3816	26
L12	6	1.4496	26
L12	6	1.3629	26
L12	6	1.3608	26
L12	7	1.3152	26
L12	7	1.2832	26
L12	7	1.2791	26
L12	6	1.2691	26
L12	6	1.3894	26
L12	6	1.4581	26
L12	6	1.4287	26
L12	6	1.3529	26
L12	6	1.4188	26
L12	8	1.4173	26

L12	8	1.4781	26
L12	6	1.4575	26
L12	7	1.3993	26
L12	9	1.4774	26
L12	7	1.2287	26
L12	6	1.4318	26
L12	8	1.4621	26
L12	9	1.4274	26
L12	7	1.3809	26
L12	9	1.2013	26
L12	7	1.3762	26
L12	8	1.1091	26
L12	8	1.3754	26
L12	6	1.3949	26
L13	9	1.3117	26
L13	6	1.4409	26
L13	6	1.4216	26
L13	6	1.4172	26
L13	7	1.5083	26
L13	8	1.4821	26
L13	7	1.4767	26
L13	7	1.3682	26
L13	8	1.3528	26
L13	8	1.3344	26
L13	8	1.5365	26
L13	7	1.5247	26
L13	8	1.4777	26
L13	6	1.4621	26
L13	8	1.4383	26
L13	6	1.3961	26
L13	6	1.4382	26
L13	8	1.3878	26
L13	6	1.3042	26
L13	7	1.6758	26
L13	8	1.6307	26
L13	6	1.5831	26
L13	6	1.5291	26
L13	7	1.5257	26
L13	7	1.5166	26
L13	7	1.5129	26
L13	6	1.4964	26
L13	9	1.4837	26
L13	6	1.4813	26
L13	7	1.4752	26
L13	6	1.4703	26
L13	6	1.4681	26
L13	6	1.4554	26

L13	6	1.4511	26
L13	7	1.4415	26
L13	8	1.4308	26
L13	6	1.4146	26
L13	9	1.4127	26
L13	7	1.3924	26
L13	6	1.3802	26
L13	6	1.3511	26
L13	6	1.2644	26
L13	7	1.4191	26
L13	7	1.5908	26
L13	9	1.5858	26
L13	7	1.5728	26
L13	6	1.5422	26
L13	7	1.5331	26
L13	8	1.5301	26
L13	7	1.5610	26
L13	6	1.4886	26
L13	6	1.4575	26
L13	6	1.4171	26
L13	6	1.5788	26
L13	8	1.5249	26
L13	8	1.5116	26
L13	7	1.4909	26
L13	7	1.4762	26
L13	6	1.4532	26
L13	8	1.5396	26
L13	8	1.5103	26
L13	8	1.4698	26
L13	7	1.4637	26
L13	9	1.3537	26
L13	6	1.8027	26
L13	7	1.6449	26
L13	7	1.6273	26
L13	7	1.6148	26
L13	9	1.5987	26
L13	6	1.5509	26
L13	10	1.5471	26
L13	8	1.5466	26
L13	7	1.5378	26
L13	6	1.5319	26
L13	6	1.5289	26
L13	7	1.5009	26
L13	8	1.4905	26
L13	7	1.4879	26
L13	7	1.4274	26
L13	7	1.5986	26

L13	8	1.5766	26
L13	8	1.5749	26
L13	9	1.5621	26
L13	8	1.5596	26
L13	6	1.5578	26
L13	6	1.5567	26
L13	8	1.5358	26
L13	9	1.4845	26
L13	7	1.4812	26
L13	7	1.4711	26
L13	6	1.4096	26
L13	8	1.6256	26
L13	6	1.6635	26
L13	7	1.6321	26
L13	7	1.5966	26
L13	8	1.5396	26
L13	8	1.5027	26
L13	6	1.4418	26
L13	7	1.7968	26
L13	6	1.6778	26
L13	7	1.4981	26
L13	6	1.4918	26
L13	7	1.4859	26
L13	6	1.4680	26
L13	6	1.3362	26
L13	7	1.6082	26
L13	6	1.6011	26
L13	8	1.5889	26
L13	9	1.5854	26
L13	8	1.5731	26
L13	9	1.5591	26
L13	10	1.5577	26
L13	8	1.5511	26
L13	8	1.5184	26
L13	7	1.5078	26
L13	6	1.4606	26
L13	6	1.4467	26
L13	5	1.4463	26
L13	7	1.3741	26
L13	7	1.6003	26
L13	7	1.5286	26
L13	7	1.5223	26
L13	6	1.4264	26
L13	6	1.3716	26
L13	8	1.7321	26
L13	7	1.6354	26
L13	6	1.6142	26

L13	7	1.6124	26
L13	8	1.5787	26
L13	7	1.5631	26
L13	7	1.5339	26
L13	7	1.5244	26
L13	6	1.4987	26
L13	7	1.4819	26
L13	6	1.4766	26
L13	6	1.4603	26
L13	8	1.3831	26
L13	6	1.5958	26
L13	7	1.5753	26
L13	9	1.5659	26
L13	6	1.5207	26
L13	9	1.4888	26
L13	7	1.4746	26
L13	8	1.4609	26
L13	7	1.4454	26
L13	6	1.4049	26
L13	6	1.3994	26
L13	8	1.3954	26
L13	9	1.3912	26
L13	8	1.4909	26
L13	6	1.4967	26
L13	8	1.4749	26
L13	7	1.4694	26
L13	6	1.4641	26
L13	6	1.4586	26
L13	8	1.4527	26
L13	6	1.4470	26
L13	6	1.4434	26
L13	6	1.4431	26
L13	6	1.4378	26
L13	7	1.4078	26
L13	6	1.3314	26
L13	7	1.2632	26
L13	6	1.5218	26
L13	9	1.4773	26
L13	7	1.4707	26
L13	6	1.4438	26
L13	9	1.4381	26
L13	7	1.4296	26
L13	6	1.4216	26
L13	6	1.4097	26
L13	9	1.4013	26
L13	9	1.3958	26
L13	6	1.3941	26

L13	6	1.3910	26
L13	9	1.3909	26
L13	6	1.3898	26
L13	6	1.3371	26
L13	7	1.3077	26
L13	6	1.2989	26
L13	8	1.6092	26
L13	7	1.5839	26
L13	10	1.5543	26
L13	6	1.5209	26
L13	9	1.5013	26
L13	6	1.5007	26
L13	7	1.4561	26
L13	6	1.4151	26
L13	5	1.4063	26
L13	6	1.3521	26
L13	9	1.2687	26
L13	6	1.2522	26
L13	7	1.4040	26
L13	7	1.3523	26
L13	6	1.3494	26
L13	6	1.3329	26
L13	6	1.3287	26
L13	6	1.2895	26
L13	6	1.5823	26
L13	8	1.5741	26
L13	7	1.5635	26
L13	7	1.5461	26
L13	8	1.4961	26
L13	6	1.4381	26
L13	6	1.4291	26
L13	6	1.4101	26
L13	6	1.3912	26
L13	6	1.3471	26
L13	6	1.3349	26
L13	6	1.3281	26
L13	7	1.1339	26
L13	6	1.5984	26
L13	6	1.5439	26
L13	7	1.5317	26
L13	7	1.3964	26
L13	6	1.4478	26
L13	6	1.5621	26
L13	7	1.5484	26
L13	6	1.5325	26
L13	6	1.4703	26
L13	7	1.4335	26

L13	8	1.5167	26
L13	8	1.4987	26
L13	6	1.4738	26
L13	6	1.4629	26
L13	6	1.4427	26
L13	7	1.4176	26
L13	8	1.4134	26
L13	6	1.4112	26
L13	7	1.3887	26
L13	6	1.3866	26
L13	7	1.3685	26
L13	7	1.3114	26
L13	8	1.4101	26
L13	7	1.3636	26
L13	6	1.2791	26
L13	7	1.2695	26
L13	6	1.2046	26
S12	6	1.2727	26
S12	6	1.1654	26
S12	6	1.1774	26
S12	7	1.1851	26
S12	6	1.2205	26
S12	6	1.2451	26
S12	6	1.0923	26
L7	10	1.5743	22
L7	9	1.5931	22
L7	8	1.5005	22
L7	10	1.4974	22
L7	6	1.7244	22
L7	10	1.5504	22
L7	9	1.5417	22
L7	10	1.4925	22
L7	8	1.4923	22
L7	9	1.4893	22
L7	8	1.4813	22
L7	10	1.4805	22
L7	10	1.4722	22
L7	9	1.4584	22
L7	7	1.5941	22
L7	10	1.5931	22
L7	10	1.5907	22
L7	8	1.5724	22
L7	10	1.5391	22
L7	10	1.5332	22
L7	10	1.5241	22
L7	10	1.5083	22
L7	10	1.4882	22

L7	8	1.4755	22
L7	10	1.4691	22
L7	10	1.4131	22
L7	7	1.5494	22
L7	9	1.5339	22
L7	9	1.5247	22
L7	8	1.4801	22
L7	10	1.3428	22
L7	11	1.3572	22
L7	9	1.6648	22
L7	7	1.6326	22
L7	8	1.6082	22
L7	9	1.5992	22
L7	8	1.5283	22
L7	8	1.4969	22
L7	8	1.6398	22
L7	10	1.5847	22
L7	8	1.5772	22
L7	8	1.5652	22
L7	8	1.5338	22
L7	9	1.5271	22
L7	9	1.5154	22
L7	7	1.6623	22
L7	8	1.6540	22
L7	8	1.6098	22
L7	8	1.5982	22
L7	10	1.5877	22
L7	8	1.5561	22
L7	7	1.5886	22
L7	9	1.5431	22
L7	7	1.5549	22
L7	9	1.5511	22
L7	6	1.5482	22
L7	8	1.5427	22
L7	10	1.5627	22
L7	10	1.5553	22
L7	6	1.6291	22
L7	9	1.6265	22
L7	9	1.5701	22
L7	7	1.5973	22
L7	8	1.5852	22
L7	6	1.5603	22
L7	8	1.5323	22
L7	7	1.5301	22
L7	6	1.4493	22
L7	6	1.4376	22
L7	6	1.4122	22

L7	8	1.4097	22
L7	9	1.6172	22
L7	8	1.5771	22
L7	10	1.5633	22
L7	8	1.5281	22
L7	9	1.6661	22
L7	7	1.7311	22
L7	8	1.5104	22
L7	8	1.5629	22
L7	10	1.6233	22
L7	8	1.6166	22
L7	8	1.3534	22
L9	10	1.5608	22
L9	9	1.5373	22
L9	8	1.5068	22
L9	6	1.6781	22
L9	10	1.5913	22
L9	10	1.5686	22
L9	8	1.5200	22
L9	6	1.6464	22
L9	7	1.5851	22
L9	6	1.5651	22
L9	8	1.5319	22
L9	6	1.5314	22
L9	8	1.4597	22
L9	8	1.6321	22
L9	8	1.5167	22
L9	7	1.4855	22
L9	7	1.5708	22
L9	7	1.5683	22
L9	7	1.5433	22
L9	9	1.6791	22
L9	8	1.5863	22
L9	7	1.5693	22
L9	6	1.5411	22
L9	6	1.5373	22
L9	6	1.5086	22
L9	8	1.5051	22
L9	7	1.4968	22
L9	6	1.4583	22
L9	6	1.4531	22
L9	6	1.2995	22
L9	7	1.5737	22
L9	7	1.5681	22
L9	7	1.4944	22
L9	10	1.5652	22
L9	9	1.4934	22

L9	8	1.6071	22
L9	8	1.4656	22
S7	8	1.4928	22
S7	9	1.5591	22
S7	8	1.5854	22
S7	7	1.5801	22
S7	6	1.5078	22
S7	6	1.5184	22
S7	9	1.4487	22
S7	8	1.4184	22
S7	9	1.3741	22
S7	7	1.4463	22
S7	7	1.5471	22
S7	7	1.4905	22
S7	9	1.8027	22
S7	8	1.5009	22
S7	9	1.4274	22
S7	9	1.6420	22
S7	7	1.5387	22
S7	8	1.5549	22
S7	7	1.5766	22
S7	8	1.5851	22
S7	6	1.5212	22
S7	9	1.5471	22
S7	8	1.6648	22
S7	6	1.4966	22
S7	6	1.4902	22
S7	9	1.4257	22
S7	6	1.5677	22
S7	7	1.6393	22
S7	6	1.5934	22
S7	7	1.5908	22
S7	8	1.4109	22
S7	8	1.4056	22
S7	8	1.3931	22
S7	9	1.3894	22
S7	9	1.3534	22
S7	9	1.3784	22
S7	10	1.1254	22
S7	8	1.3824	22
S7	6	1.4491	22
S7	7	1.4238	22
S7	9	1.4063	22
S7	9	0.8994	22
S7	8	1.1837	22
S7	7	1.4147	22
S7	8	1.2571	22

S7	7	1.1297	22
S7	7	1.3885	22
S7	6	1.3989	22
S7	8	1.2797	22
S7	8	1.3212	22
S8	8	1.4927	22
L1	9	1.5321	18
L1	10	1.5273	18
L1	12	1.6073	18
L1	11	1.5892	18
L1	9	1.5548	18
L1	9	1.5419	18
L1	11	1.5720	18
L1	9	1.5621	18
L1	12	1.5584	18
L1	10	1.5306	18
L1	10	1.5174	18
L1	9	1.5740	18
L1	10	1.5686	18
L1	10	1.5489	18
L1	7	1.5311	18
L1	8	1.5631	18
L1	10	1.5614	18
L1	10	1.4867	18
L1	9	1.4826	18
L1	10	1.6362	18
L1	10	1.5404	18
L1	9	1.5229	18
L1	9	1.5168	18
L1	9	1.4956	18
L1	10	1.6913	18
L1	8	1.6171	18
L1	8	1.6135	18
L1	11	1.5934	18
L1	10	1.6388	18
L1	10	1.6167	18
L1	8	1.6163	18
L1	10	1.5731	18
L1	12	1.6744	18
L1	9	1.5387	18
L1	10	1.6175	18
L1	9	1.5258	18
L1	9	1.5971	18
L1	8	1.5267	18
L1	7	1.5187	18
L1	8	1.5883	18
L1	7	1.5382	18

L1	9	1.6064	18
L1	10	1.5607	18
L1	6	1.5338	18
L1	6	1.5113	18
L1	6	1.4764	18
L1	6	1.4244	18
L1	6	1.3522	18
L1	10	1.5117	18
L1	6	1.4504	18
L1	8	1.3783	18
L1	8	1.5167	18
L1	8	1.5917	18
L1	9	1.5604	18
L1	9	1.5016	18
L1	7	1.4101	18
L3	10	1.4366	18
L3	10	1.4371	18
L3	10	1.3726	18
L3	12	1.3941	18
L3	9	1.3931	18
L3	11	1.3893	18
L3	8	1.4214	18
L3	10	1.4927	18
L3	9	1.4153	18
L3	10	1.3913	18
L3	9	1.4778	18
L3	10	1.3882	18
L3	12	1.5185	18
L3	10	1.5936	18
L3	9	1.6432	18
L3	9	1.4887	18
L3	10	1.5184	18
L3	9	1.5364	18
L3	9	1.4689	18
L3	8	1.4131	18
L3	10	1.3409	18
S2	7	1.4492	18
S2	10	1.4535	18
S2	10	1.3411	18
S2	6	1.4238	18
S2	7	1.4231	18
S2	9	1.4109	18
S2	9	1.3784	18
S2	8	1.4473	18
S2	8	1.4027	18
S2	7	1.3724	18
S2	6	1.3647	18

S2	8	1.3638	18
S2	7	1.3812	18
S2	8	1.4645	18
S2	7	1.4091	18
S2	9	1.3962	18
S2	8	1.4260	18
S2	8	1.3750	18
S2	8	1.3417	18
S2	11	1.4826	18
S2	9	1.3833	18
S2	8	1.3662	18
S2	9	1.3999	18
S2	8	1.5161	18
S2	7	1.4271	18
S2	9	1.3103	18
S2	7	1.4386	18
S2	6	1.4314	18
S2	7	1.3671	18
S2	9	1.5301	18
S2	6	1.4008	18
S2	6	1.5984	18
S2	8	1.5117	18
S2	6	1.4492	18
S2	8	1.4623	18
S2	6	1.4001	18
S2	6	1.5024	18
