# Supplementary material for: Effect of Temperature and Genetic Inheritance on the Number of Mycangium Pits in Female Platypus quercivorus (Coleoptera: Curculionidae: Platypodinae)
Source: Insects. 2026 May 22;17(6):536. doi: 10.3390/insects17060536 (PMC13300474; doi:10.3390/insects17060536)
Supplement: Supplementary file 1 [file insects-17-00536-s001.zip › Table_S2.pdf]

Table S2. Estimated brood-level summaries were calculated for each distinct brood, including the mean number of mycangial pits (females only), sex-specific mean body weight, and the number of samples for each sex for *Platypus quercivorus* individuals collected from *Quercus serrata* host trees using emergence traps (see Figure 1) at the University of Tokyo Tanashi Forest in 2023. Broods were then classified into three size groups based on the brood-level mean number of mycangial pits: small (≤ 6.57), medium (> 6.57 and < 7.90), and large (≥ 7.90), determined by visual inspection of the relative stability of cumulative mean trajectories in Figure S1. These brood-level mean values were then assigned as proxy estimates of maternal pit number and sex-specific parental body weights to individuals that subsequently emerged from the same broods following classification into large and small groups for use in pairing experiments. Some brood IDs are absent because no individuals were captured for those broods during the sampling period. No data imputation was performed; all values represent strictly observed biological measurements used to assess the influence of parental traits on the subsequent offspring generation.

Brood ID	Mean No. Pits	Mean BW (Female)	*N* (Female)	Mean BW (Male)	*N* (Male)	Size group
1	8.34	1.3630	44	1.3725	52	Large
3	7.12	1.1574	8	1.1735	16	Medium
4	7.16	1.4586	43	1.4108	36	Medium
5	7.37	1.3124	30	1.2349	46	Medium
7	6.67	1.2202	12	1.1832	11	Medium
8	7.93	1.2221	15	1.2746	20	Large
10	6.00	1.0346	1	1.0073	6	Small
11	7.00	1.2944	26	1.2006	29	Medium
12	7.00	1.3176	29	1.3152	45	Medium
13	7.77	1.3361	31	1.2739	22	Medium
14	6.62	1.2701	13	1.2399	25	Medium
15	6.83	1.3283	18	1.3063	25	Medium
16	6.86	1.2457	7	1.2158	6	Medium
17	7.36	1.2857	14	1.2549	4	Medium
18	6.38	1.2418	13	1.1886	16	Small
19	6.67	1.3553	12	1.3313	16	Medium
20	7.29	1.2742	21	1.3318	37	Medium
21	6.90	1.2985	29	1.3207	25	Medium
22	7.85	1.3268	13	1.2260	52	Medium
24	7.22	1.3228	27	1.3254	23	Medium
25	6.75	1.1311	4	1.3217	1	Medium
26	7.14	1.3821	37	1.3841	49	Medium
27	7.65	1.2502	17	1.2496	11	Medium
29	6.86	1.3449	22	1.2974	26	Medium
31	7.60	1.2987	30	1.3291	28	Medium
32	7.75	1.3348	8	1.2878	26	Medium

34	7.79	1.2247	19	1.3099	18	Medium
35	7.39	1.2887	18	1.2234	29	Medium
36	7.17	1.1901	30	1.2496	39	Medium
37	7.62	1.3063	32	1.2949	31	Medium
38	6.44	1.2882	27	1.2387	40	Small
41	7.00	1.0421	1	1.0879	5	Medium
42	6.50	1.1583	12	1.1852	19	Small
43	7.15	1.3013	13	1.2469	19	Medium
46	6.67	1.1383	6	1.2036	10	Medium
48	7.43	1.2555	7	1.2715	17	Medium
49	NA	NA	NA	1.0814	4	NA
50	6.64	1.3062	25	1.2419	13	Medium
51	6.75	1.1863	8	1.2450	4	Medium
52	8.00	1.1780	1	1.2035	2	Large
53	7.09	1.2214	22	1.2055	27	Medium
54	6.88	1.2003	17	1.2364	10	Medium
55	7.14	1.2532	22	1.2972	16	Medium
56	8.78	1.2466	9	1.2457	21	Large
57	7.24	1.2668	21	1.2708	16	Medium
59	6.88	1.1127	8	1.0780	4	Medium
60	8.40	1.7573	68	1.6369	65	Large
61	7.04	1.3495	28	1.2641	32	Medium
62	6.87	1.2265	15	1.1648	7	Medium
63	7.30	1.2849	23	1.2368	24	Medium
64	6.76	1.3781	37	1.3567	42	Medium
65	6.85	1.2624	13	1.2343	15	Medium
66	6.77	1.2395	13	1.1515	29	Medium
67	6.87	1.2978	30	1.2878	41	Medium
69	7.29	1.2356	14	1.2481	26	Medium
70	6.41	1.2975	17	1.3081	30	Small
71	6.67	1.1766	6	1.2084	11	Medium
73	6.71	1.2884	17	1.2479	17	Medium
74	7.33	1.3427	46	1.2501	36	Medium
75	7.47	1.2918	40	1.2707	41	Medium
76	6.64	1.2294	22	1.1095	19	Medium
77	6.62	1.1764	8	1.1991	28	Medium
78	7.17	1.3235	30	1.3497	24	Medium
79	6.00	1.1815	3	1.1736	4	Small
81	8.00	1.2931	13	1.2564	30	Large
82	NA	NA	NA	1.2203	1	NA
83	6.86	1.1688	7	1.2297	10	Medium
84	7.33	1.2516	6	1.2390	5	Medium
85	NA	NA	NA	1.2017	1	NA
86	7.39	1.2662	18	1.1967	28	Medium
87	6.97	1.3492	30	1.3506	36	Medium
88	7.05	1.2970	20	1.2322	34	Medium
89	7.00	1.1723	2	NA	NA	Medium

90	7.32	1.3762	50	1.3509	69	Medium
91	6.88	1.4449	40	1.4493	20	Medium
92	7.00	1.2156	2	1.3421	4	Medium
96	7.00	1.2477	15	1.1239	16	Medium
97	7.03	1.2214	34	1.2058	35	Medium
98	6.69	1.2096	16	1.1800	11	Medium
99	7.59	1.1939	17	1.2003	22	Medium
100	6.50	1.1764	2	1.3332	1	Small
101	7.80	1.2579	25	1.4014	24	Medium
102	7.64	1.4882	42	1.4755	41	Medium
103	6.50	0.9846	2	1.0154	2	Small
104	6.86	1.1633	7	1.1771	9	Medium
105	7.33	1.2325	9	1.2224	14	Medium
106	8.11	1.3414	35	1.3739	28	Large
107	7.26	1.2285	27	1.2669	21	Medium
200	6.88	1.4485	32	1.4639	61	Medium
201	7.10	1.2379	10	1.2432	10	Medium
202	6.67	1.2410	3	1.1800	5	Medium
204	6.76	1.4013	38	1.3862	68	Medium
205	6.36	1.3019	22	1.3580	23	Small
206	6.46	1.3720	28	1.4672	30	Small
207	6.88	1.3608	42	1.4763	40	Medium
208	6.56	1.3417	9	1.4890	14	Small
209	6.00	1.5046	1	1.4715	10	Small
210	6.36	1.3561	22	1.3561	34	Small
211	6.69	1.3628	26	1.3598	43	Medium
212	7.29	1.3364	24	1.3239	34	Medium
213	6.45	1.3370	20	1.2499	28	Small
214	NA	NA	NA	1.1778	2	NA
215	7.49	1.4171	45	1.4543	39	Medium
216	6.50	1.4804	2	NA	NA	Small
217	8.58	1.3009	19	1.2914	22	Large
218	6.67	1.3475	24	1.3189	26	Medium
219	6.81	1.3740	27	1.2606	45	Medium
220	6.47	1.2852	15	1.2930	34	Small
221	NA	NA	NA	1.3673	1	NA
222	6.97	1.3439	30	1.2891	42	Medium
223	6.58	1.3317	36	1.2678	32	Medium
224	6.57	1.3419	42	1.2902	44	Small
225	7.50	1.3840	2	NA	NA	Medium
226	7.40	1.3163	52	1.2683	39	Medium
227	7.13	1.2429	45	1.2703	51	Medium
228	6.86	1.2917	14	1.2732	31	Medium
