# Supplementary material for: Effect of Temperature and Genetic Inheritance on the Number of Mycangium Pits in Female Platypus quercivorus (Coleoptera: Curculionidae: Platypodinae)
Source: Insects. 2026 May 22;17(6):536. doi: 10.3390/insects17060536 (PMC13300474; doi:10.3390/insects17060536)
Supplement: Supplementary file 1 [file insects-17-00536-s001.zip › Table_S1.pdf]

Table S1. Raw individual-level observations of number of mycangial pits, sex,body weight and collection timing for *Platypus quercivorus* individuals collected from *Quercus serrata* host trees using emergence traps (see Figure 1) at the University of Tokyo Tanashi Forest. This dataset provides the complete record of observations used to analyze mycangial pit variation and establish brood-size classifications, collected between May and July 2023 from *i*nfested *Q. serrata* trees. Organized in long format, each row represents a single individual collected from a trap on a given sampling date, where Brood ID indicates the unique gallery and serves to identify repeated samples from the same brood over time. For each individual, sexing was performed based on the presence or absence of mycangial pits on the pronotum using a magnifier, and body weight was measured using a highly sensitive microbalance to the nearest 0.0001 mg. The number of mycangial pits (No. pits) was recorded as a non-negative integer for all specimens; due to the sexual dimorphism of the species, these structures are present only in females, and males are consistently assigned a value of 0. Sampling dates are reported as Julian dates (Days 143–233) to facilitate further analysis, with collections occurring on weekdays to the exclusion of weekends and holidays.

Individual ID	Brood ID	Julian date	Sex	No pits	Body weight (mg)
1	52	143	Male	0	1.2008
2	87	149	Male	0	1.3651
3	88	149	Male	0	1.2827
4	22	151	Male	0	1.3832
5	205	151	Male	0	1.4148
6	213	151	Male	0	1.3151
7	224	151	Male	0	1.2775
8	34	156	Male	0	1.2672
9	221	158	Male	0	1.3673
10	222	158	Male	0	1.3103
11	5	160	Male	0	1.3554
12	38	160	Male	0	1.2370
13	38	160	Male	0	1.3269
14	1	163	Male	0	1.2827
15	1	163	Male	0	1.5405
16	3	163	Male	0	0.8698
17	3	163	Male	0	1.2024
18	3	163	Male	0	1.2104
19	3	163	Male	0	1.3120
20	5	163	Female	8	1.2652
21	5	163	Male	0	1.2403
22	5	163	Male	0	1.3742
23	8	163	Male	0	1.2307

24	12	163	Male	0	1.2199
25	12	163	Male	0	1.2318
26	12	163	Male	0	1.2433
27	12	163	Male	0	1.2867
28	12	163	Male	0	1.3426
29	12	163	Male	0	1.3446
30	12	163	Male	0	1.3826
31	12	163	Male	0	1.3846
32	15	163	Male	0	1.2519
33	15	163	Male	0	1.2689
34	15	163	Male	0	1.3915
35	18	163	Male	0	1.0776
36	19	163	Male	0	1.3123
37	21	163	Male	0	1.2184
38	21	163	Male	0	1.3430
39	22	163	Male	0	1.0110
40	24	163	Male	0	1.1768
41	24	163	Male	0	1.4392
42	35	163	Female	7	1.4076
43	35	163	Male	0	1.2769
44	35	163	Male	0	1.2987
45	38	163	Male	0	1.2759
46	38	163	Male	0	1.3101
47	42	163	Female	6	1.2342
48	42	163	Male	0	1.0873
49	43	163	Male	0	1.3940
50	48	163	Male	0	1.2719
51	49	163	Male	0	1.0240
52	49	163	Male	0	1.0808
53	53	163	Male	0	1.2507
54	55	163	Female	9	1.3404
55	55	163	Male	0	1.3367
56	56	163	Male	0	1.2847
57	56	163	Male	0	1.3344
58	63	163	Female	8	1.2751
59	63	163	Male	0	1.2554
60	63	163	Male	0	1.2623
61	63	163	Male	0	1.3239
62	63	163	Male	0	1.3683
63	67	163	Male	0	1.1691
64	67	163	Male	0	1.2056
65	67	163	Male	0	1.3439
66	67	163	Male	0	1.3719
67	67	163	Male	0	1.4228

68	75	163	Male	0	1.2887
69	75	163	Male	0	1.3272
70	77	163	Male	0	1.2849
71	78	163	Male	0	1.3431
72	78	163	Male	0	1.3656
73	87	163	Male	0	1.4404
74	88	163	Male	0	1.2492
75	90	163	Male	0	1.2053
76	90	163	Male	0	1.3617
77	90	163	Male	0	1.3651
78	97	163	Male	0	1.2567
79	202	163	Male	0	1.2077
80	202	163	Male	0	1.2418
81	204	163	Male	0	1.3599
82	204	163	Male	0	1.4884
83	206	163	Male	0	1.4856
84	206	163	Male	0	1.4871
85	207	163	Male	0	1.3740
86	207	163	Male	0	1.5372
87	209	163	Male	0	1.3678
88	209	163	Male	0	1.4993
89	211	163	Male	0	1.2311
90	211	163	Male	0	1.3606
91	211	163	Male	0	1.3678
92	211	163	Male	0	1.3729
93	211	163	Male	0	1.3765
94	211	163	Male	0	1.3796
95	211	163	Male	0	1.4071
96	211	163	Male	0	1.4440
97	211	163	Male	0	1.4445
98	217	163	Male	0	1.4108
99	219	163	Male	0	1.3075
100	223	163	Male	0	1.3179
101	224	163	Male	0	1.1480
102	224	163	Male	0	1.2236
103	1	165	Male	0	1.2124
104	1	165	Male	0	1.2249
105	1	165	Male	0	1.4369
106	1	165	Male	0	1.4527
107	3	165	Female	8	1.1990
108	3	165	Male	0	0.9392
109	3	165	Male	0	1.2547
110	4	165	Female	9	1.5159
111	4	165	Female	9	1.5455

112	4	165	Female	9	1.5605
113	5	165	Female	9	1.4541
114	5	165	Male	0	1.2351
115	7	165	Male	0	1.2011
116	8	165	Male	0	1.2147
117	8	165	Male	0	1.2941
118	8	165	Male	0	1.3542
119	10	165	Male	0	0.7580
120	11	165	Female	8	1.3157
121	11	165	Male	0	1.3988
122	12	165	Female	7	1.3438
123	12	165	Female	9	1.3409
124	12	165	Male	0	1.2526
125	12	165	Male	0	1.2967
126	12	165	Male	0	1.4259
127	13	165	Male	0	1.3277
128	14	165	Female	6	1.2241
129	14	165	Female	9	1.3143
130	14	165	Male	0	1.2043
131	14	165	Male	0	1.2081
132	14	165	Male	0	1.3149
133	15	165	Male	0	1.0337
134	19	165	Male	0	1.1528
135	19	165	Male	0	1.4138
136	21	165	Female	8	1.2281
137	21	165	Male	0	1.3256
138	22	165	Male	0	1.3389
139	24	165	Male	0	1.4066
140	29	165	Female	8	1.2839
141	29	165	Male	0	1.2766
142	29	165	Male	0	1.3040
143	35	165	Male	0	0.9918
144	37	165	Female	10	1.3291
145	37	165	Male	0	1.3095
146	38	165	Female	8	1.3377
147	38	165	Male	0	1.2487
148	38	165	Male	0	1.3568
149	42	165	Female	6	1.0673
150	42	165	Male	0	1.1925
151	42	165	Male	0	1.2631
152	46	165	Male	0	1.1702
153	48	165	Male	0	1.4132
154	49	165	Male	0	1.0868
155	53	165	Female	7	1.2394

156	53	165	Male	0	1.2390
157	55	165	Male	0	1.3377
158	56	165	Male	0	1.2661
159	56	165	Male	0	1.2696
160	63	165	Male	0	1.0857
161	64	165	Male	0	1.4199
162	64	165	Male	0	1.4410
163	66	165	Male	0	1.2043
164	66	165	Male	0	1.2412
165	74	165	Male	0	0.9668
166	74	165	Male	0	1.3574
167	86	165	Male	0	1.3426
168	88	165	Male	0	1.3719
169	97	165	Female	9	1.2413
170	204	165	Male	0	1.3643
171	204	165	Male	0	1.5076
172	205	165	Female	7	1.4015
173	205	165	Male	0	1.4437
174	206	165	Male	0	1.4230
175	206	165	Male	0	1.5171
176	209	165	Male	0	1.4882
177	209	165	Male	0	1.5794
178	209	165	Male	0	1.5815
179	211	165	Male	0	1.2952
180	211	165	Male	0	1.3209
181	211	165	Male	0	1.3248
182	211	165	Male	0	1.3563
183	211	165	Male	0	1.3875
184	211	165	Male	0	1.4249
185	211	165	Male	0	1.4687
186	215	165	Male	0	1.3632
187	215	165	Male	0	1.4818
188	219	165	Male	0	1.2693
189	219	165	Male	0	1.3069
190	220	165	Male	0	1.1706
191	220	165	Male	0	1.4214
192	222	165	Male	0	1.2942
193	222	165	Male	0	1.3751
194	223	165	Male	0	1.3801
195	224	165	Male	0	1.1869
196	226	165	Male	0	1.1650
197	226	165	Male	0	1.1920
198	226	165	Male	0	1.2916
199	226	165	Male	0	1.4106

200	227	165	Male	0	1.1585
201	227	165	Male	0	1.2071
202	227	165	Male	0	1.2212
203	227	165	Male	0	1.2370
204	1	167	Male	0	1.2954
205	1	167	Male	0	1.3116
206	1	167	Male	0	1.3458
207	1	167	Male	0	1.3773
208	1	167	Male	0	1.4352
209	1	167	Male	0	1.4382
210	1	167	Male	0	1.5191
211	1	167	Male	0	1.5881
212	3	167	Male	0	0.7461
213	3	167	Male	0	1.2333
214	3	167	Male	0	1.2817
215	4	167	Female	7	1.5713
216	4	167	Male	0	1.3272
217	5	167	Male	0	0.8690
218	5	167	Male	0	1.1116
219	5	167	Male	0	1.3984
220	5	167	Male	0	1.4368
221	8	167	Male	0	1.1929
222	10	167	Male	0	0.8705
223	10	167	Male	0	1.1264
224	11	167	Female	7	1.1328
225	11	167	Male	0	1.0853
226	11	167	Male	0	1.4146
227	12	167	Male	0	1.2334
228	12	167	Male	0	1.2437
229	12	167	Male	0	1.2646
230	12	167	Male	0	1.2654
231	12	167	Male	0	1.3329
232	12	167	Male	0	1.3358
233	12	167	Male	0	1.3546
234	12	167	Male	0	1.3603
235	12	167	Male	0	1.3749
236	12	167	Male	0	1.3905
237	12	167	Male	0	1.4221
238	13	167	Male	0	1.2008
239	13	167	Male	0	1.3324
240	14	167	Male	0	1.1956
241	14	167	Male	0	1.2879
242	14	167	Male	0	1.3074
243	14	167	Male	0	1.3544

244	15	167	Male	0	1.2233
245	15	167	Male	0	1.3623
246	16	167	Male	0	1.1823
247	18	167	Male	0	1.0731
248	19	167	Male	0	1.3636
249	19	167	Male	0	1.4149
250	22	167	Female	6	1.4877
251	22	167	Female	9	1.3287
252	22	167	Male	0	1.2635
253	22	167	Male	0	1.3248
254	22	167	Male	0	1.3912
255	24	167	Male	0	1.3237
256	26	167	Male	0	1.3120
257	26	167	Male	0	1.3986
258	29	167	Female	8	1.2807
259	29	167	Female	8	1.3584
260	29	167	Male	0	1.3286
261	31	167	Female	7	1.2279
262	31	167	Male	0	1.2421
263	32	167	Male	0	1.2057
264	32	167	Male	0	1.2827
265	32	167	Male	0	1.3261
266	32	167	Male	0	1.3333
267	34	167	Female	9	1.2813
268	34	167	Male	0	1.3302
269	34	167	Male	0	1.3449
270	35	167	Female	6	1.3190
271	35	167	Male	0	1.2369
272	36	167	Male	0	1.1356
273	36	167	Male	0	1.2030
274	36	167	Male	0	1.2193
275	36	167	Male	0	1.2592
276	37	167	Female	9	1.2236
277	37	167	Male	0	1.2347
278	37	167	Male	0	1.2894
279	37	167	Male	0	1.3029
280	37	167	Male	0	1.3097
281	37	167	Male	0	1.3286
282	37	167	Male	0	1.3315
283	37	167	Male	0	1.3473
284	37	167	Male	0	1.3503
285	37	167	Male	0	1.3826
286	38	167	Male	0	1.3967
287	42	167	Male	0	1.1878

288	42	167	Male	0	1.3175
289	43	167	Female	7	1.3398
290	43	167	Female	9	1.3819
291	43	167	Male	0	1.3128
292	43	167	Male	0	1.3463
293	46	167	Female	6	1.1221
294	48	167	Female	7	1.2223
295	48	167	Female	7	1.2791
296	48	167	Female	8	1.3456
297	48	167	Male	0	1.2051
298	48	167	Male	0	1.2755
299	48	167	Male	0	1.3210
300	53	167	Male	0	1.2337
301	53	167	Male	0	1.2727
302	55	167	Female	6	1.2954
303	55	167	Female	8	1.2703
304	55	167	Male	0	1.1733
305	55	167	Male	0	1.1942
306	55	167	Male	0	1.3298
307	55	167	Male	0	1.3693
308	56	167	Male	0	1.3226
309	57	167	Male	0	1.2608
310	57	167	Male	0	1.2820
311	57	167	Male	0	1.3246
312	60	167	Male	0	1.6241
313	60	167	Male	0	1.7633
314	61	167	Male	0	1.3804
315	63	167	Female	6	1.2194
316	63	167	Female	7	1.3359
317	63	167	Female	8	1.3192
318	63	167	Female	9	1.2685
319	63	167	Female	9	1.3161
320	63	167	Female	10	1.2687
321	63	167	Male	0	1.1005
322	63	167	Male	0	1.1523
323	63	167	Male	0	1.3292
324	64	167	Male	0	1.3164
325	64	167	Male	0	1.4156
326	64	167	Male	0	1.4242
327	66	167	Male	0	1.1741
328	69	167	Male	0	1.2388
329	69	167	Male	0	1.3176
330	70	167	Male	0	1.2716
331	70	167	Male	0	1.2910

332	70	167	Male	0	1.3091
333	71	167	Male	0	1.1609
334	73	167	Male	0	1.0641
335	73	167	Male	0	1.2597
336	73	167	Male	0	1.4366
337	74	167	Female	6	1.2637
338	74	167	Female	8	1.3354
339	74	167	Male	0	1.0948
340	74	167	Male	0	1.2979
341	74	167	Male	0	1.3099
342	75	167	Female	6	1.3431
343	75	167	Female	9	1.2493
344	75	167	Female	10	1.3619
345	77	167	Male	0	1.1253
346	78	167	Female	6	1.2751
347	78	167	Female	6	1.3442
348	78	167	Female	8	1.3553
349	78	167	Female	8	1.3764
350	78	167	Female	9	1.3599
351	78	167	Female	11	1.3697
352	78	167	Male	0	1.3445
353	78	167	Male	0	1.3477
354	78	167	Male	0	1.3831
355	78	167	Male	0	1.3962
356	81	167	Male	0	1.3842
357	82	167	Male	0	1.2203
358	86	167	Male	0	1.2374
359	86	167	Male	0	1.2503
360	87	167	Female	6	1.3091
361	87	167	Female	9	1.2941
362	87	167	Male	0	1.2813
363	87	167	Male	0	1.3452
364	87	167	Male	0	1.3816
365	87	167	Male	0	1.3917
366	87	167	Male	0	1.3951
367	87	167	Male	0	1.3966
368	87	167	Male	0	1.4038
369	87	167	Male	0	1.4094
370	88	167	Female	7	1.1968
371	88	167	Male	0	1.0942
372	88	167	Male	0	1.3124
373	88	167	Male	0	1.3378
374	88	167	Male	0	1.3865
375	90	167	Male	0	1.1561

376	90	167	Male	0	1.3901
377	90	167	Male	0	1.4138
378	90	167	Male	0	1.4238
379	90	167	Male	0	1.4418
380	90	167	Male	0	1.4428
381	90	167	Male	0	1.4728
382	90	167	Male	0	1.4846
383	90	167	Male	0	1.5418
384	91	167	Female	7	1.4581
385	91	167	Female	8	1.3784
386	91	167	Female	8	1.5332
387	91	167	Male	0	1.4833
388	91	167	Male	0	1.5144
389	91	167	Male	0	1.5213
390	91	167	Male	0	1.5421
391	97	167	Female	6	1.1829
392	101	167	Male	0	1.3986
393	101	167	Male	0	1.4136
394	101	167	Male	0	1.4362
395	101	167	Male	0	1.5136
396	102	167	Male	0	1.4123
397	102	167	Male	0	1.4268
398	102	167	Male	0	1.4446
399	102	167	Male	0	1.4520
400	102	167	Male	0	1.4542
401	102	167	Male	0	1.4764
402	103	167	Male	0	0.9961
403	104	167	Male	0	1.1698
404	104	167	Male	0	1.1843
405	200	167	Male	0	1.4118
406	200	167	Male	0	1.4719
407	200	167	Male	0	1.4911
408	200	167	Male	0	1.5236
409	200	167	Male	0	1.5424
410	200	167	Male	0	1.5474
411	200	167	Male	0	1.6524
412	204	167	Male	0	1.3899
413	204	167	Male	0	1.4723
414	204	167	Male	0	1.4767
415	204	167	Male	0	1.5397
416	205	167	Male	0	1.3062
417	205	167	Male	0	1.4029
418	206	167	Female	7	1.3517
419	206	167	Female	8	1.4684

420	206	167	Male	0	1.3561
421	206	167	Male	0	1.4490
422	206	167	Male	0	1.5204
423	206	167	Male	0	1.5516
424	206	167	Male	0	1.5554
425	207	167	Male	0	1.3792
426	207	167	Male	0	1.3904
427	207	167	Male	0	1.4629
428	208	167	Male	0	1.4946
429	208	167	Male	0	1.5231
430	209	167	Male	0	1.4639
431	209	167	Male	0	1.5444
432	210	167	Male	0	1.2590
433	210	167	Male	0	1.3597
434	210	167	Male	0	1.3845
435	211	167	Male	0	1.2468
436	211	167	Male	0	1.2541
437	212	167	Male	0	1.2408
438	212	167	Male	0	1.3131
439	212	167	Male	0	1.3664
440	212	167	Male	0	1.3943
441	213	167	Female	8	1.3523
442	213	167	Male	0	1.3326
443	213	167	Male	0	1.3388
444	215	167	Female	9	1.4549
445	215	167	Male	0	1.3632
446	215	167	Male	0	1.4132
447	215	167	Male	0	1.4818
448	218	167	Male	0	1.2677
449	218	167	Male	0	1.3377
450	219	167	Female	7	1.5267
451	219	167	Male	0	1.1910
452	219	167	Male	0	1.2220
453	219	167	Male	0	1.2592
454	222	167	Male	0	1.1658
455	222	167	Male	0	1.2910
456	223	167	Male	0	1.2072
457	223	167	Male	0	1.2437
458	223	167	Male	0	1.2777
459	224	167	Male	0	1.2281
460	224	167	Male	0	1.2951
461	224	167	Male	0	1.4022
462	226	167	Female	8	1.4154
463	226	167	Female	9	1.1818

464	226	167	Female	9	1.4516
465	226	167	Male	0	1.2299
466	226	167	Male	0	1.3553
467	227	167	Male	0	1.1480
468	227	167	Male	0	1.2199
469	227	167	Male	0	1.2226
470	227	167	Male	0	1.2346
471	227	167	Male	0	1.2964
472	227	167	Male	0	1.3065
473	227	167	Male	0	1.3615
474	227	167	Male	0	1.3764
475	228	167	Male	0	1.2196
476	228	167	Male	0	1.3408
477	1	170	Female	10	1.2230
478	1	170	Female	10	1.4579
479	1	170	Female	10	1.4630
480	1	170	Female	10	1.5087
481	1	170	Female	11	1.4819
482	1	170	Male	0	1.3451
483	1	170	Male	0	1.3519
484	1	170	Male	0	1.3664
485	1	170	Male	0	1.5423
486	4	170	Female	6	1.5216
487	4	170	Female	6	1.5417
488	4	170	Female	7	1.4841
489	4	170	Female	7	1.5607
490	4	170	Female	7	1.5835
491	4	170	Female	7	1.6092
492	4	170	Female	8	1.1724
493	4	170	Female	8	1.3057
494	4	170	Female	9	1.4539
495	4	170	Female	9	1.4763
496	4	170	Female	10	1.4340
497	4	170	Male	0	0.9527
498	4	170	Male	0	1.2268
499	4	170	Male	0	1.3312
500	4	170	Male	0	1.3754
501	4	170	Male	0	1.4718
502	4	170	Male	0	1.5220
503	4	170	Male	0	1.5360
504	4	170	Male	0	1.5529
505	4	170	Male	0	1.5538
506	4	170	Male	0	1.6026
507	5	170	Female	7	1.2989

508	5	170	Female	7	1.3455
509	5	170	Female	8	1.0892
510	5	170	Female	8	1.3636
511	5	170	Female	8	1.3712
512	5	170	Female	8	1.3808
513	5	170	Female	9	1.3788
514	5	170	Female	9	1.5128
515	5	170	Male	0	1.2814
516	5	170	Male	0	1.3024
517	7	170	Female	6	1.2953
518	7	170	Female	7	1.1436
519	7	170	Male	0	1.0754
520	8	170	Female	9	1.2727
521	8	170	Female	10	1.2097
522	8	170	Female	10	1.3022
523	8	170	Male	0	1.2344
524	8	170	Male	0	1.3623
525	11	170	Female	8	1.2706
526	11	170	Male	0	0.9775
527	11	170	Male	0	1.4523
528	13	170	Female	7	1.3126
529	13	170	Female	8	1.3477
530	13	170	Male	0	1.2289
531	13	170	Male	0	1.2289
532	14	170	Female	6	1.2519
533	14	170	Female	6	1.2538
534	14	170	Female	6	1.3167
535	14	170	Male	0	1.2589
536	14	170	Male	0	1.2763
537	15	170	Female	6	1.4236
538	15	170	Female	7	1.3397
539	15	170	Female	8	1.3424
540	15	170	Male	0	1.3623
541	15	170	Male	0	1.4078
542	16	170	Male	0	1.2777
543	16	170	Male	0	1.2786
544	17	170	Female	7	1.2787
545	17	170	Female	8	1.3296
546	18	170	Female	6	1.1867
547	18	170	Female	7	1.1659
548	18	170	Male	0	1.0969
549	18	170	Male	0	1.2196
550	18	170	Male	0	1.2348
551	21	170	Female	7	1.3276

552	21	170	Female	7	1.3546
553	21	170	Female	8	1.3451
554	21	170	Female	9	1.2742
555	21	170	Male	0	1.2352
556	21	170	Male	0	1.3028
557	21	170	Male	0	1.3809
558	22	170	Male	0	1.0462
559	22	170	Male	0	1.0788
560	22	170	Male	0	1.0817
561	22	170	Male	0	1.1112
562	22	170	Male	0	1.1185
563	22	170	Male	0	1.1277
564	22	170	Male	0	1.1343
565	22	170	Male	0	1.1474
566	22	170	Male	0	1.1481
567	22	170	Male	0	1.1529
568	22	170	Male	0	1.1561
569	22	170	Male	0	1.1700
570	22	170	Male	0	1.1765
571	22	170	Male	0	1.1847
572	22	170	Male	0	1.1858
573	22	170	Male	0	1.1865
574	22	170	Male	0	1.1902
575	22	170	Male	0	1.2034
576	22	170	Male	0	1.2682
577	22	170	Male	0	1.2774
578	22	170	Male	0	1.2846
579	22	170	Male	0	1.2847
580	22	170	Male	0	1.2862
581	22	170	Male	0	1.2884
582	22	170	Male	0	1.3070
583	22	170	Male	0	1.3170
584	22	170	Male	0	1.3197
585	22	170	Male	0	1.3198
586	22	170	Male	0	1.3404
587	22	170	Male	0	1.3443
588	22	170	Male	0	1.3506
589	22	170	Male	0	1.3680
590	22	170	Male	0	1.3767
591	22	170	Male	0	1.3833
592	24	170	Female	9	1.3910
593	24	170	Female	9	1.4529
594	24	170	Male	0	1.3619
595	24	170	Male	0	1.4296

596	26	170	Female	6	1.3586
597	26	170	Female	6	1.3781
598	26	170	Female	7	1.4479
599	26	170	Female	7	1.5398
600	26	170	Male	0	1.2667
601	26	170	Male	0	1.3657
602	26	170	Male	0	1.3678
603	26	170	Male	0	1.3692
604	26	170	Male	0	1.4033
605	27	170	Female	5	1.2903
606	27	170	Female	8	1.3152
607	29	170	Male	0	1.2493
608	29	170	Male	0	1.2905
609	29	170	Male	0	1.2971
610	29	170	Male	0	1.2972
611	29	170	Male	0	1.3154
612	29	170	Male	0	1.4231
613	31	170	Male	0	1.2931
614	31	170	Male	0	1.6484
615	34	170	Female	7	1.0708
616	36	170	Female	6	1.2796
617	36	170	Male	0	1.1373
618	36	170	Male	0	1.2562
619	36	170	Male	0	1.2585
620	37	170	Female	6	1.2288
621	37	170	Female	6	1.3678
622	37	170	Female	8	1.2857
623	37	170	Female	8	1.3158
624	37	170	Female	8	1.3341
625	37	170	Female	8	1.3496
626	38	170	Female	6	1.2367
627	38	170	Female	9	1.3667
628	38	170	Male	0	1.2057
629	38	170	Male	0	1.2258
630	38	170	Male	0	1.2464
631	42	170	Female	6	1.0703
632	42	170	Female	7	1.1376
633	42	170	Female	7	1.2791
634	42	170	Male	0	1.1304
635	42	170	Male	0	1.1813
636	43	170	Female	6	1.2640
637	43	170	Female	7	1.2644
638	43	170	Male	0	1.0259
639	48	170	Female	8	1.2759

640	48	170	Male	0	1.1804
641	53	170	Male	0	1.0855
642	54	170	Female	7	1.2752
643	55	170	Female	8	1.3232
644	56	170	Male	0	1.1334
645	56	170	Male	0	1.3106
646	56	170	Male	0	1.3117
647	56	170	Male	0	1.3311
648	57	170	Female	6	1.2487
649	57	170	Male	0	1.2419
650	57	170	Male	0	1.2748
651	59	170	Female	8	1.1266
652	60	170	Male	0	1.3954
653	60	170	Male	0	1.5775
654	60	170	Male	0	1.5907
655	60	170	Male	0	1.5993
656	60	170	Male	0	1.6109
657	60	170	Male	0	1.6267
658	60	170	Male	0	1.6272
659	60	170	Male	0	1.6980
660	60	170	Male	0	1.7407
661	60	170	Male	0	1.8393
662	60	170	Male	0	1.9238
663	61	170	Female	10	1.3489
664	61	170	Female	11	1.7553
665	61	170	Male	0	1.2380
666	63	170	Female	7	1.2267
667	63	170	Female	8	1.3302
668	64	170	Female	6	1.3903
669	64	170	Female	6	1.4233
670	64	170	Female	7	1.3817
671	64	170	Female	8	1.3328
672	64	170	Female	8	1.3376
673	64	170	Female	9	1.4155
674	64	170	Male	0	1.2442
675	64	170	Male	0	1.2483
676	64	170	Male	0	1.2483
677	64	170	Male	0	1.3687
678	64	170	Male	0	1.4071
679	65	170	Male	0	1.2351
680	65	170	Male	0	1.2649
681	66	170	Female	6	1.1119
682	66	170	Female	6	1.2544
683	66	170	Female	7	1.2121

684	66	170	Female	8	1.2491
685	66	170	Female	8	1.2493
686	66	170	Male	0	1.0065
687	66	170	Male	0	1.1829
688	66	170	Male	0	1.2049
689	66	170	Male	0	1.2242
690	66	170	Male	0	1.2772
691	66	170	Male	0	1.3047
692	67	170	Female	6	1.4596
693	67	170	Female	7	1.3881
694	67	170	Male	0	1.1882
695	69	170	Female	8	1.2581
696	69	170	Female	8	1.2859
697	69	170	Male	0	1.2146
698	70	170	Female	7	1.3576
699	70	170	Male	0	1.2371
700	70	170	Male	0	1.3166
701	70	170	Male	0	1.3341
702	70	170	Male	0	1.3489
703	71	170	Male	0	1.1187
704	73	170	Female	8	1.2372
705	73	170	Male	0	1.2057
706	73	170	Male	0	1.2711
707	73	170	Male	0	1.2986
708	74	170	Male	0	1.3336
709	74	170	Male	0	1.3632
710	74	170	Male	0	1.4549
711	74	170	Male	0	1.4994
712	75	170	Female	7	1.2201
713	75	170	Female	7	1.2553
714	75	170	Female	8	1.1838
715	75	170	Female	10	1.2493
716	75	170	Female	10	1.3346
717	75	170	Female	10	1.3547
718	75	170	Male	0	1.0921
719	75	170	Male	0	1.2296
720	75	170	Male	0	1.2497
721	75	170	Male	0	1.2539
722	75	170	Male	0	1.2778
723	75	170	Male	0	1.3018
724	75	170	Male	0	1.3213
725	75	170	Male	0	1.3624
726	75	170	Male	0	1.4431
727	76	170	Female	9	1.2571

728	76	170	Male	0	1.2051
729	78	170	Female	6	1.1097
730	78	170	Female	6	1.3743
731	78	170	Female	9	1.3976
732	78	170	Female	10	1.3196
733	78	170	Male	0	1.3582
734	78	170	Male	0	1.3848
735	78	170	Male	0	1.3911
736	81	170	Female	7	1.1432
737	81	170	Female	8	1.2277
738	81	170	Male	0	1.3173
739	81	170	Male	0	1.3221
740	81	170	Male	0	1.3304
741	86	170	Male	0	1.2009
742	86	170	Male	0	1.3027
743	87	170	Female	6	1.3826
744	87	170	Female	7	1.3427
745	87	170	Female	8	1.3377
746	87	170	Male	0	1.3609
747	87	170	Male	0	1.3712
748	87	170	Male	0	1.4139
749	87	170	Male	0	1.4197
750	87	170	Male	0	1.4233
751	88	170	Female	6	1.1987
752	88	170	Female	6	1.2425
753	88	170	Female	6	1.3871
754	88	170	Female	6	1.4061
755	88	170	Female	6	1.4130
756	88	170	Female	7	1.3137
757	88	170	Female	9	1.3436
758	88	170	Male	0	1.0116
759	88	170	Male	0	1.2046
760	88	170	Male	0	1.2587
761	88	170	Male	0	1.2766
762	88	170	Male	0	1.2870
763	88	170	Male	0	1.2899
764	88	170	Male	0	1.3201
765	88	170	Male	0	1.3743
766	90	170	Female	5	1.3392
767	90	170	Female	7	1.3819
768	90	170	Female	7	1.3866
769	90	170	Female	8	1.3928
770	90	170	Female	8	1.4043
771	90	170	Female	8	1.4369

772	90	170	Female	9	1.2996
773	90	170	Female	9	1.3988
774	90	170	Female	9	1.4379
775	90	170	Female	9	1.4476
776	90	170	Female	11	1.4084
777	90	170	Male	0	1.1432
778	90	170	Male	0	1.2238
779	90	170	Male	0	1.2879
780	90	170	Male	0	1.2921
781	90	170	Male	0	1.3578
782	90	170	Male	0	1.3759
783	90	170	Male	0	1.3787
784	90	170	Male	0	1.4137
785	90	170	Male	0	1.4310
786	90	170	Male	0	1.4917
787	91	170	Female	7	1.4366
788	91	170	Female	7	1.4799
789	91	170	Female	7	1.4904
790	91	170	Female	7	1.4929
791	91	170	Female	10	1.4915
792	91	170	Male	0	1.4216
793	96	170	Female	7	1.0318
794	96	170	Female	8	1.2399
795	96	170	Male	0	0.8577
796	97	170	Female	6	1.2687
797	97	170	Female	7	1.1961
798	97	170	Female	8	1.0914
799	97	170	Female	9	1.2296
800	97	170	Male	0	1.0697
801	97	170	Male	0	1.2143
802	97	170	Male	0	1.2239
803	97	170	Male	0	1.2756
804	97	170	Male	0	1.3344
805	98	170	Male	0	1.2576
806	101	170	Female	7	1.1255
807	101	170	Female	8	1.1463
808	101	170	Female	8	1.4186
809	101	170	Female	8	1.5371
810	101	170	Male	0	1.4104
811	101	170	Male	0	1.4353
812	101	170	Male	0	1.4391
813	101	170	Male	0	1.4567
814	104	170	Male	0	1.1774
815	104	170	Male	0	1.1781

816	104	170	Male	0	1.1786
817	105	170	Male	0	1.2123
818	106	170	Female	8	1.3165
819	106	170	Female	9	1.3930
820	106	170	Female	9	1.4367
821	106	170	Female	10	1.3231
822	106	170	Female	11	1.4178
823	106	170	Male	0	1.3269
824	107	170	Female	6	1.2004
825	200	170	Female	7	1.5989
826	200	170	Male	0	1.3668
827	200	170	Male	0	1.4266
828	200	170	Male	0	1.4343
829	200	170	Male	0	1.4552
830	200	170	Male	0	1.4555
831	200	170	Male	0	1.4597
832	200	170	Male	0	1.4668
833	200	170	Male	0	1.4713
834	200	170	Male	0	1.4727
835	200	170	Male	0	1.4772
836	200	170	Male	0	1.5354
837	200	170	Male	0	1.5394
838	200	170	Male	0	1.5601
839	200	170	Male	0	1.5647
840	201	170	Female	6	1.2605
841	201	170	Male	0	1.2317
842	201	170	Male	0	1.2850
843	201	170	Male	0	1.4070
844	202	170	Female	8	1.3059
845	204	170	Female	7	1.4671
846	204	170	Female	9	1.3811
847	204	170	Female	10	1.3338
848	204	170	Male	0	1.3257
849	204	170	Male	0	1.3586
850	204	170	Male	0	1.3731
851	204	170	Male	0	1.3743
852	204	170	Male	0	1.4231
853	204	170	Male	0	1.4351
854	204	170	Male	0	1.4425
855	204	170	Male	0	1.4498
856	204	170	Male	0	1.4584
857	204	170	Male	0	1.4657
858	204	170	Male	0	1.4876
859	204	170	Male	0	1.4907

860	204	170	Male	0	1.6029
861	205	170	Female	6	1.3351
862	205	170	Female	6	1.3933
863	205	170	Female	6	1.4298
864	205	170	Female	7	1.4238
865	205	170	Male	0	1.0419
866	205	170	Male	0	1.3166
867	205	170	Male	0	1.3936
868	205	170	Male	0	1.4006
869	205	170	Male	0	1.4173
870	205	170	Male	0	1.4712
871	205	170	Male	0	1.4876
872	206	170	Female	8	1.3474
873	206	170	Female	8	1.5031
874	206	170	Male	0	1.3992
875	206	170	Male	0	1.4799
876	206	170	Male	0	1.5091
877	206	170	Male	0	1.5120
878	206	170	Male	0	1.5211
879	206	170	Male	0	1.5842
880	207	170	Female	4	1.3242
881	207	170	Female	5	1.2469
882	207	170	Female	7	1.4643
883	207	170	Female	8	1.2969
884	207	170	Female	9	1.2566
885	207	170	Female	10	1.4219
886	207	170	Male	0	1.3763
887	207	170	Male	0	1.4956
888	207	170	Male	0	1.5177
889	207	170	Male	0	1.5262
890	207	170	Male	0	1.5461
891	207	170	Male	0	1.5482
892	208	170	Male	0	1.4650
893	210	170	Female	6	1.3573
894	210	170	Male	0	1.2832
895	210	170	Male	0	1.3079
896	210	170	Male	0	1.3481
897	210	170	Male	0	1.3484
898	210	170	Male	0	1.3616
899	210	170	Male	0	1.3684
900	210	170	Male	0	1.3786
901	210	170	Male	0	1.4127
902	210	170	Male	0	1.4236
903	210	170	Male	0	1.4364

904	211	170	Female	6	1.2934
905	211	170	Female	6	1.4214
906	211	170	Male	0	1.3009
907	211	170	Male	0	1.3136
908	211	170	Male	0	1.3630
909	211	170	Male	0	1.3977
910	211	170	Male	0	1.4108
911	211	170	Male	0	1.4495
912	211	170	Male	0	1.4593
913	212	170	Female	6	1.2800
914	212	170	Female	7	1.3322
915	212	170	Female	8	1.3351
916	212	170	Female	8	1.3925
917	212	170	Male	0	1.2558
918	212	170	Male	0	1.3225
919	213	170	Female	4	1.4371
920	213	170	Female	6	1.2821
921	213	170	Female	6	1.3725
922	213	170	Female	6	1.4057
923	213	170	Female	7	1.3116
924	213	170	Female	7	1.3867
925	213	170	Male	0	1.3158
926	213	170	Male	0	1.3438
927	213	170	Male	0	1.3987
928	213	170	Male	0	1.4379
929	213	170	Male	0	1.4509
930	213	170	Male	0	1.4609
931	215	170	Female	7	1.4726
932	215	170	Female	8	1.4627
933	215	170	Female	8	1.4637
934	215	170	Female	8	1.5139
935	215	170	Female	8	1.5861
936	215	170	Female	9	1.4691
937	215	170	Female	10	1.3805
938	215	170	Male	0	1.5239
939	217	170	Female	6	1.1043
940	217	170	Male	0	1.0422
941	217	170	Male	0	1.3218
942	217	170	Male	0	1.3597
943	217	170	Male	0	1.3684
944	217	170	Male	0	1.3717
945	217	170	Male	0	1.4164
946	217	170	Male	0	1.4613
947	218	170	Female	6	1.2081

948	218	170	Female	7	1.4788
949	218	170	Male	0	1.1236
950	218	170	Male	0	1.3159
951	218	170	Male	0	1.3204
952	218	170	Male	0	1.3697
953	218	170	Male	0	1.3722
954	218	170	Male	0	1.3998
955	218	170	Male	0	1.4084
956	219	170	Female	5	1.4400
957	219	170	Female	6	1.5558
958	219	170	Male	0	1.1277
959	219	170	Male	0	1.1551
960	219	170	Male	0	1.1730
961	219	170	Male	0	1.2356
962	219	170	Male	0	1.2731
963	219	170	Male	0	1.2759
964	219	170	Male	0	1.3353
965	220	170	Female	6	1.2568
966	220	170	Female	8	1.3311
967	220	170	Male	0	1.1267
968	220	170	Male	0	1.2191
969	220	170	Male	0	1.2543
970	220	170	Male	0	1.3042
971	220	170	Male	0	1.3099
972	220	170	Male	0	1.3122
973	220	170	Male	0	1.3232
974	220	170	Male	0	1.3393
975	222	170	Female	6	1.4134
976	222	170	Female	7	1.1708
977	222	170	Female	7	1.2599
978	222	170	Female	7	1.3823
979	222	170	Female	7	1.5624
980	222	170	Male	0	1.1444
981	222	170	Male	0	1.2320
982	222	170	Male	0	1.2976
983	222	170	Male	0	1.3132
984	222	170	Male	0	1.3878
985	223	170	Female	5	1.3205
986	223	170	Female	5	1.4510
987	223	170	Female	6	1.1404
988	223	170	Female	6	1.5171
989	223	170	Female	6	1.5834
990	223	170	Female	7	1.3440
991	223	170	Female	7	1.4705

992	223	170	Female	7	1.5382
993	223	170	Female	9	1.2517
994	223	170	Male	0	1.1550
995	223	170	Male	0	1.1621
996	223	170	Male	0	1.1890
997	223	170	Male	0	1.2310
998	223	170	Male	0	1.3031
999	223	170	Male	0	1.3098
1000	224	170	Female	6	1.1599
1001	224	170	Female	6	1.3170
1002	224	170	Male	0	1.2148
1003	224	170	Male	0	1.2194
1004	224	170	Male	0	1.2208
1005	224	170	Male	0	1.2555
1006	224	170	Male	0	1.2634
1007	224	170	Male	0	1.2965
1008	224	170	Male	0	1.3317
1009	226	170	Female	6	1.2814
1010	226	170	Female	6	1.4324
1011	226	170	Female	7	1.1743
1012	226	170	Female	8	1.1799
1013	226	170	Female	8	1.2200
1014	226	170	Female	9	1.4563
1015	226	170	Female	10	1.1481
1016	226	170	Male	0	1.1600
1017	226	170	Male	0	1.1781
1018	226	170	Male	0	1.1791
1019	226	170	Male	0	1.2780
1020	226	170	Male	0	1.3032
1021	226	170	Male	0	1.3248
1022	226	170	Male	0	1.3493
1023	226	170	Male	0	1.4106
1024	227	170	Female	5	1.0911
1025	227	170	Female	6	1.0185
1026	227	170	Female	7	1.1477
1027	227	170	Female	7	1.2736
1028	227	170	Female	9	1.0648
1029	227	170	Male	0	1.1937
1030	227	170	Male	0	1.2550
1031	227	170	Male	0	1.3790
1032	227	170	Male	0	1.3896
1033	227	170	Male	0	1.3929
1034	228	170	Male	0	1.2314
1035	228	170	Male	0	1.2348

1036	228	170	Male	0	1.2697
1037	228	170	Male	0	1.3036
1038	228	170	Male	0	1.3138
1039	228	170	Male	0	1.3741
1040	228	170	Male	0	1.3765
1041	228	170	Male	0	1.4198
1042	1	172	Female	7	1.3213
1043	1	172	Female	8	1.3978
1044	1	172	Female	10	1.3621
1045	1	172	Female	10	1.4911
1046	1	172	Male	0	1.3167
1047	1	172	Male	0	1.3182
1048	1	172	Male	0	1.3188
1049	1	172	Male	0	1.3319
1050	1	172	Male	0	1.3635
1051	1	172	Male	0	1.4247
1052	1	172	Male	0	1.4356
1053	1	172	Male	0	1.4657
1054	4	172	Female	5	1.2294
1055	4	172	Female	6	1.2739
1056	4	172	Female	6	1.3935
1057	4	172	Female	6	1.4997
1058	4	172	Female	7	1.4898
1059	4	172	Female	7	1.4934
1060	4	172	Female	9	1.4683
1061	4	172	Male	0	1.4053
1062	4	172	Male	0	1.4674
1063	5	172	Female	8	1.2726
1064	7	172	Female	6	1.1847
1065	7	172	Female	6	1.1936
1066	7	172	Female	7	1.2138
1067	8	172	Female	8	1.2623
1068	8	172	Male	0	1.2977
1069	11	172	Female	6	1.1184
1070	11	172	Female	7	1.2627
1071	11	172	Female	7	1.3293
1072	11	172	Male	0	0.8296
1073	11	172	Male	0	1.1812
1074	11	172	Male	0	1.2421
1075	11	172	Male	0	1.3324
1076	11	172	Male	0	1.3641
1077	13	172	Female	7	1.3056
1078	13	172	Female	7	1.3761
1079	13	172	Female	8	1.2703

1080	13	172	Female	8	1.3507
1081	13	172	Female	8	1.4223
1082	13	172	Female	9	1.3623
1083	13	172	Male	0	1.3180
1084	14	172	Male	0	0.9012
1085	14	172	Male	0	1.2040
1086	14	172	Male	0	1.2052
1087	14	172	Male	0	1.2369
1088	14	172	Male	0	1.2416
1089	14	172	Male	0	1.3989
1090	14	172	Male	0	1.4299
1091	15	172	Male	0	1.3167
1092	15	172	Male	0	1.3256
1093	15	172	Male	0	1.3563
1094	15	172	Male	0	1.3942
1095	15	172	Male	0	1.4167
1096	15	172	Male	0	1.4197
1097	15	172	Male	0	1.4587
1098	16	172	Male	0	1.2215
1099	18	172	Female	6	1.2604
1100	19	172	Male	0	1.3784
1101	20	172	Male	0	1.2798
1102	21	172	Female	7	1.2617
1103	21	172	Male	0	1.2985
1104	21	172	Male	0	1.3268
1105	21	172	Male	0	1.3738
1106	22	172	Female	10	1.3102
1107	22	172	Female	10	1.3344
1108	22	172	Male	0	1.0441
1109	22	172	Male	0	1.0743
1110	22	172	Male	0	1.2853
1111	24	172	Female	7	1.2959
1112	24	172	Female	9	1.3891
1113	24	172	Female	9	1.4529
1114	24	172	Male	0	1.2895
1115	26	172	Female	7	1.3738
1116	26	172	Female	9	1.4592
1117	26	172	Male	0	1.3821
1118	26	172	Male	0	1.3833
1119	26	172	Male	0	1.4457
1120	26	172	Male	0	1.4747
1121	29	172	Female	7	1.4496
1122	29	172	Female	8	1.2985
1123	29	172	Female	8	1.3513

1124	29	172	Male	0	1.3367
1125	31	172	Female	8	1.2738
1126	31	172	Female	8	1.4624
1127	31	172	Male	0	1.2645
1128	31	172	Male	0	1.2871
1129	31	172	Male	0	1.4034
1130	35	172	Male	0	1.0287
1131	35	172	Male	0	1.3693
1132	35	172	Male	0	1.3963
1133	35	172	Male	0	1.4811
1134	36	172	Female	7	1.2544
1135	36	172	Female	8	1.1978
1136	36	172	Male	0	1.1865
1137	36	172	Male	0	1.2406
1138	36	172	Male	0	1.3284
1139	36	172	Male	0	1.3776
1140	37	172	Female	8	1.2772
1141	37	172	Female	9	1.3771
1142	37	172	Male	0	1.3522
1143	37	172	Male	0	1.4207
1144	38	172	Female	6	1.3635
1145	38	172	Female	7	1.1989
1146	38	172	Female	7	1.2936
1147	38	172	Male	0	1.0737
1148	38	172	Male	0	1.1068
1149	38	172	Male	0	1.2024
1150	38	172	Male	0	1.2728
1151	38	172	Male	0	1.3247
1152	42	172	Male	0	0.8503
1153	42	172	Male	0	1.2732
1154	43	172	Male	0	1.3531
1155	43	172	Male	0	1.3670
1156	46	172	Female	6	1.2297
1157	48	172	Male	0	1.3154
1158	50	172	Female	6	1.3407
1159	51	172	Female	7	1.1673
1160	51	172	Male	0	1.2619
1161	54	172	Female	9	1.1296
1162	55	172	Female	7	1.2389
1163	55	172	Male	0	1.2803
1164	56	172	Female	9	1.0402
1165	56	172	Male	0	1.2279
1166	56	172	Male	0	1.2791
1167	56	172	Male	0	1.3244

1168	57	172	Female	7	1.2382
1169	57	172	Female	7	1.3706
1170	57	172	Female	8	1.1296
1171	57	172	Male	0	1.2454
1172	57	172	Male	0	1.3124
1173	57	172	Male	0	1.3307
1174	60	172	Female	10	1.5884
1175	60	172	Female	10	1.8379
1176	60	172	Male	0	1.5978
1177	60	172	Male	0	1.6159
1178	60	172	Male	0	1.6564
1179	60	172	Male	0	1.7671
1180	60	172	Male	0	1.8503
1181	60	172	Male	0	2.0117
1182	61	172	Female	10	1.3428
1183	61	172	Male	0	1.2380
1184	62	172	Female	7	1.2247
1185	62	172	Female	7	1.2644
1186	63	172	Female	7	1.3331
1187	63	172	Male	0	1.3158
1188	63	172	Male	0	1.3612
1189	64	172	Female	6	1.3914
1190	64	172	Female	7	1.3692
1191	64	172	Male	0	1.3418
1192	64	172	Male	0	1.3657
1193	64	172	Male	0	1.3879
1194	64	172	Male	0	1.4275
1195	65	172	Female	6	1.2316
1196	65	172	Female	8	1.3553
1197	66	172	Male	0	1.1821
1198	66	172	Male	0	1.2447
1199	67	172	Female	7	1.3893
1200	67	172	Female	9	1.3043
1201	67	172	Female	9	1.3146
1202	67	172	Female	9	1.3881
1203	67	172	Male	0	1.2859
1204	67	172	Male	0	1.2978
1205	67	172	Male	0	1.3319
1206	67	172	Male	0	1.3368
1207	69	172	Female	7	1.2884
1208	69	172	Female	7	1.2953
1209	69	172	Male	0	1.1203
1210	69	172	Male	0	1.3275
1211	70	172	Female	6	1.2971

1212	70	172	Female	9	1.3313
1213	71	172	Male	0	1.1137
1214	71	172	Male	0	1.2544
1215	71	172	Male	0	1.2603
1216	73	172	Female	8	1.2699
1217	74	172	Female	8	1.3516
1218	74	172	Female	8	1.4591
1219	74	172	Male	0	1.2286
1220	74	172	Male	0	1.3269
1221	75	172	Female	7	1.2097
1222	75	172	Male	0	1.2466
1223	76	172	Female	6	1.0137
1224	76	172	Female	7	1.2096
1225	76	172	Male	0	1.2642
1226	77	172	Female	7	1.1880
1227	77	172	Female	7	1.2241
1228	77	172	Male	0	1.1867
1229	77	172	Male	0	1.2197
1230	78	172	Female	6	1.0859
1231	78	172	Male	0	1.3518
1232	81	172	Female	9	1.3474
1233	81	172	Male	0	1.2937
1234	81	172	Male	0	1.2957
1235	81	172	Male	0	1.3136
1236	81	172	Male	0	1.3173
1237	81	172	Male	0	1.3221
1238	83	172	Male	0	1.2374
1239	86	172	Male	0	1.2443
1240	86	172	Male	0	1.3344
1241	87	172	Female	6	1.3564
1242	87	172	Female	7	1.4666
1243	87	172	Male	0	1.3738
1244	87	172	Male	0	1.4432
1245	88	172	Female	7	1.0414
1246	88	172	Female	7	1.4122
1247	88	172	Female	8	1.3776
1248	88	172	Female	9	1.4303
1249	88	172	Male	0	0.9769
1250	88	172	Male	0	1.4121
1251	90	172	Female	6	1.2389
1252	90	172	Female	6	1.4123
1253	90	172	Female	7	1.3952
1254	90	172	Female	8	1.4436
1255	90	172	Female	9	1.3798

1256	90	172	Female	9	1.3971
1257	90	172	Male	0	1.1994
1258	90	172	Male	0	1.2011
1259	90	172	Male	0	1.3937
1260	90	172	Male	0	1.4049
1261	90	172	Male	0	1.4467
1262	90	172	Male	0	1.4741
1263	91	172	Female	7	1.2712
1264	91	172	Female	8	1.5870
1265	92	172	Male	0	1.2353
1266	92	172	Male	0	1.2797
1267	92	172	Male	0	1.3099
1268	92	172	Male	0	1.5435
1269	96	172	Male	0	1.2012
1270	97	172	Female	7	1.2622
1271	97	172	Female	9	1.2382
1272	97	172	Female	10	1.2053
1273	97	172	Male	0	1.2418
1274	98	172	Female	6	1.0028
1275	99	172	Female	6	1.2321
1276	101	172	Female	7	1.3137
1277	101	172	Female	10	1.3926
1278	101	172	Male	0	1.4947
1279	101	172	Male	0	1.5014
1280	102	172	Female	6	1.5780
1281	102	172	Female	9	1.5172
1282	102	172	Female	10	1.4397
1283	102	172	Female	10	1.4413
1284	102	172	Male	0	1.5174
1285	103	172	Male	0	1.0347
1286	104	172	Female	6	1.2227
1287	104	172	Male	0	1.1723
1288	105	172	Female	9	1.3688
1289	106	172	Female	8	1.3261
1290	106	172	Male	0	1.4069
1291	107	172	Female	7	1.2613
1292	107	172	Male	0	1.2816
1293	200	172	Female	6	1.5161
1294	200	172	Female	8	1.4435
1295	200	172	Male	0	1.2562
1296	200	172	Male	0	1.3309
1297	200	172	Male	0	1.4523
1298	200	172	Male	0	1.5642
1299	200	172	Male	0	1.5877

1300	201	172	Female	8	1.2051
1301	204	172	Male	0	1.0351
1302	204	172	Male	0	1.3582
1303	204	172	Male	0	1.3591
1304	204	172	Male	0	1.3922
1305	204	172	Male	0	1.3924
1306	204	172	Male	0	1.4086
1307	204	172	Male	0	1.5418
1308	205	172	Male	0	1.3356
1309	205	172	Male	0	1.4186
1310	206	172	Female	6	1.3459
1311	206	172	Female	6	1.3459
1312	206	172	Female	6	1.4044
1313	206	172	Female	7	1.4857
1314	206	172	Male	0	1.4339
1315	206	172	Male	0	1.5487
1316	207	172	Female	6	1.4778
1317	207	172	Female	7	1.2556
1318	207	172	Female	7	1.3237
1319	207	172	Female	7	1.4227
1320	207	172	Female	7	1.4876
1321	207	172	Female	8	1.4702
1322	207	172	Male	0	1.3676
1323	207	172	Male	0	1.3900
1324	207	172	Male	0	1.4255
1325	207	172	Male	0	1.4450
1326	207	172	Male	0	1.4828
1327	207	172	Male	0	1.4875
1328	210	172	Female	6	1.2131
1329	210	172	Female	6	1.4146
1330	210	172	Female	8	1.3408
1331	210	172	Male	0	1.2917
1332	210	172	Male	0	1.3627
1333	210	172	Male	0	1.3898
1334	211	172	Female	6	1.4225
1335	211	172	Female	7	1.2398
1336	211	172	Male	0	1.3141
1337	212	172	Female	6	1.2539
1338	212	172	Female	8	1.3014
1339	212	172	Male	0	1.2673
1340	212	172	Male	0	1.3984
1341	212	172	Male	0	1.4465
1342	213	172	Female	6	1.3301
1343	213	172	Female	7	1.3596

1344	213	172	Male	0	1.0144
1345	213	172	Male	0	1.1143
1346	213	172	Male	0	1.4572
1347	215	172	Female	6	1.3981
1348	215	172	Female	6	1.4018
1349	215	172	Female	6	1.4457
1350	215	172	Female	7	1.5142
1351	215	172	Female	8	1.3677
1352	215	172	Female	10	1.4036
1353	215	172	Male	0	1.3572
1354	215	172	Male	0	1.4262
1355	215	172	Male	0	1.4591
1356	215	172	Male	0	1.4826
1357	215	172	Male	0	1.5961
1358	217	172	Female	8	1.3247
1359	217	172	Female	9	1.3708
1360	217	172	Female	10	1.2981
1361	217	172	Female	10	1.3443
1362	217	172	Male	0	1.2783
1363	217	172	Male	0	1.3679
1364	217	172	Male	0	1.4787
1365	218	172	Female	6	1.3907
1366	218	172	Female	7	1.3928
1367	218	172	Female	8	1.3628
1368	218	172	Male	0	1.3064
1369	218	172	Male	0	1.3131
1370	218	172	Male	0	1.3396
1371	218	172	Male	0	1.3695
1372	218	172	Male	0	1.3749
1373	218	172	Male	0	1.3801
1374	220	172	Male	0	1.2814
1375	220	172	Male	0	1.3132
1376	222	172	Female	6	1.2219
1377	222	172	Female	6	1.3873
1378	222	172	Female	6	1.4114
1379	222	172	Male	0	1.1919
1380	222	172	Male	0	1.2525
1381	222	172	Male	0	1.2964
1382	222	172	Male	0	1.3777
1383	223	172	Female	5	1.3838
1384	223	172	Female	5	1.4951
1385	223	172	Female	6	1.2957
1386	223	172	Female	6	1.4672
1387	223	172	Female	8	1.1572

1388	223	172	Female	8	1.2517
1389	223	172	Male	0	1.1686
1390	223	172	Male	0	1.1764
1391	223	172	Male	0	1.2328
1392	223	172	Male	0	1.2373
1393	223	172	Male	0	1.3424
1394	224	172	Male	0	1.2342
1395	224	172	Male	0	1.2567
1396	224	172	Male	0	1.2705
1397	224	172	Male	0	1.3043
1398	224	172	Male	0	1.3180
1399	225	172	Female	6	1.3786
1400	225	172	Female	9	1.3894
1401	226	172	Female	7	1.5058
1402	226	172	Female	9	1.0718
1403	227	172	Female	6	1.1705
1404	227	172	Male	0	1.3087
1405	227	172	Male	0	1.3815
1406	227	172	Male	0	1.3935
1407	227	172	Male	0	1.4175
1408	228	172	Female	6	1.0776
1409	228	172	Female	6	1.4373
1410	228	172	Female	8	1.1160
1411	228	172	Male	0	1.1598
1412	228	172	Male	0	1.2447
1413	228	172	Male	0	1.2533
1414	228	172	Male	0	1.2595
1415	228	172	Male	0	1.3329
1416	228	172	Male	0	1.3545
1417	228	172	Male	0	1.3679
1418	228	172	Male	0	1.3711
1419	1	174	Female	9	1.3784
1420	1	174	Female	9	1.4226
1421	1	174	Female	10	1.3021
1422	1	174	Female	10	1.3100
1423	1	174	Male	0	1.4803
1424	3	174	Female	7	1.1521
1425	4	174	Female	8	1.5107
1426	4	174	Male	0	1.0628
1427	4	174	Male	0	1.4641
1428	4	174	Male	0	1.5569
1429	5	174	Female	7	1.2347
1430	5	174	Male	0	1.2603
1431	8	174	Female	9	1.2486

1432	11	174	Female	7	1.4172
1433	11	174	Female	8	1.2394
1434	11	174	Female	8	1.4105
1435	11	174	Male	0	1.1530
1436	11	174	Male	0	1.3914
1437	13	174	Female	9	1.2845
1438	13	174	Female	9	1.3536
1439	14	174	Male	0	1.1053
1440	14	174	Male	0	1.4262
1441	15	174	Female	8	1.2507
1442	16	174	Female	6	1.2658
1443	17	174	Female	8	1.3144
1444	18	174	Female	6	1.1308
1445	18	174	Male	0	1.1497
1446	18	174	Male	0	1.1497
1447	19	174	Female	8	1.3853
1448	19	174	Male	0	1.4110
1449	21	174	Female	9	1.3254
1450	22	174	Female	8	1.4096
1451	22	174	Male	0	1.0302
1452	24	174	Female	8	1.3518
1453	24	174	Male	0	1.3049
1454	24	174	Male	0	1.3382
1455	26	174	Female	7	1.3798
1456	26	174	Female	9	1.0582
1457	26	174	Female	9	1.3144
1458	26	174	Male	0	1.2902
1459	26	174	Male	0	1.4485
1460	26	174	Male	0	1.5278
1461	27	174	Female	8	1.2453
1462	27	174	Female	10	1.2864
1463	29	174	Female	7	1.3598
1464	31	174	Female	7	1.3842
1465	31	174	Female	8	1.3527
1466	31	174	Female	9	1.3534
1467	35	174	Female	6	1.3702
1468	35	174	Female	9	1.2654
1469	35	174	Female	9	1.3365
1470	35	174	Female	9	1.3875
1471	35	174	Male	0	1.2319
1472	36	174	Female	7	1.2128
1473	36	174	Male	0	1.0329
1474	38	174	Female	7	1.2537
1475	38	174	Female	7	1.3392

1476	38	174	Male	0	1.2417
1477	38	174	Male	0	1.2681
1478	46	174	Female	6	1.1441
1479	46	174	Male	0	1.1593
1480	46	174	Male	0	1.1681
1481	46	174	Male	0	1.1991
1482	46	174	Male	0	1.2059
1483	48	174	Male	0	1.3069
1484	50	174	Female	6	1.2223
1485	50	174	Female	6	1.2963
1486	50	174	Female	6	1.3289
1487	50	174	Female	6	1.3337
1488	53	174	Female	6	1.1910
1489	53	174	Female	6	1.2275
1490	53	174	Female	7	1.2458
1491	53	174	Female	8	1.1477
1492	53	174	Female	9	1.2078
1493	53	174	Female	9	1.2729
1494	53	174	Male	0	1.2160
1495	56	174	Female	10	1.1941
1496	57	174	Female	9	1.2708
1497	57	174	Male	0	1.1846
1498	57	174	Male	0	1.3385
1499	60	174	Female	8	1.6849
1500	60	174	Female	9	1.6764
1501	60	174	Female	10	1.7561
1502	60	174	Female	10	2.1895
1503	60	174	Female	10	2.2581
1504	60	174	Male	0	1.4742
1505	60	174	Male	0	1.7746
1506	60	174	Male	0	1.7839
1507	62	174	Male	0	1.1962
1508	64	174	Female	6	1.4413
1509	64	174	Female	7	1.3853
1510	64	174	Female	7	1.4338
1511	64	174	Female	10	1.4538
1512	65	174	Female	6	1.2684
1513	65	174	Male	0	1.2133
1514	65	174	Male	0	1.2192
1515	66	174	Male	0	1.1161
1516	66	174	Male	0	1.2614
1517	66	174	Male	0	1.2619
1518	67	174	Female	6	1.2992
1519	67	174	Female	6	1.3226

1520	67	174	Female	8	1.3252
1521	67	174	Female	8	1.3592
1522	67	174	Male	0	1.2891
1523	67	174	Male	0	1.3657
1524	67	174	Male	0	1.3977
1525	69	174	Female	9	1.2973
1526	69	174	Female	10	1.2536
1527	69	174	Male	0	1.2498
1528	69	174	Male	0	1.2641
1529	70	174	Male	0	1.3253
1530	74	174	Female	8	1.3296
1531	74	174	Female	9	1.3286
1532	74	174	Female	9	1.3662
1533	74	174	Male	0	1.1939
1534	74	174	Male	0	1.3127
1535	74	174	Male	0	1.4336
1536	75	174	Male	0	1.2652
1537	77	174	Male	0	1.2053
1538	77	174	Male	0	1.2323
1539	78	174	Female	6	1.2461
1540	78	174	Female	6	1.3642
1541	78	174	Female	9	1.3341
1542	78	174	Male	0	1.3656
1543	81	174	Male	0	1.2364
1544	83	174	Male	0	1.2105
1545	84	174	Male	0	1.2082
1546	86	174	Male	0	0.9511
1547	86	174	Male	0	1.2247
1548	87	174	Female	6	1.3756
1549	87	174	Female	8	1.4042
1550	87	174	Female	8	1.4372
1551	87	174	Male	0	1.3144
1552	87	174	Male	0	1.3986
1553	88	174	Male	0	1.2268
1554	90	174	Male	0	1.3727
1555	91	174	Female	7	1.4372
1556	91	174	Female	7	1.4766
1557	91	174	Female	8	1.2339
1558	91	174	Male	0	1.3919
1559	96	174	Female	6	1.2257
1560	96	174	Female	8	1.2678
1561	96	174	Male	0	1.2491
1562	97	174	Female	8	1.2921
1563	97	174	Male	0	1.1589

1564	97	174	Male	0	1.2326
1565	101	174	Female	7	1.0654
1566	102	174	Female	7	1.4366
1567	102	174	Female	7	1.4937
1568	102	174	Female	9	1.4347
1569	102	174	Male	0	1.4694
1570	102	174	Male	0	1.5182
1571	106	174	Female	9	1.4816
1572	106	174	Male	0	1.3352
1573	107	174	Female	8	1.2712
1574	107	174	Female	9	1.1754
1575	107	174	Female	9	1.2459
1576	107	174	Male	0	1.3011
1577	200	174	Female	6	1.4140
1578	200	174	Female	7	1.4132
1579	200	174	Male	0	1.4211
1580	201	174	Male	0	1.2557
1581	202	174	Male	0	1.1726
1582	204	174	Male	0	1.2432
1583	204	174	Male	0	1.3521
1584	206	174	Female	6	1.3981
1585	207	174	Female	9	1.2798
1586	208	174	Female	8	1.2511
1587	212	174	Female	8	1.4709
1588	212	174	Male	0	1.2978
1589	215	174	Female	7	1.4783
1590	217	174	Female	7	1.2152
1591	217	174	Female	10	1.4584
1592	220	174	Female	6	1.2631
1593	220	174	Female	6	1.2641
1594	222	174	Male	0	1.3183
1595	224	174	Female	7	1.1554
1596	224	174	Female	7	1.5190
1597	224	174	Female	8	1.2431
1598	224	174	Female	8	1.2746
1599	224	174	Female	8	1.4930
1600	224	174	Male	0	1.1645
1601	224	174	Male	0	1.3776
1602	226	174	Female	7	1.3266
1603	226	174	Female	7	1.4987
1604	227	174	Female	6	1.0167
1605	227	174	Female	6	1.0476
1606	227	174	Female	6	1.3729
1607	227	174	Female	7	1.5175

1608	227	174	Female	10	1.1012
1609	4	177	Female	6	1.5090
1610	4	177	Female	7	1.4627
1611	4	177	Female	7	1.5849
1612	4	177	Female	7	1.6207
1613	4	177	Female	9	1.4448
1614	4	177	Male	0	1.2140
1615	4	177	Male	0	1.2303
1616	4	177	Male	0	1.4246
1617	4	177	Male	0	1.4993
1618	7	177	Female	6	1.3256
1619	7	177	Female	7	1.1928
1620	7	177	Female	7	1.3174
1621	7	177	Female	8	1.2269
1622	7	177	Female	8	1.2528
1623	7	177	Male	0	0.9024
1624	7	177	Male	0	1.1199
1625	7	177	Male	0	1.1983
1626	7	177	Male	0	1.2521
1627	7	177	Male	0	1.2626
1628	7	177	Male	0	1.2979
1629	13	177	Female	8	1.4007
1630	13	177	Female	9	1.2486
1631	13	177	Female	9	1.3172
1632	13	177	Female	9	1.4128
1633	13	177	Male	0	0.9599
1634	13	177	Male	0	1.0697
1635	13	177	Male	0	1.1143
1636	13	177	Male	0	1.3228
1637	18	177	Female	6	1.3034
1638	18	177	Female	7	1.2478
1639	18	177	Female	7	1.3134
1640	18	177	Male	0	1.0993
1641	18	177	Male	0	1.1968
1642	18	177	Male	0	1.3190
1643	20	177	Female	6	1.2033
1644	20	177	Female	7	1.2804
1645	20	177	Female	8	1.3167
1646	20	177	Female	9	1.3071
1647	20	177	Female	9	1.3116
1648	20	177	Male	0	1.2397
1649	20	177	Male	0	1.2551
1650	20	177	Male	0	1.2734
1651	20	177	Male	0	1.9986

1652	24	177	Female	6	1.2954
1653	24	177	Female	6	1.2985
1654	24	177	Female	6	1.3811
1655	24	177	Female	7	1.3328
1656	24	177	Female	7	1.3611
1657	24	177	Female	7	1.3841
1658	24	177	Female	8	1.2359
1659	24	177	Female	8	1.3499
1660	24	177	Female	8	1.3633
1661	24	177	Male	0	1.2304
1662	24	177	Male	0	1.3173
1663	26	177	Female	6	1.4647
1664	26	177	Female	6	1.4734
1665	26	177	Female	8	1.3483
1666	26	177	Male	0	1.3019
1667	26	177	Male	0	1.3128
1668	26	177	Male	0	1.3323
1669	26	177	Male	0	1.3810
1670	26	177	Male	0	1.3849
1671	26	177	Male	0	1.4024
1672	26	177	Male	0	1.4659
1673	27	177	Female	7	1.3240
1674	27	177	Female	10	1.2239
1675	27	177	Male	0	1.1895
1676	27	177	Male	0	1.2191
1677	27	177	Male	0	1.2669
1678	27	177	Male	0	1.3076
1679	29	177	Male	0	1.3064
1680	29	177	Male	0	1.3638
1681	29	177	Male	0	1.3862
1682	31	177	Female	6	1.2486
1683	31	177	Female	6	1.2578
1684	31	177	Female	7	1.3156
1685	31	177	Female	7	1.3739
1686	31	177	Female	8	1.3099
1687	31	177	Male	0	1.2094
1688	31	177	Male	0	1.2742
1689	31	177	Male	0	1.2808
1690	31	177	Male	0	1.2878
1691	36	177	Female	6	0.9805
1692	36	177	Female	6	1.0584
1693	36	177	Female	6	1.1628
1694	36	177	Female	7	1.1527
1695	36	177	Female	7	1.2671

1696	36	177	Female	8	1.2433
1697	36	177	Female	10	1.2391
1698	36	177	Male	0	1.0981
1699	36	177	Male	0	1.2360
1700	36	177	Male	0	1.2887
1701	37	177	Female	8	1.2319
1702	37	177	Female	8	1.3518
1703	37	177	Male	0	1.2444
1704	43	177	Female	7	1.3346
1705	43	177	Female	7	1.3522
1706	46	177	Male	0	1.1358
1707	46	177	Male	0	1.2121
1708	50	177	Female	6	1.2693
1709	50	177	Female	8	1.3139
1710	50	177	Female	9	1.2799
1711	50	177	Male	0	1.2769
1712	50	177	Male	0	1.2941
1713	53	177	Female	7	1.2540
1714	53	177	Male	0	1.1809
1715	53	177	Male	0	1.2314
1716	56	177	Female	8	1.3130
1717	56	177	Male	0	1.2033
1718	57	177	Female	6	1.2681
1719	57	177	Female	6	1.3922
1720	57	177	Female	8	1.2622
1721	57	177	Female	8	1.2664
1722	57	177	Female	8	1.2944
1723	57	177	Female	8	1.2949
1724	57	177	Female	10	1.2492
1725	60	177	Female	8	1.4847
1726	60	177	Female	8	1.7499
1727	60	177	Female	8	1.8040
1728	60	177	Female	8	2.1431
1729	60	177	Female	9	1.6116
1730	60	177	Female	9	1.6553
1731	60	177	Female	9	1.6741
1732	60	177	Female	9	1.6913
1733	60	177	Female	9	1.7021
1734	60	177	Female	9	1.7914
1735	60	177	Female	10	1.2862
1736	60	177	Female	10	1.8547
1737	60	177	Male	0	1.3537
1738	60	177	Male	0	1.5706
1739	60	177	Male	0	1.5922

1740	60	177	Male	0	1.5967
1741	60	177	Male	0	1.6027
1742	60	177	Male	0	1.6888
1743	60	177	Male	0	1.6903
1744	60	177	Male	0	1.7109
1745	60	177	Male	0	1.7364
1746	61	177	Female	6	1.3249
1747	61	177	Female	6	1.3574
1748	61	177	Female	7	1.4087
1749	61	177	Male	0	1.1032
1750	61	177	Male	0	1.2013
1751	61	177	Male	0	1.2921
1752	65	177	Male	0	1.2152
1753	67	177	Female	6	1.0411
1754	67	177	Female	6	1.1692
1755	67	177	Male	0	1.3017
1756	67	177	Male	0	1.3526
1757	67	177	Male	0	1.3784
1758	69	177	Female	6	1.1887
1759	69	177	Male	0	1.2512
1760	69	177	Male	0	1.3041
1761	69	177	Male	0	1.3259
1762	70	177	Male	0	1.3087
1763	70	177	Male	0	1.3167
1764	70	177	Male	0	1.3291
1765	73	177	Female	6	1.3188
1766	73	177	Female	7	1.2743
1767	73	177	Female	7	1.3303
1768	73	177	Female	7	1.3502
1769	73	177	Male	0	1.1026
1770	73	177	Male	0	1.4162
1771	74	177	Female	8	1.3439
1772	74	177	Female	9	1.4299
1773	74	177	Female	9	1.4309
1774	74	177	Female	10	1.3851
1775	74	177	Male	0	1.3698
1776	75	177	Female	7	1.2156
1777	75	177	Female	8	1.2304
1778	75	177	Female	8	1.3360
1779	75	177	Male	0	1.2053
1780	75	177	Male	0	1.2094
1781	75	177	Male	0	1.2177
1782	75	177	Male	0	1.2577
1783	75	177	Male	0	1.2986

1784	75	177	Male	0	1.3142
1785	76	177	Female	6	1.2613
1786	76	177	Female	7	1.2297
1787	76	177	Female	8	0.8701
1788	76	177	Female	8	1.3009
1789	76	177	Male	0	1.1679
1790	76	177	Male	0	1.1946
1791	76	177	Male	0	1.2308
1792	76	177	Male	0	1.2383
1793	77	177	Female	6	1.2096
1794	77	177	Female	8	1.2123
1795	77	177	Male	0	1.0854
1796	77	177	Male	0	1.2274
1797	77	177	Male	0	1.2451
1798	81	177	Female	8	1.2993
1799	81	177	Male	0	1.3038
1800	81	177	Male	0	1.3079
1801	81	177	Male	0	1.3221
1802	81	177	Male	0	1.3241
1803	81	177	Male	0	1.3601
1804	83	177	Female	6	1.1599
1805	83	177	Female	7	1.1697
1806	83	177	Male	0	1.2198
1807	84	177	Female	7	1.2697
1808	84	177	Female	8	1.3046
1809	84	177	Male	0	1.2436
1810	86	177	Female	6	1.1916
1811	86	177	Female	6	1.2341
1812	86	177	Female	7	1.2394
1813	86	177	Male	0	1.0931
1814	86	177	Male	0	1.1917
1815	86	177	Male	0	1.2548
1816	86	177	Male	0	1.2923
1817	87	177	Female	7	1.4364
1818	87	177	Male	0	1.4253
1819	87	177	Male	0	1.4571
1820	88	177	Male	0	1.2863
1821	88	177	Male	0	1.3633
1822	90	177	Female	6	1.3823
1823	90	177	Female	6	1.4026
1824	90	177	Female	7	1.3626
1825	90	177	Female	7	1.4208
1826	90	177	Female	7	1.4261
1827	90	177	Female	8	1.3441

1828	90	177	Female	9	1.4407
1829	90	177	Female	10	1.3646
1830	90	177	Male	0	1.1293
1831	90	177	Male	0	1.1573
1832	90	177	Male	0	1.2911
1833	90	177	Male	0	1.3934
1834	90	177	Male	0	1.3988
1835	90	177	Male	0	1.4174
1836	90	177	Male	0	1.4201
1837	90	177	Male	0	1.4296
1838	90	177	Male	0	1.5095
1839	91	177	Female	8	1.3811
1840	91	177	Female	8	1.4977
1841	91	177	Male	0	1.2211
1842	91	177	Male	0	1.4971
1843	91	177	Male	0	1.5046
1844	91	177	Male	0	1.5661
1845	96	177	Female	6	1.1591
1846	96	177	Female	8	1.0911
1847	96	177	Female	8	1.2601
1848	97	177	Female	6	1.0184
1849	97	177	Female	6	1.1205
1850	97	177	Female	7	1.2455
1851	97	177	Female	8	1.0673
1852	97	177	Female	8	1.3073
1853	97	177	Female	9	1.2234
1854	97	177	Male	0	1.2126
1855	97	177	Male	0	1.2674
1856	99	177	Female	8	1.2498
1857	99	177	Male	0	1.1104
1858	99	177	Male	0	1.2714
1859	101	177	Female	8	1.2248
1860	101	177	Female	10	1.4294
1861	101	177	Male	0	1.2181
1862	101	177	Male	0	1.3063
1863	101	177	Male	0	1.3996
1864	102	177	Female	6	1.4891
1865	102	177	Female	7	1.4376
1866	102	177	Female	8	1.4927
1867	102	177	Female	9	1.4856
1868	102	177	Female	9	1.5632
1869	102	177	Female	9	1.6387
1870	102	177	Female	10	1.4764
1871	102	177	Male	0	1.3749

1872	102	177	Male	0	1.4161
1873	102	177	Male	0	1.4167
1874	102	177	Male	0	1.4369
1875	102	177	Male	0	1.4421
1876	102	177	Male	0	1.4581
1877	102	177	Male	0	1.4963
1878	102	177	Male	0	1.5026
1879	102	177	Male	0	1.5657
1880	102	177	Male	0	1.5972
1881	104	177	Male	0	1.1739
1882	105	177	Male	0	1.2309
1883	106	177	Female	7	1.3613
1884	106	177	Female	9	1.2318
1885	106	177	Female	9	1.3193
1886	106	177	Female	9	1.3584
1887	106	177	Male	0	1.3087
1888	106	177	Male	0	1.3149
1889	106	177	Male	0	1.3488
1890	106	177	Male	0	1.3610
1891	106	177	Male	0	1.3791
1892	106	177	Male	0	1.3824
1893	106	177	Male	0	1.3979
1894	106	177	Male	0	1.4201
1895	106	177	Male	0	1.4491
1896	107	177	Female	6	0.9946
1897	107	177	Female	6	1.2251
1898	107	177	Female	7	1.2464
1899	107	177	Female	7	1.2517
1900	107	177	Female	8	1.3113
1901	107	177	Female	9	1.2496
1902	107	177	Male	0	1.2552
1903	107	177	Male	0	1.2622
1904	200	177	Female	6	1.6227
1905	200	177	Male	0	1.3050
1906	200	177	Male	0	1.3893
1907	200	177	Male	0	1.4887
1908	200	177	Male	0	1.5286
1909	201	177	Female	9	1.2614
1910	201	177	Male	0	1.1743
1911	201	177	Male	0	1.2067
1912	201	177	Male	0	1.2274
1913	204	177	Female	6	1.3653
1914	204	177	Female	6	1.4578
1915	204	177	Female	9	1.3623

1916	204	177	Male	0	1.4093
1917	204	177	Male	0	1.4563
1918	204	177	Male	0	1.4652
1919	207	177	Male	0	1.4054
1920	207	177	Male	0	1.5104
1921	208	177	Male	0	1.5375
1922	208	177	Male	0	1.5429
1923	210	177	Male	0	1.3854
1924	211	177	Female	7	1.2610
1925	211	177	Female	8	1.2271
1926	211	177	Male	0	1.3972
1927	212	177	Female	7	1.3242
1928	212	177	Female	7	1.5114
1929	212	177	Female	8	1.2298
1930	212	177	Female	8	1.2443
1931	212	177	Female	8	1.2609
1932	212	177	Male	0	1.2429
1933	212	177	Male	0	1.2611
1934	212	177	Male	0	1.2843
1935	212	177	Male	0	1.3068
1936	212	177	Male	0	1.3123
1937	212	177	Male	0	1.3326
1938	212	177	Male	0	1.3335
1939	212	177	Male	0	1.3468
1940	212	177	Male	0	1.4553
1941	212	177	Male	0	1.4612
1942	213	177	Male	0	1.0081
1943	215	177	Male	0	1.3931
1944	215	177	Male	0	1.3974
1945	215	177	Male	0	1.5858
1946	217	177	Female	8	1.2931
1947	217	177	Female	9	1.2871
1948	217	177	Female	9	1.3803
1949	217	177	Female	10	1.3176
1950	217	177	Male	0	1.3553
1951	219	177	Male	0	1.2394
1952	219	177	Male	0	1.3980
1953	222	177	Male	0	1.1768
1954	222	177	Male	0	1.3055
1955	223	177	Female	6	1.1678
1956	223	177	Female	6	1.3067
1957	223	177	Female	6	1.3570
1958	223	177	Male	0	1.2011
1959	226	177	Female	6	1.3426

1960	226	177	Female	8	1.3053
1961	226	177	Male	0	1.1672
1962	227	177	Female	8	1.4280
1963	227	177	Male	0	1.1836
1964	227	177	Male	0	1.2157
1965	227	177	Male	0	1.3689
1966	227	177	Male	0	1.3900
1967	228	177	Female	7	1.3641
1968	228	177	Male	0	1.1360
1969	228	177	Male	0	1.1637
1970	228	177	Male	0	1.1922
1971	228	177	Male	0	1.2105
1972	228	177	Male	0	1.2249
1973	228	177	Male	0	1.2880
1974	228	177	Male	0	1.3316
1975	228	177	Male	0	1.3570
1976	1	178	Male	0	1.3380
1977	1	178	Male	0	1.4092
1978	1	178	Male	0	1.5314
1979	4	178	Female	6	1.3591
1980	4	178	Female	7	1.5310
1981	4	178	Female	8	1.4649
1982	4	178	Male	0	1.3256
1983	4	178	Male	0	1.5053
1984	4	178	Male	0	1.5686
1985	5	178	Female	9	1.2136
1986	5	178	Male	0	1.3568
1987	8	178	Male	0	1.2977
1988	8	178	Male	0	1.3688
1989	10	178	Female	6	1.0346
1990	11	178	Male	0	1.1041
1991	11	178	Male	0	1.3857
1992	12	178	Female	7	1.3546
1993	12	178	Male	0	1.2399
1994	12	178	Male	0	1.2525
1995	12	178	Male	0	1.2887
1996	13	178	Female	6	1.4110
1997	13	178	Female	8	1.2497
1998	13	178	Female	8	1.3093
1999	13	178	Female	9	1.3747
2000	13	178	Male	0	1.3457
2001	17	178	Female	7	1.2708
2002	17	178	Female	8	1.3743
2003	17	178	Female	9	1.3169

2004	17	178	Female	10	1.3087
2005	17	178	Male	0	1.2774
2006	17	178	Male	0	1.3068
2007	20	178	Female	9	1.2724
2008	20	178	Female	9	1.3432
2009	20	178	Male	0	1.1395
2010	20	178	Male	0	1.1819
2011	22	178	Female	8	1.3063
2012	22	178	Male	0	1.2291
2013	22	178	Male	0	1.2388
2014	22	178	Male	0	1.2791
2015	26	178	Female	6	1.3594
2016	26	178	Female	8	1.3949
2017	26	178	Female	8	1.4018
2018	26	178	Female	9	1.4197
2019	26	178	Male	0	1.2723
2020	26	178	Male	0	1.3214
2021	26	178	Male	0	1.3619
2022	31	178	Female	7	1.2358
2023	31	178	Female	8	1.3407
2024	31	178	Female	9	1.3528
2025	31	178	Female	10	1.2873
2026	31	178	Male	0	1.2897
2027	34	178	Female	7	1.3227
2028	34	178	Female	8	1.1988
2029	34	178	Female	8	1.2888
2030	34	178	Female	8	1.3113
2031	34	178	Female	8	1.3564
2032	34	178	Female	9	0.9702
2033	34	178	Female	9	1.0003
2034	34	178	Female	10	1.2197
2035	34	178	Male	0	1.2281
2036	35	178	Female	8	1.0483
2037	35	178	Female	8	1.3518
2038	35	178	Male	0	1.0299
2039	35	178	Male	0	1.0859
2040	35	178	Male	0	1.2623
2041	35	178	Male	0	1.3687
2042	36	178	Female	6	1.0327
2043	36	178	Female	7	1.2116
2044	36	178	Female	8	1.2333
2045	36	178	Female	8	1.2547
2046	36	178	Female	9	1.1247
2047	36	178	Female	9	1.2164

2048	36	178	Male	0	1.1410
2049	36	178	Male	0	1.2929
2050	37	178	Female	7	1.2919
2051	37	178	Female	7	1.3111
2052	37	178	Female	8	1.3621
2053	37	178	Male	0	1.2410
2054	37	178	Male	0	1.3517
2055	38	178	Female	6	1.2917
2056	38	178	Female	7	1.3372
2057	38	178	Male	0	1.2222
2058	46	178	Female	6	1.0078
2059	46	178	Female	7	1.1188
2060	46	178	Female	9	1.2073
2061	48	178	Female	6	1.1658
2062	48	178	Female	8	1.2235
2063	48	178	Male	0	1.1584
2064	48	178	Male	0	1.3419
2065	50	178	Female	7	1.2397
2066	50	178	Male	0	1.3723
2067	54	178	Female	8	1.1408
2068	54	178	Male	0	1.1664
2069	55	178	Female	7	1.2907
2070	55	178	Male	0	1.3452
2071	56	178	Male	0	1.1385
2072	56	178	Male	0	1.2751
2073	57	178	Female	6	1.2781
2074	59	178	Female	6	1.0034
2075	59	178	Female	7	1.1094
2076	59	178	Female	7	1.1362
2077	60	178	Female	6	2.1048
2078	60	178	Female	8	1.6941
2079	60	178	Female	8	1.8562
2080	60	178	Female	8	1.9633
2081	60	178	Female	9	1.6937
2082	60	178	Female	9	1.8839
2083	60	178	Female	10	1.6171
2084	60	178	Female	10	1.6234
2085	60	178	Female	10	1.7148
2086	60	178	Female	10	1.7456
2087	60	178	Male	0	1.2653
2088	60	178	Male	0	1.6123
2089	60	178	Male	0	1.8138
2090	60	178	Male	0	1.8309
2091	61	178	Female	8	1.3631

2092	61	178	Male	0	1.2801
2093	61	178	Male	0	1.4024
2094	62	178	Female	6	1.1781
2095	62	178	Female	6	1.1915
2096	62	178	Female	10	1.2627
2097	63	178	Female	7	1.3553
2098	64	178	Female	6	1.2872
2099	64	178	Female	7	1.3379
2100	64	178	Female	7	1.3939
2101	64	178	Female	8	1.3287
2102	64	178	Male	0	1.2537
2103	64	178	Male	0	1.2946
2104	64	178	Male	0	1.4136
2105	65	178	Female	6	1.2728
2106	65	178	Female	7	1.3119
2107	65	178	Female	9	1.2120
2108	65	178	Male	0	1.2183
2109	65	178	Male	0	1.2425
2110	65	178	Male	0	1.2438
2111	66	178	Male	0	1.0190
2112	66	178	Male	0	1.1006
2113	67	178	Female	6	1.2275
2114	67	178	Female	7	1.3097
2115	67	178	Female	8	1.2294
2116	67	178	Female	8	1.3886
2117	67	178	Male	0	0.9626
2118	67	178	Male	0	1.2787
2119	67	178	Male	0	1.2791
2120	69	178	Female	9	1.2975
2121	69	178	Male	0	1.3164
2122	70	178	Male	0	1.3139
2123	71	178	Female	8	1.1348
2124	73	178	Female	8	1.2693
2125	73	178	Male	0	1.3538
2126	74	178	Female	6	1.3279
2127	74	178	Female	7	1.3528
2128	74	178	Male	0	1.3699
2129	75	178	Female	6	1.2477
2130	75	178	Female	10	1.3619
2131	76	178	Female	7	1.2221
2132	76	178	Male	0	0.8433
2133	76	178	Male	0	1.1484
2134	77	178	Male	0	1.2139
2135	83	178	Female	7	1.1947

2136	83	178	Female	8	1.2315
2137	83	178	Male	0	1.2263
2138	83	178	Male	0	1.2494
2139	84	178	Female	6	1.2563
2140	84	178	Female	7	1.1899
2141	85	178	Male	0	1.2017
2142	86	178	Female	9	1.1793
2143	86	178	Male	0	1.3404
2144	87	178	Female	6	1.3498
2145	87	178	Male	0	1.1199
2146	90	178	Female	8	1.4106
2147	90	178	Female	8	1.4328
2148	90	178	Female	8	1.4684
2149	90	178	Male	0	1.4129
2150	90	178	Male	0	1.4162
2151	91	178	Female	10	1.5366
2152	96	178	Male	0	1.0891
2153	96	178	Male	0	1.2577
2154	97	178	Male	0	1.1236
2155	97	178	Male	0	1.2963
2156	97	178	Male	0	1.2981
2157	99	178	Male	0	1.3471
2158	101	178	Female	9	1.2342
2159	101	178	Female	9	1.4521
2160	102	178	Female	8	1.3926
2161	102	178	Female	8	1.4881
2162	102	178	Female	9	1.4331
2163	102	178	Female	10	1.4451
2164	104	178	Female	6	1.0213
2165	104	178	Male	0	1.1818
2166	105	178	Female	8	1.2280
2167	105	178	Male	0	1.2237
2168	105	178	Male	0	1.2279
2169	105	178	Male	0	1.2301
2170	106	178	Female	8	1.4202
2171	106	178	Male	0	1.3152
2172	106	178	Male	0	1.4775
2173	107	178	Female	6	0.9766
2174	107	178	Female	8	1.2304
2175	107	178	Male	0	1.2223
2176	107	178	Male	0	1.2459
2177	107	178	Male	0	1.3142
2178	200	178	Male	0	1.4004
2179	200	178	Male	0	1.4341

2180	200	178	Male	0	1.4707
2181	201	178	Female	8	1.3391
2182	202	178	Female	6	1.1881
2183	202	178	Male	0	1.0211
2184	204	178	Female	7	1.3283
2185	204	178	Female	9	1.4262
2186	204	178	Male	0	1.1758
2187	204	178	Male	0	1.4516
2188	204	178	Male	0	1.5365
2189	208	178	Male	0	1.5300
2190	210	178	Female	6	1.3904
2191	212	178	Female	11	1.2118
2192	217	178	Male	0	1.3304
2193	219	178	Male	0	1.1463
2194	220	178	Male	0	1.3167
2195	222	178	Female	8	1.5462
2196	222	178	Male	0	1.3806
2197	223	178	Female	8	1.1153
2198	224	178	Female	6	1.5102
2199	224	178	Female	6	1.5424
2200	226	178	Female	8	1.5169
2201	226	178	Male	0	1.1527
2202	226	178	Male	0	1.2724
2203	226	178	Male	0	1.3848
2204	226	178	Male	0	1.3888
2205	227	178	Female	7	1.1037
2206	228	178	Female	6	1.2728
2207	228	178	Female	6	1.3333
2208	228	178	Male	0	1.1328
2209	1	179	Female	6	1.3951
2210	1	179	Female	10	1.3691
2211	1	179	Male	0	1.3436
2212	1	179	Male	0	1.4118
2213	4	179	Female	7	1.5853
2214	4	179	Male	0	1.4807
2215	5	179	Female	6	1.3009
2216	5	179	Female	8	1.2627
2217	5	179	Female	8	1.4113
2218	5	179	Male	0	1.3844
2219	8	179	Female	9	1.2630
2220	10	179	Male	0	1.0255
2221	10	179	Male	0	1.1223
2222	10	179	Male	0	1.1413
2223	11	179	Female	6	1.2262

2224	12	179	Female	7	1.2646
2225	12	179	Female	7	1.2833
2226	12	179	Female	7	1.2908
2227	12	179	Female	7	1.3497
2228	12	179	Female	7	1.3562
2229	12	179	Female	8	1.3303
2230	12	179	Female	8	1.3583
2231	12	179	Male	0	1.3116
2232	14	179	Female	6	1.1964
2233	14	179	Female	7	1.3287
2234	15	179	Female	7	1.2767
2235	15	179	Female	7	1.2854
2236	15	179	Female	8	1.2967
2237	15	179	Male	0	1.1710
2238	15	179	Male	0	1.3908
2239	16	179	Male	0	1.1113
2240	17	179	Female	6	1.2354
2241	19	179	Female	6	1.3796
2242	19	179	Female	7	1.3793
2243	20	179	Female	7	1.3224
2244	20	179	Male	0	0.9859
2245	20	179	Male	0	1.2957
2246	20	179	Male	0	1.3506
2247	21	179	Female	7	1.2826
2248	21	179	Female	7	1.2928
2249	22	179	Male	0	1.2889
2250	24	179	Female	7	1.2776
2251	24	179	Female	7	1.3189
2252	24	179	Male	0	1.2841
2253	24	179	Male	0	1.3288
2254	24	179	Male	0	1.3767
2255	24	179	Male	0	1.4407
2256	26	179	Female	7	1.4144
2257	26	179	Female	8	1.4583
2258	26	179	Female	10	1.4191
2259	27	179	Female	7	1.2278
2260	27	179	Female	7	1.2943
2261	27	179	Female	9	1.2196
2262	27	179	Male	0	1.3327
2263	29	179	Male	0	1.3517
2264	31	179	Female	6	1.1237
2265	31	179	Female	6	1.3221
2266	31	179	Female	7	1.2011
2267	31	179	Female	10	1.2331

2268	31	179	Male	0	1.2763
2269	31	179	Male	0	1.2996
2270	31	179	Male	0	1.3309
2271	31	179	Male	0	1.3363
2272	32	179	Female	9	1.3653
2273	34	179	Female	7	1.2736
2274	34	179	Female	7	1.2846
2275	35	179	Female	6	1.2909
2276	35	179	Male	0	1.0194
2277	36	179	Female	6	1.2538
2278	36	179	Female	8	1.2169
2279	36	179	Female	8	1.2463
2280	36	179	Male	0	1.2246
2281	36	179	Male	0	1.2568
2282	36	179	Male	0	1.2918
2283	36	179	Male	0	1.4509
2284	37	179	Female	9	1.2026
2285	38	179	Female	4	1.3656
2286	38	179	Female	6	1.3282
2287	38	179	Male	0	1.3269
2288	42	179	Female	7	1.2258
2289	42	179	Male	0	1.0671
2290	48	179	Female	8	1.2765
2291	48	179	Male	0	1.2764
2292	50	179	Male	0	1.2368
2293	53	179	Female	6	1.2215
2294	53	179	Female	7	1.2784
2295	53	179	Female	8	1.3063
2296	53	179	Male	0	1.0503
2297	54	179	Female	8	1.2336
2298	54	179	Female	9	1.2205
2299	54	179	Male	0	1.3059
2300	56	179	Female	9	1.3457
2301	56	179	Female	10	1.1863
2302	56	179	Male	0	1.1465
2303	56	179	Male	0	1.2180
2304	57	179	Female	7	1.3234
2305	57	179	Female	8	1.2143
2306	57	179	Female	9	1.2508
2307	57	179	Male	0	1.2384
2308	57	179	Male	0	1.2719
2309	57	179	Male	0	1.2827
2310	60	179	Female	8	1.9453
2311	60	179	Female	9	1.7843

2312	60	179	Male	0	1.4384
2313	60	179	Male	0	1.6508
2314	60	179	Male	0	1.6822
2315	60	179	Male	0	1.7503
2316	61	179	Female	6	1.2981
2317	61	179	Female	7	1.3318
2318	61	179	Male	0	1.2237
2319	61	179	Male	0	1.3120
2320	63	179	Female	8	1.3182
2321	63	179	Male	0	1.3184
2322	64	179	Female	7	1.3557
2323	64	179	Female	7	1.3674
2324	64	179	Female	7	1.4332
2325	64	179	Male	0	1.3778
2326	65	179	Female	6	1.3174
2327	66	179	Female	6	1.2626
2328	66	179	Male	0	1.0358
2329	66	179	Male	0	1.0598
2330	67	179	Female	6	1.1694
2331	67	179	Female	10	1.2744
2332	67	179	Male	0	1.2482
2333	67	179	Male	0	1.2569
2334	73	179	Female	6	1.3304
2335	73	179	Female	6	1.3356
2336	73	179	Male	0	1.2166
2337	77	179	Male	0	1.2641
2338	81	179	Female	7	1.1871
2339	81	179	Female	7	1.3196
2340	81	179	Female	9	1.2348
2341	81	179	Female	9	1.3529
2342	81	179	Male	0	0.9861
2343	87	179	Female	7	1.3781
2344	87	179	Female	9	1.3884
2345	88	179	Male	0	1.2859
2346	91	179	Female	7	1.4164
2347	91	179	Female	7	1.4321
2348	98	179	Male	0	1.2763
2349	99	179	Female	6	1.2311
2350	99	179	Male	0	1.2641
2351	99	179	Male	0	1.3331
2352	101	179	Male	0	1.4186
2353	102	179	Female	7	1.4612
2354	102	179	Female	8	1.5049
2355	102	179	Female	9	1.5811

2356	105	179	Male	0	1.2140
2357	106	179	Female	9	1.3959
2358	106	179	Female	10	1.3868
2359	106	179	Male	0	1.3465
2360	107	179	Male	0	1.2184
2361	107	179	Male	0	1.2713
2362	200	179	Female	8	1.5264
2363	201	179	Female	7	1.2889
2364	211	179	Male	0	1.2319
2365	212	179	Female	6	1.3228
2366	218	179	Male	0	1.3671
2367	218	179	Male	0	1.3908
2368	219	179	Male	0	1.2590
2369	220	179	Female	6	1.2797
2370	220	179	Male	0	1.2889
2371	222	179	Female	7	1.2175
2372	222	179	Female	9	1.3437
2373	222	179	Female	9	1.5277
2374	222	179	Male	0	1.3294
2375	227	179	Male	0	1.1714
2376	227	179	Male	0	1.1888
2377	228	179	Female	6	1.4955
2378	228	179	Female	7	1.4156
2379	1	180	Female	6	1.3428
2380	1	180	Female	8	1.4158
2381	1	180	Male	0	1.3383
2382	3	180	Male	0	1.2109
2383	5	180	Male	0	1.3523
2384	8	180	Male	0	1.3367
2385	8	180	Male	0	1.3565
2386	12	180	Female	10	1.3029
2387	12	180	Male	0	1.2318
2388	13	180	Male	0	1.3459
2389	13	180	Male	0	1.4246
2390	14	180	Female	6	1.3012
2391	14	180	Male	0	1.1180
2392	15	180	Female	7	1.2124
2393	15	180	Female	8	1.2837
2394	15	180	Male	0	1.2921
2395	17	180	Female	6	1.2268
2396	17	180	Female	6	1.2769
2397	17	180	Female	7	1.2531
2398	18	180	Female	6	1.1517
2399	19	180	Male	0	1.4046

2400	20	180	Female	7	1.3093
2401	20	180	Male	0	1.2819
2402	21	180	Female	8	1.3707
2403	21	180	Female	8	1.3943
2404	21	180	Male	0	1.2684
2405	21	180	Male	0	1.3225
2406	22	180	Female	7	1.2904
2407	22	180	Female	9	1.2972
2408	24	180	Female	7	1.3793
2409	26	180	Male	0	1.3254
2410	26	180	Male	0	1.3575
2411	27	180	Male	0	1.1995
2412	27	180	Male	0	1.3092
2413	29	180	Female	7	1.4184
2414	31	180	Male	0	1.4221
2415	34	180	Male	0	1.2203
2416	35	180	Female	7	1.4092
2417	36	180	Female	6	1.1911
2418	36	180	Female	7	1.1723
2419	36	180	Female	7	1.1923
2420	37	180	Female	9	1.3668
2421	37	180	Male	0	1.3442
2422	38	180	Female	6	1.1791
2423	38	180	Male	0	1.2091
2424	38	180	Male	0	1.3593
2425	42	180	Male	0	1.1823
2426	42	180	Male	0	1.2795
2427	43	180	Female	7	1.1954
2428	43	180	Female	8	1.2722
2429	43	180	Male	0	1.2122
2430	48	180	Male	0	1.1797
2431	50	180	Female	9	1.3231
2432	50	180	Male	0	1.2488
2433	51	180	Female	8	1.2643
2434	53	180	Female	8	1.1654
2435	53	180	Male	0	1.2191
2436	55	180	Female	7	1.1431
2437	55	180	Female	8	1.1372
2438	55	180	Female	8	1.3448
2439	55	180	Male	0	1.3324
2440	56	180	Female	9	1.2996
2441	56	180	Male	0	1.1564
2442	57	180	Female	6	1.2207
2443	57	180	Female	6	1.2352

2444	59	180	Female	7	1.0937
2445	60	180	Female	10	1.5747
2446	60	180	Male	0	1.4950
2447	61	180	Female	6	1.3362
2448	61	180	Female	7	1.2392
2449	61	180	Male	0	1.2027
2450	61	180	Male	0	1.3216
2451	62	180	Female	6	1.1313
2452	62	180	Female	6	1.1693
2453	62	180	Female	8	1.1624
2454	63	180	Female	6	1.2839
2455	63	180	Female	7	1.3233
2456	63	180	Female	8	1.2966
2457	64	180	Male	0	1.3574
2458	64	180	Male	0	1.4012
2459	67	180	Female	6	1.2546
2460	70	180	Female	8	1.3496
2461	70	180	Male	0	1.3217
2462	71	180	Female	6	1.2016
2463	71	180	Male	0	1.1486
2464	73	180	Female	8	1.3198
2465	74	180	Female	6	1.3501
2466	74	180	Female	7	1.3573
2467	74	180	Female	7	1.4451
2468	74	180	Female	8	1.3653
2469	74	180	Male	0	1.1864
2470	75	180	Female	6	1.2860
2471	75	180	Female	8	1.3778
2472	75	180	Male	0	1.1793
2473	75	180	Male	0	1.3783
2474	81	180	Male	0	1.1056
2475	83	180	Female	7	1.1821
2476	83	180	Female	7	1.2253
2477	86	180	Female	9	1.2617
2478	86	180	Female	9	1.3222
2479	86	180	Male	0	0.9365
2480	86	180	Male	0	1.2472
2481	87	180	Female	8	1.4461
2482	88	180	Male	0	1.3134
2483	90	180	Female	7	1.4028
2484	91	180	Female	6	1.3812
2485	91	180	Female	6	1.4511
2486	91	180	Female	6	1.5046
2487	91	180	Female	7	1.5179

2488	97	180	Male	0	1.1888
2489	97	180	Male	0	1.2554
2490	98	180	Male	0	1.0287
2491	98	180	Male	0	1.2081
2492	99	180	Female	8	1.2671
2493	99	180	Male	0	1.0301
2494	99	180	Male	0	1.1244
2495	99	180	Male	0	1.2698
2496	99	180	Male	0	1.3001
2497	101	180	Female	5	1.0644
2498	101	180	Female	8	1.2784
2499	101	180	Male	0	1.3371
2500	102	180	Female	7	1.4706
2501	102	180	Female	7	1.4781
2502	102	180	Female	8	1.4891
2503	102	180	Male	0	1.4484
2504	102	180	Male	0	1.4586
2505	102	180	Male	0	1.4983
2506	102	180	Male	0	1.5242
2507	104	180	Female	7	1.2223
2508	104	180	Male	0	1.1781
2509	105	180	Male	0	1.2266
2510	106	180	Female	8	1.3867
2511	106	180	Male	0	1.3024
2512	106	180	Male	0	1.4775
2513	107	180	Female	9	1.2116
2514	200	180	Female	8	1.5867
2515	200	180	Male	0	1.3971
2516	200	180	Male	0	1.4693
2517	200	180	Male	0	1.5001
2518	200	180	Male	0	1.5328
2519	204	180	Female	6	1.4132
2520	204	180	Female	7	1.4239
2521	204	180	Male	0	1.3354
2522	204	180	Male	0	1.4513
2523	204	180	Male	0	1.4757
2524	205	180	Male	0	1.3496
2525	206	180	Female	6	1.4671
2526	206	180	Male	0	1.3770
2527	209	180	Male	0	1.3962
2528	211	180	Female	6	1.4423
2529	211	180	Female	7	1.3165
2530	211	180	Male	0	1.2185
2531	211	180	Male	0	1.2962

2532	211	180	Male	0	1.3529
2533	212	180	Male	0	1.2201
2534	215	180	Female	7	1.5336
2535	218	180	Male	0	1.3232
2536	220	180	Female	6	1.3104
2537	222	180	Female	6	1.3974
2538	222	180	Male	0	1.3995
2539	223	180	Male	0	1.4005
2540	224	180	Female	7	1.3947
2541	224	180	Male	0	1.2718
2542	224	180	Male	0	1.2840
2543	226	180	Female	7	1.4934
2544	226	180	Male	0	1.3361
2545	1	181	Female	9	1.3276
2546	1	181	Female	9	1.4809
2547	1	181	Male	0	1.3643
2548	1	181	Male	0	1.4226
2549	4	181	Male	0	1.5163
2550	4	181	Male	0	1.5163
2551	7	181	Female	6	1.0863
2552	8	181	Female	8	1.2693
2553	8	181	Male	0	1.1655
2554	8	181	Male	0	1.2856
2555	12	181	Female	8	1.3499
2556	12	181	Female	10	1.2263
2557	12	181	Male	0	1.4207
2558	13	181	Male	0	1.3458
2559	14	181	Male	0	1.2814
2560	14	181	Male	0	1.3229
2561	15	181	Female	6	1.3536
2562	15	181	Female	6	1.3881
2563	16	181	Female	7	1.2082
2564	16	181	Male	0	1.2234
2565	18	181	Male	0	1.2593
2566	19	181	Male	0	1.2982
2567	19	181	Male	0	1.4020
2568	20	181	Female	7	1.2572
2569	20	181	Male	0	1.3094
2570	20	181	Male	0	1.3111
2571	21	181	Female	6	1.2587
2572	21	181	Female	6	1.3093
2573	21	181	Male	0	1.4462
2574	24	181	Male	0	1.1867
2575	24	181	Male	0	1.2187

2576	26	181	Female	8	1.3949
2577	26	181	Male	0	1.3062
2578	27	181	Male	0	1.1620
2579	29	181	Female	7	1.3477
2580	29	181	Male	0	1.3532
2581	29	181	Male	0	1.3749
2582	32	181	Female	7	1.3617
2583	32	181	Female	9	1.3452
2584	32	181	Male	0	1.3421
2585	32	181	Male	0	1.4398
2586	34	181	Female	8	1.2982
2587	34	181	Male	0	1.2083
2588	34	181	Male	0	1.3093
2589	35	181	Male	0	1.0217
2590	36	181	Female	8	0.9545
2591	36	181	Female	8	1.2238
2592	36	181	Male	0	1.2219
2593	36	181	Male	0	1.2598
2594	36	181	Male	0	1.3276
2595	37	181	Male	0	1.3043
2596	38	181	Female	6	1.2104
2597	38	181	Male	0	1.1797
2598	38	181	Male	0	1.3221
2599	38	181	Male	0	1.3668
2600	42	181	Female	6	1.1613
2601	42	181	Female	6	1.2171
2602	42	181	Male	0	1.1294
2603	42	181	Male	0	1.2740
2604	48	181	Male	0	1.2271
2605	50	181	Female	7	1.2124
2606	50	181	Female	7	1.3051
2607	55	181	Female	7	1.2708
2608	55	181	Female	7	1.3403
2609	55	181	Female	8	1.2789
2610	55	181	Female	8	1.3342
2611	56	181	Female	9	1.3268
2612	57	181	Female	7	1.2896
2613	60	181	Female	6	1.8982
2614	60	181	Female	10	1.5178
2615	60	181	Female	10	1.6185
2616	60	181	Male	0	1.1577
2617	60	181	Male	0	1.6259
2618	60	181	Male	0	1.7226
2619	60	181	Male	0	1.8426

2620	61	181	Female	7	1.2338
2621	61	181	Female	7	1.3762
2622	61	181	Male	0	1.3171
2623	62	181	Female	9	1.1917
2624	64	181	Female	6	1.2063
2625	64	181	Female	6	1.2588
2626	65	181	Female	6	1.2117
2627	65	181	Female	7	1.2571
2628	66	181	Female	7	1.2396
2629	67	181	Female	6	1.3669
2630	67	181	Male	0	1.2148
2631	67	181	Male	0	1.2714
2632	67	181	Male	0	1.2714
2633	67	181	Male	0	1.3317
2634	67	181	Male	0	1.3394
2635	67	181	Male	0	1.3958
2636	67	181	Male	0	1.3958
2637	69	181	Male	0	1.1976
2638	69	181	Male	0	1.2072
2639	69	181	Male	0	1.2796
2640	69	181	Male	0	1.2843
2641	70	181	Female	6	1.2055
2642	70	181	Female	6	1.2346
2643	71	181	Female	8	1.1709
2644	74	181	Female	7	1.3683
2645	74	181	Female	7	1.4003
2646	74	181	Male	0	1.3315
2647	75	181	Female	6	1.1418
2648	75	181	Female	8	1.2588
2649	76	181	Female	6	1.2657
2650	77	181	Male	0	1.1909
2651	77	181	Male	0	1.2283
2652	77	181	Male	0	1.2387
2653	81	181	Male	0	1.3436
2654	81	181	Male	0	1.3602
2655	84	181	Female	8	1.2221
2656	86	181	Male	0	1.1914
2657	86	181	Male	0	1.2469
2658	86	181	Male	0	1.2757
2659	87	181	Female	6	1.2795
2660	87	181	Male	0	1.3689
2661	88	181	Female	7	1.3493
2662	88	181	Male	0	1.2179
2663	90	181	Female	7	1.3869

2664	90	181	Female	7	1.3886
2665	90	181	Male	0	1.3814
2666	90	181	Male	0	1.4184
2667	91	181	Female	7	1.5656
2668	91	181	Male	0	1.5012
2669	97	181	Male	0	1.1715
2670	97	181	Male	0	1.1905
2671	97	181	Male	0	1.2076
2672	98	181	Female	7	1.0911
2673	99	181	Female	6	1.1119
2674	102	181	Female	6	1.4550
2675	102	181	Male	0	1.4981
2676	102	181	Male	0	1.5348
2677	103	181	Female	7	1.0005
2678	106	181	Female	6	1.2344
2679	106	181	Female	7	1.3184
2680	106	181	Female	7	1.3707
2681	106	181	Female	7	1.3839
2682	106	181	Female	9	1.3359
2683	106	181	Female	9	1.4055
2684	106	181	Male	0	1.3334
2685	106	181	Male	0	1.3744
2686	107	181	Female	6	1.2391
2687	107	181	Male	0	1.2583
2688	107	181	Male	0	1.2862
2689	200	181	Female	7	1.3868
2690	200	181	Male	0	1.3954
2691	204	181	Female	6	1.3937
2692	204	181	Female	7	1.1999
2693	204	181	Male	0	1.3332
2694	205	181	Female	7	1.3061
2695	206	181	Male	0	1.3578
2696	206	181	Male	0	1.4125
2697	207	181	Male	0	1.4946
2698	210	181	Female	7	1.3828
2699	211	181	Female	8	1.3744
2700	211	181	Male	0	1.2250
2701	211	181	Male	0	1.3273
2702	211	181	Male	0	1.3785
2703	211	181	Male	0	1.3971
2704	211	181	Male	0	1.4628
2705	213	181	Female	7	1.3512
2706	215	181	Male	0	1.4891
2707	222	181	Female	7	1.2614

2708	226	181	Female	9	1.0616
2709	227	181	Female	7	1.1790
2710	1	184	Female	8	1.0459
2711	1	184	Female	8	1.3091
2712	1	184	Female	8	1.3324
2713	1	184	Female	8	1.3918
2714	1	184	Female	8	1.4477
2715	1	184	Female	9	1.1547
2716	1	184	Female	9	1.4533
2717	1	184	Female	10	1.4746
2718	1	184	Male	0	1.0985
2719	1	184	Male	0	1.1117
2720	1	184	Male	0	1.2570
2721	1	184	Male	0	1.2579
2722	1	184	Male	0	1.3391
2723	1	184	Male	0	1.4159
2724	1	184	Male	0	1.4503
2725	1	184	Male	0	1.4608
2726	1	184	Male	0	1.4725
2727	1	184	Male	0	1.4765
2728	4	184	Female	7	1.4536
2729	4	184	Female	8	1.4611
2730	4	184	Male	0	1.4933
2731	4	184	Male	0	1.5019
2732	4	184	Male	0	1.5231
2733	4	184	Male	0	1.5483
2734	4	184	Male	0	1.5700
2735	5	184	Female	7	1.3453
2736	5	184	Female	8	1.3932
2737	5	184	Male	0	1.0152
2738	5	184	Male	0	1.2533
2739	5	184	Male	0	1.3382
2740	5	184	Male	0	1.3645
2741	5	184	Male	0	1.3674
2742	5	184	Male	0	1.3994
2743	5	184	Male	0	1.4218
2744	7	184	Male	0	1.2454
2745	8	184	Male	0	1.1137
2746	8	184	Male	0	1.1253
2747	11	184	Female	6	1.3166
2748	11	184	Female	6	1.3458
2749	11	184	Female	7	1.4051
2750	11	184	Female	7	1.4263
2751	11	184	Female	8	1.2777

2752	11	184	Female	8	1.3346
2753	11	184	Female	8	1.3551
2754	11	184	Female	8	1.3742
2755	11	184	Male	0	1.3796
2756	11	184	Male	0	1.4032
2757	12	184	Female	6	1.3549
2758	12	184	Female	8	1.3148
2759	12	184	Male	0	1.2356
2760	12	184	Male	0	1.2380
2761	12	184	Male	0	1.3258
2762	12	184	Male	0	1.3504
2763	12	184	Male	0	1.3963
2764	13	184	Female	9	1.3197
2765	14	184	Female	8	1.3059
2766	15	184	Male	0	1.1581
2767	15	184	Male	0	1.2056
2768	15	184	Male	0	1.4205
2769	16	184	Female	6	1.3173
2770	17	184	Male	0	1.2978
2771	18	184	Female	6	1.3608
2772	18	184	Female	7	1.2124
2773	18	184	Male	0	1.2300
2774	20	184	Female	6	1.2298
2775	20	184	Female	8	1.2835
2776	20	184	Male	0	1.2499
2777	20	184	Male	0	1.2553
2778	20	184	Male	0	1.2724
2779	20	184	Male	0	1.3267
2780	20	184	Male	0	1.3514
2781	21	184	Female	6	1.2484
2782	21	184	Female	7	1.1739
2783	21	184	Female	7	1.3601
2784	21	184	Male	0	1.3320
2785	21	184	Male	0	1.3642
2786	21	184	Male	0	1.3929
2787	21	184	Male	0	1.4044
2788	21	184	Male	0	1.4218
2789	22	184	Female	8	1.3693
2790	22	184	Male	0	1.0173
2791	22	184	Male	0	1.2335
2792	24	184	Female	8	1.0353
2793	26	184	Female	6	1.2790
2794	26	184	Female	6	1.4738
2795	26	184	Female	10	1.4364

2796	26	184	Male	0	1.3914
2797	26	184	Male	0	1.4610
2798	27	184	Female	8	1.1451
2799	27	184	Female	8	1.2117
2800	27	184	Female	9	1.2423
2801	27	184	Male	0	1.2156
2802	29	184	Female	6	1.3565
2803	29	184	Female	6	1.3884
2804	29	184	Female	7	1.3226
2805	29	184	Female	7	1.3428
2806	29	184	Female	7	1.3671
2807	29	184	Male	0	1.2669
2808	29	184	Male	0	1.3284
2809	29	184	Male	0	1.3776
2810	29	184	Male	0	1.3856
2811	31	184	Female	7	1.2206
2812	31	184	Female	8	1.3586
2813	31	184	Female	9	1.3124
2814	31	184	Female	9	1.4192
2815	31	184	Male	0	1.3188
2816	32	184	Male	0	1.1360
2817	34	184	Female	7	1.1401
2818	34	184	Male	0	1.2177
2819	35	184	Female	7	1.4018
2820	35	184	Female	9	1.2664
2821	35	184	Female	9	1.3881
2822	35	184	Male	0	1.2310
2823	35	184	Male	0	1.2542
2824	36	184	Female	7	1.2224
2825	36	184	Male	0	1.2299
2826	37	184	Female	7	1.3156
2827	37	184	Female	7	1.3273
2828	37	184	Female	7	1.4811
2829	37	184	Female	8	1.2976
2830	37	184	Female	10	1.3015
2831	37	184	Male	0	1.3205
2832	37	184	Male	0	1.3460
2833	38	184	Female	6	1.1720
2834	38	184	Female	6	1.1762
2835	38	184	Female	6	1.2977
2836	38	184	Female	7	1.2709
2837	38	184	Female	7	1.3523
2838	38	184	Male	0	1.1080
2839	38	184	Male	0	1.2329

2840	38	184	Male	0	1.2557
2841	38	184	Male	0	1.2727
2842	42	184	Female	6	1.0768
2843	42	184	Male	0	1.1178
2844	42	184	Male	0	1.2239
2845	42	184	Male	0	1.2581
2846	43	184	Male	0	1.2719
2847	43	184	Male	0	1.2997
2848	48	184	Male	0	1.2247
2849	48	184	Male	0	1.2921
2850	48	184	Male	0	1.2981
2851	48	184	Male	0	1.3273
2852	50	184	Female	6	1.4284
2853	50	184	Female	8	1.3008
2854	51	184	Male	0	1.2907
2855	53	184	Male	0	1.1183
2856	53	184	Male	0	1.1750
2857	53	184	Male	0	1.2187
2858	54	184	Female	6	1.2487
2859	54	184	Female	6	1.2539
2860	54	184	Female	6	1.2727
2861	57	184	Male	0	1.2763
2862	57	184	Male	0	1.2891
2863	60	184	Female	7	1.5337
2864	60	184	Female	9	1.7178
2865	60	184	Female	9	1.8132
2866	60	184	Female	9	1.9844
2867	60	184	Male	0	1.3112
2868	60	184	Male	0	1.3484
2869	60	184	Male	0	1.4743
2870	60	184	Male	0	1.4986
2871	60	184	Male	0	1.5423
2872	60	184	Male	0	1.7679
2873	60	184	Male	0	1.7858
2874	60	184	Male	0	1.8563
2875	61	184	Female	6	1.3321
2876	61	184	Female	8	1.3783
2877	61	184	Male	0	1.2640
2878	61	184	Male	0	1.2824
2879	61	184	Male	0	1.2898
2880	61	184	Male	0	1.3547
2881	62	184	Male	0	0.9693
2882	63	184	Female	6	1.2536
2883	63	184	Female	8	1.2669

2884	63	184	Female	9	1.2481
2885	63	184	Male	0	1.3194
2886	63	184	Male	0	1.5086
2887	64	184	Female	6	1.4111
2888	64	184	Male	0	1.3266
2889	64	184	Male	0	1.3816
2890	64	184	Male	0	1.4052
2891	64	184	Male	0	1.4218
2892	64	184	Male	0	1.4306
2893	64	184	Male	0	1.4322
2894	65	184	Female	7	1.3414
2895	65	184	Male	0	1.2352
2896	66	184	Female	7	1.2779
2897	66	184	Male	0	1.1280
2898	67	184	Male	0	1.2148
2899	67	184	Male	0	1.3076
2900	67	184	Male	0	1.3209
2901	67	184	Male	0	1.3394
2902	67	184	Male	0	1.4653
2903	69	184	Male	0	1.1341
2904	69	184	Male	0	1.2076
2905	69	184	Male	0	1.2832
2906	69	184	Male	0	1.3219
2907	70	184	Female	6	1.3718
2908	70	184	Male	0	1.2833
2909	70	184	Male	0	1.3099
2910	70	184	Male	0	1.3393
2911	70	184	Male	0	1.3497
2912	74	184	Female	6	1.4204
2913	74	184	Female	7	1.2458
2914	74	184	Female	7	1.3203
2915	74	184	Female	7	1.3687
2916	74	184	Female	8	1.3097
2917	74	184	Female	8	1.3326
2918	74	184	Female	8	1.3495
2919	74	184	Male	0	0.9829
2920	74	184	Male	0	1.0130
2921	74	184	Male	0	1.0437
2922	74	184	Male	0	1.0540
2923	74	184	Male	0	1.0974
2924	74	184	Male	0	1.2178
2925	74	184	Male	0	1.3198
2926	75	184	Female	6	1.4167
2927	75	184	Male	0	1.2124

2928	75	184	Male	0	1.2287
2929	75	184	Male	0	1.3796
2930	76	184	Female	7	1.2597
2931	76	184	Male	0	1.0601
2932	76	184	Male	0	1.1996
2933	77	184	Female	7	1.2427
2934	77	184	Male	0	1.1543
2935	78	184	Female	6	1.3859
2936	78	184	Female	8	1.2609
2937	78	184	Female	8	1.3929
2938	78	184	Male	0	1.4877
2939	79	184	Female	6	1.2383
2940	81	184	Male	0	1.3331
2941	83	184	Female	6	1.0181
2942	84	184	Male	0	1.3219
2943	86	184	Female	8	1.2929
2944	86	184	Female	10	1.3123
2945	86	184	Male	0	0.9673
2946	86	184	Male	0	0.9804
2947	86	184	Male	0	1.1898
2948	87	184	Female	6	1.1574
2949	87	184	Female	7	1.2829
2950	87	184	Female	7	1.4679
2951	87	184	Female	8	1.4001
2952	87	184	Female	8	1.4391
2953	88	184	Female	6	1.1845
2954	88	184	Female	7	1.2294
2955	90	184	Female	6	1.3851
2956	90	184	Female	6	1.4032
2957	90	184	Female	6	1.4044
2958	90	184	Female	8	1.3946
2959	90	184	Female	9	1.3814
2960	90	184	Male	0	1.1604
2961	90	184	Male	0	1.2086
2962	90	184	Male	0	1.2226
2963	90	184	Male	0	1.3289
2964	90	184	Male	0	1.3396
2965	91	184	Female	6	1.4329
2966	91	184	Female	6	1.4567
2967	91	184	Female	6	1.5234
2968	91	184	Female	7	1.4579
2969	91	184	Male	0	1.3969
2970	91	184	Male	0	1.4704
2971	91	184	Male	0	1.4989

2972	92	184	Female	6	1.1101
2973	92	184	Female	8	1.3211
2974	97	184	Female	7	1.3553
2975	97	184	Female	8	1.2528
2976	97	184	Female	8	1.2746
2977	97	184	Male	0	1.2337
2978	97	184	Male	0	1.2448
2979	97	184	Male	0	1.2643
2980	98	184	Female	8	1.1951
2981	98	184	Male	0	1.3081
2982	99	184	Male	0	1.0537
2983	99	184	Male	0	1.3014
2984	100	184	Female	7	1.2098
2985	101	184	Female	8	1.1616
2986	101	184	Female	8	1.2410
2987	101	184	Female	9	1.3384
2988	101	184	Male	0	1.3867
2989	101	184	Male	0	1.3981
2990	101	184	Male	0	1.4960
2991	102	184	Female	7	1.6178
2992	102	184	Female	8	1.4776
2993	102	184	Female	8	1.4786
2994	102	184	Female	8	1.4951
2995	102	184	Female	9	1.4764
2996	102	184	Female	9	1.5929
2997	103	184	Female	6	0.9686
2998	104	184	Female	6	1.0998
2999	104	184	Female	8	1.1599
3000	104	184	Female	8	1.2124
3001	105	184	Male	0	1.2186
3002	106	184	Female	10	1.3687
3003	107	184	Female	7	1.2613
3004	107	184	Female	9	1.3803
3005	200	184	Female	6	1.4404
3006	200	184	Female	7	1.3501
3007	200	184	Female	7	1.4173
3008	200	184	Female	7	1.5157
3009	200	184	Female	8	1.5530
3010	200	184	Male	0	1.2693
3011	200	184	Male	0	1.3606
3012	200	184	Male	0	1.4121
3013	200	184	Male	0	1.4808
3014	200	184	Male	0	1.4886
3015	200	184	Male	0	1.5106

3016	200	184	Male	0	1.5684
3017	200	184	Male	0	1.6515
3018	201	184	Female	7	1.2024
3019	201	184	Female	8	1.1964
3020	201	184	Male	0	1.2457
3021	202	184	Male	0	1.2569
3022	204	184	Female	6	1.5346
3023	204	184	Male	0	1.3926
3024	204	184	Male	0	1.3976
3025	204	184	Male	0	1.4129
3026	204	184	Male	0	1.4797
3027	204	184	Male	0	1.4943
3028	205	184	Female	6	1.3248
3029	205	184	Female	7	1.3857
3030	205	184	Female	7	1.4727
3031	205	184	Male	0	1.1982
3032	205	184	Male	0	1.2251
3033	205	184	Male	0	1.2546
3034	205	184	Male	0	1.2841
3035	205	184	Male	0	1.3804
3036	205	184	Male	0	1.3912
3037	205	184	Male	0	1.4053
3038	205	184	Male	0	1.4426
3039	206	184	Female	6	1.2068
3040	206	184	Female	6	1.3642
3041	206	184	Female	6	1.4147
3042	206	184	Female	6	1.4632
3043	206	184	Female	6	1.4681
3044	206	184	Female	7	1.3886
3045	206	184	Female	7	1.5134
3046	206	184	Male	0	1.3838
3047	206	184	Male	0	1.3953
3048	206	184	Male	0	1.4025
3049	206	184	Male	0	1.4151
3050	206	184	Male	0	1.4246
3051	206	184	Male	0	1.5080
3052	207	184	Female	6	1.3648
3053	207	184	Male	0	1.3875
3054	207	184	Male	0	1.5553
3055	207	184	Male	0	1.5570
3056	207	184	Male	0	1.5744
3057	208	184	Male	0	1.4417
3058	210	184	Male	0	1.3227
3059	210	184	Male	0	1.3256

3060	210	184	Male	0	1.3722
3061	210	184	Male	0	1.4431
3062	211	184	Female	6	1.3427
3063	211	184	Female	6	1.4843
3064	211	184	Female	7	1.3020
3065	211	184	Female	7	1.3284
3066	211	184	Female	7	1.3548
3067	211	184	Female	7	1.3874
3068	211	184	Male	0	1.3518
3069	211	184	Male	0	1.4287
3070	212	184	Male	0	1.2709
3071	212	184	Male	0	1.3738
3072	213	184	Female	6	1.2789
3073	213	184	Female	8	1.4094
3074	215	184	Female	8	1.4263
3075	215	184	Female	8	1.4734
3076	215	184	Female	9	1.4828
3077	215	184	Male	0	1.3321
3078	215	184	Male	0	1.3345
3079	215	184	Male	0	1.5286
3080	215	184	Male	0	1.5852
3081	217	184	Male	0	1.3350
3082	217	184	Male	0	1.4409
3083	218	184	Female	6	1.2637
3084	218	184	Male	0	1.2289
3085	218	184	Male	0	1.2472
3086	218	184	Male	0	1.3897
3087	219	184	Female	9	1.3501
3088	219	184	Male	0	1.3927
3089	219	184	Male	0	1.4041
3090	220	184	Female	6	1.2348
3091	220	184	Male	0	1.3298
3092	220	184	Male	0	1.3608
3093	222	184	Female	8	1.3357
3094	223	184	Female	7	1.3692
3095	223	184	Female	7	1.4700
3096	223	184	Female	8	1.3335
3097	223	184	Female	8	1.4494
3098	223	184	Male	0	1.1688
3099	223	184	Male	0	1.2010
3100	223	184	Male	0	1.2469
3101	223	184	Male	0	1.2678
3102	223	184	Male	0	1.2810
3103	223	184	Male	0	1.3744

3104	223	184	Male	0	1.3891
3105	223	184	Male	0	1.4060
3106	224	184	Male	0	1.2021
3107	224	184	Male	0	1.2725
3108	224	184	Male	0	1.2797
3109	224	184	Male	0	1.3913
3110	226	184	Female	6	1.0546
3111	226	184	Female	6	1.4617
3112	226	184	Female	8	1.0881
3113	226	184	Female	8	1.1683
3114	226	184	Female	8	1.2790
3115	226	184	Female	8	1.5073
3116	226	184	Female	9	1.1350
3117	226	184	Female	9	1.4326
3118	226	184	Male	0	1.1566
3119	226	184	Male	0	1.2195
3120	226	184	Male	0	1.2907
3121	226	184	Male	0	1.3688
3122	226	184	Male	0	1.3742
3123	226	184	Male	0	1.4127
3124	227	184	Female	6	1.1735
3125	227	184	Female	6	1.3192
3126	227	184	Female	8	1.0192
3127	227	184	Female	8	1.4132
3128	227	184	Male	0	1.1389
3129	227	184	Male	0	1.1532
3130	227	184	Male	0	1.1811
3131	227	184	Male	0	1.2187
3132	227	184	Male	0	1.3177
3133	1	185	Male	0	1.5691
3134	3	185	Female	9	1.1197
3135	3	185	Male	0	1.1826
3136	3	185	Male	0	1.2249
3137	4	185	Female	6	1.1683
3138	4	185	Female	6	1.4543
3139	4	185	Female	6	1.5010
3140	4	185	Female	7	1.4824
3141	4	185	Male	0	1.2971
3142	5	185	Female	8	1.2569
3143	5	185	Male	0	1.3313
3144	7	185	Female	6	1.2099
3145	7	185	Male	0	1.1722
3146	7	185	Male		1.2883
3147	8	185	Female	7	1.2337

3148	8	185	Female	7	1.2497
3149	8	185	Female	8	1.1798
3150	11	185	Female	8	1.3742
3151	12	185	Female	6	1.4338
3152	13	185	Female	8	1.2481
3153	13	185	Female	8	1.3504
3154	13	185	Female	8	1.3540
3155	13	185	Female	8	1.3601
3156	13	185	Female	9	1.3308
3157	13	185	Male	0	1.3471
3158	15	185	Female	6	1.1889
3159	15	185	Female	6	1.4280
3160	15	185	Male	0	1.2885
3161	20	185	Male	0	1.2711
3162	20	185	Male	0	1.2935
3163	20	185	Male	0	1.3793
3164	21	185	Female	6	1.2756
3165	21	185	Female	6	1.3414
3166	21	185	Female	6	1.3745
3167	21	185	Female	7	1.3175
3168	21	185	Female	8	1.3118
3169	26	185	Female	6	1.3592
3170	26	185	Female	6	1.3995
3171	26	185	Female	9	1.3846
3172	26	185	Male	0	1.3485
3173	26	185	Male	0	1.4391
3174	29	185	Female	6	1.3985
3175	29	185	Male	0	1.2673
3176	29	185	Male	0	1.3259
3177	32	185	Female	7	1.3025
3178	32	185	Male	0	1.2409
3179	32	185	Male	0	1.3058
3180	37	185	Female	7	1.2174
3181	37	185	Female	8	1.3893
3182	37	185	Female	9	1.2701
3183	37	185	Male	0	1.0176
3184	37	185	Male	0	1.3559
3185	41	185	Male	0	1.0441
3186	41	185	Male	0	1.1233
3187	42	185	Female	6	1.1264
3188	43	185	Female	6	1.3242
3189	43	185	Male	0	1.2832
3190	43	185	Male	0	1.3079
3191	50	185	Female	6	1.2795

3192	50	185	Female	6	1.3478
3193	50	185	Male	0	1.3356
3194	50	185	Male	0	1.3363
3195	55	185	Female	7	1.0911
3196	55	185	Male	0	1.3332
3197	60	185	Female	6	2.2266
3198	60	185	Female	8	1.6588
3199	60	185	Female	9	1.5762
3200	60	185	Female	9	1.7373
3201	60	185	Female	10	1.5555
3202	60	185	Female	10	1.6776
3203	60	185	Male	0	1.5474
3204	60	185	Male	0	1.5691
3205	60	185	Male	0	1.6003
3206	60	185	Male	0	1.7727
3207	62	185	Female	6	1.2913
3208	62	185	Female	7	1.2281
3209	62	185	Male	0	1.2359
3210	62	185	Male	0	1.2622
3211	63	185	Male	0	0.9464
3212	63	185	Male	0	1.2371
3213	64	185	Female	6	1.3767
3214	64	185	Female	9	1.3665
3215	64	185	Male	0	1.3908
3216	64	185	Male	0	1.4162
3217	66	185	Female	6	1.2597
3218	66	185	Female	8	1.2664
3219	66	185	Male	0	1.0820
3220	66	185	Male	0	1.0967
3221	74	185	Female	6	1.2260
3222	74	185	Female	7	1.3096
3223	74	185	Female	7	1.3496
3224	74	185	Female	8	1.3611
3225	74	185	Female	9	1.3310
3226	74	185	Male	0	1.1410
3227	75	185	Female	7	1.3271
3228	75	185	Female	8	1.3353
3229	75	185	Male	0	1.2598
3230	78	185	Female	6	1.1603
3231	78	185	Female	6	1.2905
3232	78	185	Female	9	1.4020
3233	78	185	Male	0	1.3208
3234	86	185	Female	7	1.2861
3235	86	185	Female	7	1.2876

3236	87	185	Female	6	1.1953
3237	87	185	Female	6	1.3732
3238	87	185	Female	6	1.4506
3239	87	185	Female	7	1.3766
3240	87	185	Male	0	1.3221
3241	87	185	Male	0	1.4433
3242	88	185	Female	7	1.3716
3243	88	185	Male	0	1.2717
3244	88	185	Male	0	1.3412
3245	90	185	Female	6	1.3456
3246	90	185	Female	6	1.3596
3247	90	185	Female	6	1.4046
3248	90	185	Female	8	1.4173
3249	90	185	Male	0	1.2119
3250	90	185	Male	0	1.4219
3251	91	185	Female	6	1.3817
3252	91	185	Female	6	1.4446
3253	91	185	Female	7	1.4671
3254	91	185	Male	0	1.4361
3255	96	185	Female	6	1.4467
3256	96	185	Female	6	1.4530
3257	97	185	Female	6	1.0431
3258	97	185	Female	7	1.2531
3259	97	185	Male	0	1.0613
3260	97	185	Male	0	1.2215
3261	97	185	Male	0	1.2274
3262	102	185	Female	6	1.5611
3263	102	185	Male	0	1.4076
3264	102	185	Male	0	1.4363
3265	102	185	Male	0	1.5287
3266	102	185	Male	0	1.6286
3267	106	185	Female	7	1.4276
3268	106	185	Female	10	1.2232
3269	106	185	Male	0	1.3838
3270	106	185	Male	0	1.3953
3271	106	185	Male	0	1.4114
3272	200	185	Female	7	1.4142
3273	200	185	Female	7	1.4677
3274	200	185	Female	8	1.4354
3275	200	185	Female	8	1.5006
3276	200	185	Male	0	1.3093
3277	200	185	Male	0	1.3376
3278	200	185	Male	0	1.4331
3279	201	185	Female	6	1.2067

3280	201	185	Female	6	1.2068
3281	201	185	Female	6	1.2117
3282	204	185	Female	6	1.4161
3283	204	185	Female	7	1.3368
3284	204	185	Female	8	1.4324
3285	204	185	Male	0	1.0806
3286	205	185	Female	6	1.2553
3287	205	185	Female	6	1.2651
3288	205	185	Female	6	1.4258
3289	205	185	Male	0	1.4516
3290	206	185	Female	6	1.3171
3291	206	185	Female	6	1.4674
3292	206	185	Female	6	1.4881
3293	206	185	Male	0	1.4987
3294	206	185	Male	0	1.5165
3295	207	185	Female	6	1.4373
3296	207	185	Male	0	1.4883
3297	208	185	Female	6	1.2642
3298	208	185	Female	6	1.4151
3299	208	185	Male	0	1.5472
3300	209	185	Female	6	1.5046
3301	210	185	Female	6	1.3496
3302	210	185	Female	6	1.3609
3303	210	185	Male	0	1.2983
3304	210	185	Male	0	1.4066
3305	211	185	Female	6	1.2330
3306	211	185	Female	6	1.4495
3307	211	185	Male	0	1.3557
3308	212	185	Male	0	1.2587
3309	213	185	Female	6	1.1553
3310	213	185	Female	8	1.3906
3311	213	185	Male	0	1.2468
3312	213	185	Male	0	1.2765
3313	213	185	Male	0	1.3038
3314	213	185	Male	0	1.3626
3315	213	185	Male	0	1.4399
3316	215	185	Male	0	1.6009
3317	217	185	Male	0	1.0735
3318	218	185	Female	6	1.3792
3319	218	185	Male	0	1.3687
3320	219	185	Female	7	1.1312
3321	220	185	Male	0	1.3012
3322	220	185	Male	0	1.3016
3323	220	185	Male	0	1.3507

3324	222	185	Male	0	1.4159
3325	223	185	Female	6	1.3278
3326	223	185	Male	0	1.1887
3327	223	185	Male	0	1.1910
3328	223	185	Male	0	1.2655
3329	223	185	Male	0	1.3150
3330	224	185	Female	6	1.1285
3331	224	185	Female	8	1.3227
3332	224	185	Male	0	1.3538
3333	224	185	Male	0	1.3584
3334	224	185	Male	0	1.3998
3335	226	185	Female	6	1.1238
3336	226	185	Female	7	1.1569
3337	226	185	Female	9	1.3785
3338	226	185	Male	0	1.1523
3339	226	185	Male	0	1.2386
3340	227	185	Female	6	1.4662
3341	227	185	Female	7	1.1766
3342	227	185	Male	0	1.2346
3343	227	185	Male	0	1.3523
3344	228	185	Male	0	1.2466
3345	12	187	Female	6	1.1947
3346	13	187	Female	6	1.3954
3347	14	187	Female	7	1.1521
3348	14	187	Male	0	0.9222
3349	14	187	Male	0	1.1101
3350	15	187	Male	0	1.0766
3351	16	187	Female	6	1.2373
3352	16	187	Female	8	1.2392
3353	17	187	Female	6	1.1178
3354	18	187	Female	6	1.2571
3355	19	187	Female	6	1.3445
3356	19	187	Female	6	1.4069
3357	19	187	Female	7	1.3209
3358	19	187	Female	7	1.4378
3359	19	187	Male	0	1.2492
3360	20	187	Female	8	1.3358
3361	20	187	Male	0	1.2657
3362	20	187	Male	0	1.2736
3363	20	187	Male	0	1.3076
3364	20	187	Male	0	1.3312
3365	24	187	Female	6	1.2640
3366	24	187	Female	7	1.1876
3367	24	187	Female	8	1.3147

3368	26	187	Female	8	1.3621
3369	26	187	Male	0	1.3168
3370	27	187	Female	7	1.2998
3371	29	187	Female	6	1.2129
3372	29	187	Female	6	1.3487
3373	29	187	Male	0	1.1116
3374	31	187	Female	6	1.2843
3375	31	187	Female	7	1.2741
3376	31	187	Female	9	1.2557
3377	31	187	Male	0	1.3092
3378	32	187	Female	9	1.4269
3379	32	187	Male	0	1.1717
3380	32	187	Male	0	1.3019
3381	32	187	Male	0	1.3294
3382	32	187	Male	0	1.3854
3383	32	187	Male	0	1.3879
3384	34	187	Male	0	1.3272
3385	34	187	Male	0	1.3349
3386	34	187	Male	0	1.3643
3387	34	187	Male	0	1.4134
3388	35	187	Male	0	1.0165
3389	35	187	Male	0	1.1384
3390	35	187	Male	0	1.1946
3391	35	187	Male	0	1.2453
3392	35	187	Male	0	1.4557
3393	36	187	Female	6	1.2371
3394	36	187	Male	0	1.3473
3395	36	187	Male	0	1.3853
3396	36	187	Male	0	1.4134
3397	37	187	Female	6	1.2260
3398	37	187	Male	0	1.1534
3399	37	187	Male	0	1.3364
3400	37	187	Male	0	1.3568
3401	38	187	Female	6	1.2527
3402	42	187	Female	8	1.0274
3403	42	187	Male	0	1.2621
3404	43	187	Female	7	1.2689
3405	43	187	Male	0	1.2605
3406	43	187	Male	0	1.3902
3407	50	187	Female	6	1.3982
3408	51	187	Female	7	1.2934
3409	51	187	Male	0	1.2061
3410	53	187	Female	6	1.2709
3411	56	187	Male	0	1.2124

3412	56	187	Male	0	1.2434
3413	57	187	Female	6	1.2221
3414	57	187	Female	6	1.2832
3415	57	187	Male	0	1.1793
3416	59	187	Female	6	1.0669
3417	59	187	Female	8	1.1331
3418	60	187	Female	7	1.4374
3419	60	187	Female	7	1.8177
3420	60	187	Female	7	2.0008
3421	60	187	Female	8	1.5497
3422	60	187	Female	9	1.5837
3423	60	187	Female	9	1.7924
3424	60	187	Female	9	1.8163
3425	60	187	Female	9	1.8813
3426	60	187	Male	0	1.9730
3427	61	187	Female	6	1.3506
3428	61	187	Female	6	1.3554
3429	61	187	Female	8	1.3314
3430	61	187	Female	8	1.3474
3431	61	187	Male	0	1.3072
3432	63	187	Female	6	1.1990
3433	63	187	Female	6	1.2904
3434	63	187	Male	0	1.2777
3435	64	187	Female	9	1.4159
3436	65	187	Male	0	1.2153
3437	65	187	Male	0	1.2436
3438	67	187	Female	6	1.3474
3439	67	187	Female	7	1.3328
3440	67	187	Male	0	1.1559
3441	67	187	Male	0	1.3523
3442	69	187	Female	7	1.1972
3443	69	187	Male	0	1.1454
3444	69	187	Male	0	1.1736
3445	69	187	Male	0	1.1818
3446	69	187	Male	0	1.2305
3447	69	187	Male	0	1.2784
3448	70	187	Female	6	1.2893
3449	70	187	Female	6	1.3603
3450	70	187	Male	0	1.2956
3451	70	187	Male	0	1.3111
3452	70	187	Male	0	1.3390
3453	71	187	Female	6	1.2126
3454	71	187	Male	0	1.2333
3455	71	187	Male	0	1.2384

3456	71	187	Male	0	1.2766
3457	74	187	Female	6	1.3315
3458	74	187	Male	0	1.2544
3459	74	187	Male	0	1.3585
3460	74	187	Male	0	1.4506
3461	75	187	Female	8	1.3331
3462	75	187	Male	0	1.2574
3463	75	187	Male	0	1.2699
3464	75	187	Male	0	1.2998
3465	75	187	Male	0	1.3126
3466	76	187	Female	6	1.1734
3467	76	187	Female	6	1.2396
3468	76	187	Female	6	1.2665
3469	76	187	Female	6	1.4174
3470	76	187	Female	7	1.1673
3471	76	187	Female	7	1.2234
3472	76	187	Male	0	0.9573
3473	78	187	Female	8	1.3583
3474	79	187	Female	6	1.1334
3475	81	187	Female	9	1.3286
3476	81	187	Male	0	1.2213
3477	81	187	Male	0	1.3718
3478	83	187	Male	0	1.2106
3479	84	187	Female	8	1.2670
3480	84	187	Male	0	1.1719
3481	84	187	Male	0	1.2494
3482	86	187	Female	6	1.3328
3483	86	187	Female	7	1.2076
3484	86	187	Female	8	1.3233
3485	90	187	Female	6	1.3276
3486	90	187	Female	6	1.3467
3487	91	187	Female	6	1.3953
3488	91	187	Female	6	1.4418
3489	91	187	Female	6	1.4522
3490	91	187	Female	7	1.4796
3491	91	187	Male	0	1.4330
3492	91	187	Male	0	1.4848
3493	97	187	Female	6	1.3183
3494	97	187	Female	7	1.1890
3495	98	187	Female	6	1.2851
3496	98	187	Female	7	1.2122
3497	98	187	Female	7	1.2396
3498	98	187	Female	7	1.2467
3499	98	187	Male	0	1.1867

3500	98	187	Male	0	1.2342
3501	99	187	Female	8	1.2103
3502	99	187	Male	0	1.2758
3503	100	187	Male	0	1.3332
3504	101	187	Female	9	1.4413
3505	102	187	Female	6	1.3872
3506	102	187	Female	6	1.4166
3507	102	187	Female	6	1.4316
3508	102	187	Female	6	1.4371
3509	102	187	Female	6	1.4532
3510	102	187	Female	6	1.4830
3511	102	187	Female	6	1.5025
3512	102	187	Male	0	1.4071
3513	102	187	Male	0	1.4426
3514	102	187	Male	0	1.4596
3515	102	187	Male	0	1.5118
3516	105	187	Female	6	1.2077
3517	105	187	Female	8	1.3554
3518	105	187	Male	0	1.2254
3519	106	187	Female	9	1.3488
3520	106	187	Male	0	1.3064
3521	106	187	Male	0	1.3490
3522	107	187	Male	0	1.2537
3523	200	187	Female	6	1.3022
3524	200	187	Female	6	1.4030
3525	200	187	Female	7	1.4486
3526	200	187	Female	7	1.4656
3527	200	187	Female	8	1.4072
3528	200	187	Male	0	1.2569
3529	200	187	Male	0	1.3696
3530	200	187	Male	0	1.5858
3531	200	187	Male	0	1.5924
3532	200	187	Male	0	1.6185
3533	204	187	Female	6	1.4017
3534	204	187	Female	6	1.4471
3535	204	187	Female	8	1.4828
3536	204	187	Male	0	1.3409
3537	204	187	Male	0	1.4616
3538	204	187	Male	0	1.4682
3539	205	187	Female	6	1.0695
3540	205	187	Female	6	1.4066
3541	205	187	Female	7	1.3805
3542	207	187	Female	8	1.3234
3543	207	187	Female	8	1.4272

3544	207	187	Male	0	1.4284
3545	207	187	Male	0	1.4299
3546	207	187	Male	0	1.4930
3547	208	187	Male	0	1.5775
3548	209	187	Male	0	1.4074
3549	210	187	Female	8	1.4763
3550	210	187	Male	0	1.2983
3551	210	187	Male	0	1.3318
3552	210	187	Male	0	1.3989
3553	210	187	Male	0	1.4143
3554	210	187	Male	0	1.4692
3555	211	187	Female	7	1.4601
3556	211	187	Female	8	1.3866
3557	212	187	Female	6	1.1873
3558	212	187	Female	6	1.3759
3559	212	187	Female	6	1.5348
3560	212	187	Female	7	1.2295
3561	212	187	Female	7	1.3221
3562	212	187	Female	8	1.4837
3563	212	187	Male	0	1.2128
3564	212	187	Male	0	1.2687
3565	212	187	Male	0	1.3018
3566	212	187	Male	0	1.3111
3567	212	187	Male	0	1.3382
3568	212	187	Male	0	1.3544
3569	212	187	Male	0	1.3887
3570	212	187	Male	0	1.3966
3571	213	187	Female	6	1.2947
3572	213	187	Female	6	1.3647
3573	213	187	Female	7	1.3218
3574	213	187	Male	0	1.1796
3575	215	187	Female	7	1.3938
3576	215	187	Female	9	1.3938
3577	215	187	Female	9	1.4596
3578	215	187	Male	0	1.3739
3579	215	187	Male	0	1.4409
3580	215	187	Male	0	1.4478
3581	217	187	Female	9	1.3446
3582	217	187	Female	10	1.2312
3583	217	187	Male	0	1.0592
3584	217	187	Male	0	1.1290
3585	217	187	Male	0	1.3688
3586	218	187	Female	6	1.2776
3587	218	187	Female	6	1.3264

3588	218	187	Female	6	1.4357
3589	218	187	Female	7	1.3139
3590	218	187	Male	0	1.2148
3591	218	187	Male	0	1.3271
3592	219	187	Female	6	1.1429
3593	219	187	Male	0	1.3004
3594	219	187	Male	0	1.3596
3595	222	187	Male	0	1.1806
3596	222	187	Male	0	1.2957
3597	222	187	Male	0	1.3614
3598	223	187	Female	6	1.2464
3599	223	187	Female	6	1.2668
3600	223	187	Female	6	1.5291
3601	224	187	Female	6	1.2005
3602	224	187	Female	6	1.2783
3603	224	187	Male	0	1.3259
3604	224	187	Male	0	1.3743
3605	226	187	Female	6	1.1472
3606	226	187	Female	6	1.4895
3607	226	187	Female	7	1.4156
3608	226	187	Female	7	1.4989
3609	226	187	Female	8	1.4604
3610	226	187	Female	9	1.1006
3611	226	187	Female	9	1.3854
3612	226	187	Male	0	1.1434
3613	226	187	Male	0	1.1639
3614	226	187	Male	0	1.3272
3615	226	187	Male	0	1.3730
3616	227	187	Female	6	1.2519
3617	227	187	Female	7	1.0963
3618	227	187	Female	7	1.1411
3619	227	187	Female	7	1.3671
3620	227	187	Female	7	1.4123
3621	227	187	Male	0	1.1794
3622	227	187	Male	0	1.2203
3623	227	187	Male	0	1.3519
3624	5	188	Female	6	1.1581
3625	8	188	Male	0	1.3671
3626	11	188	Female	6	1.2582
3627	12	188	Female	6	1.2543
3628	12	188	Female	6	1.3497
3629	13	188	Male	0	1.2094
3630	13	188	Male	0	1.3288
3631	14	188	Male	0	1.3399

3632	17	188	Female	6	1.3350
3633	17	188	Male	0	1.1376
3634	18	188	Male	0	1.2723
3635	20	188	Female	7	1.3380
3636	26	188	Male	0	1.3995
3637	26	188	Male	0	1.4202
3638	29	188	Female	7	1.3637
3639	31	188	Female	8	1.3171
3640	31	188	Female	9	1.3447
3641	31	188	Male	0	1.3641
3642	32	188	Female	7	1.3613
3643	32	188	Female	8	1.3041
3644	32	188	Male	0	1.3335
3645	35	188	Female	9	1.0184
3646	35	188	Male	0	1.1140
3647	35	188	Male	0	1.2966
3648	35	188	Male	0	1.4310
3649	36	188	Female	7	1.2562
3650	36	188	Male	0	1.0873
3651	37	188	Female	9	1.3742
3652	37	188	Male	0	1.3667
3653	38	188	Female	6	1.2602
3654	38	188	Female	7	1.2437
3655	38	188	Male	0	1.1501
3656	38	188	Male	0	1.2350
3657	43	188	Female	8	1.3359
3658	43	188	Male	0	0.9799
3659	49	188	Male	0	1.1341
3660	51	188	Female	6	1.2338
3661	53	188	Female	7	1.2346
3662	53	188	Female	10	1.2177
3663	53	188	Male	0	1.2783
3664	54	188	Female	6	1.1195
3665	54	188	Female	6	1.1877
3666	54	188	Female	8	1.1667
3667	55	188	Female	7	1.2702
3668	56	188	Male	0	1.1713
3669	62	188	Female	7	1.3269
3670	63	188	Male	0	1.1066
3671	64	188	Female	6	1.3176
3672	69	188	Female	7	1.2033
3673	74	188	Female	6	1.2093
3674	74	188	Female	6	1.3378
3675	74	188	Female	9	1.3903

3676	75	188	Female	9	1.3086
3677	76	188	Female	6	1.2595
3678	78	188	Female	7	1.3197
3679	78	188	Female	7	1.3466
3680	78	188	Male	0	1.1681
3681	78	188	Male	0	1.4737
3682	79	188	Male	0	1.1219
3683	81	188	Female	8	1.3414
3684	81	188	Male	0	1.3646
3685	86	188	Female	6	1.2242
3686	86	188	Female	8	1.2772
3687	88	188	Male	0	0.9971
3688	88	188	Male	0	1.0828
3689	97	188	Female	6	1.1909
3690	97	188	Male	0	1.2287
3691	98	188	Male	0	1.0790
3692	99	188	Female	8	1.2507
3693	99	188	Male	0	1.0307
3694	101	188	Female	8	1.0570
3695	102	188	Male	0	1.4599
3696	105	188	Female	7	1.1519
3697	106	188	Male	0	1.4265
3698	200	188	Female	6	1.5637
3699	200	188	Female	7	1.6102
3700	201	188	Male	0	1.1811
3701	204	188	Female	6	1.2705
3702	204	188	Female	6	1.3884
3703	204	188	Female	7	1.4846
3704	204	188	Female	7	1.4846
3705	204	188	Female	7	1.4883
3706	204	188	Female	8	1.3383
3707	204	188	Male	0	1.0638
3708	204	188	Male	0	1.0765
3709	204	188	Male	0	1.1020
3710	204	188	Male	0	1.2193
3711	204	188	Male	0	1.3050
3712	204	188	Male	0	1.4849
3713	204	188	Male	0	1.5405
3714	205	188	Female	6	1.1293
3715	207	188	Female	7	1.2493
3716	207	188	Female	7	1.4105
3717	207	188	Female	8	1.2439
3718	208	188	Female	8	1.3594
3719	208	188	Male	0	1.4227

3720	211	188	Female	6	1.4853
3721	212	188	Female	6	1.3563
3722	212	188	Female	7	1.4365
3723	212	188	Female	8	1.4166
3724	212	188	Male	0	1.3829
3725	213	188	Female	6	1.1103
3726	213	188	Female	6	1.4018
3727	213	188	Male	0	1.2073
3728	215	188	Female	7	1.3304
3729	215	188	Female	9	1.3668
3730	215	188	Male	0	1.3781
3731	218	188	Female	6	1.2608
3732	218	188	Female	6	1.2776
3733	218	188	Female	6	1.3503
3734	218	188	Female	6	1.8170
3735	218	188	Female	7	1.1891
3736	218	188	Female	7	1.3884
3737	219	188	Female	6	1.2585
3738	219	188	Female	6	1.3265
3739	219	188	Female	6	1.4418
3740	219	188	Female	6	1.5408
3741	219	188	Female	10	1.5653
3742	219	188	Male	0	1.1261
3743	219	188	Male	0	1.2628
3744	219	188	Male	0	1.3242
3745	219	188	Male	0	1.3860
3746	220	188	Male	0	1.2937
3747	222	188	Female	8	1.2834
3748	222	188	Female	9	1.2979
3749	222	188	Male	0	1.1925
3750	222	188	Male	0	1.3206
3751	222	188	Male	0	1.3804
3752	224	188	Female	6	1.3635
3753	224	188	Male	0	1.2918
3754	226	188	Male	0	1.2036
3755	228	188	Female	6	1.1505
3756	1	191	Female	7	1.2037
3757	1	191	Female	7	1.3523
3758	1	191	Female	8	1.0149
3759	1	191	Female	8	1.3462
3760	1	191	Female	8	1.4445
3761	1	191	Female	8	1.4701
3762	1	191	Female	9	1.4442
3763	1	191	Male	0	1.1235

3764	1	191	Male	0	1.4509
3765	3	191	Female	7	1.1254
3766	3	191	Male	0	1.3467
3767	8	191	Female	7	1.3208
3768	8	191	Female	8	1.1719
3769	8	191	Male	0	1.1894
3770	11	191	Female	6	1.3303
3771	11	191	Male	0	0.9021
3772	11	191	Male	0	0.9884
3773	11	191	Male	0	1.1865
3774	11	191	Male	0	1.4044
3775	12	191	Female	6	1.2468
3776	12	191	Female	6	1.2777
3777	12	191	Male	0	1.2525
3778	12	191	Male	0	1.3165
3779	12	191	Male	0	1.4007
3780	13	191	Female	6	1.3204
3781	13	191	Female	6	1.3713
3782	13	191	Female	8	1.3622
3783	13	191	Male	0	1.3619
3784	15	191	Female	6	1.3363
3785	15	191	Female	6	1.4824
3786	18	191	Male	0	1.1433
3787	19	191	Female	6	1.3217
3788	19	191	Male	0	1.1732
3789	20	191	Female	6	1.2338
3790	20	191	Male	0	1.2448
3791	22	191	Female	6	1.3539
3792	22	191	Female	7	1.3897
3793	22	191	Female	8	1.3474
3794	24	191	Male	0	1.2978
3795	24	191	Male	0	1.3604
3796	24	191	Male	0	1.4917
3797	26	191	Female	6	1.2796
3798	27	191	Female	6	1.2036
3799	27	191	Female	7	1.2914
3800	29	191	Male	0	1.1675
3801	31	191	Female	6	1.1629
3802	31	191	Male	0	1.2769
3803	31	191	Male	0	1.3654
3804	32	191	Male	0	1.1399
3805	32	191	Male	0	1.2783
3806	34	191	Female	8	1.2462
3807	34	191	Female	9	1.2815

3808	34	191	Male	0	1.3653
3809	35	191	Male	0	1.3656
3810	37	191	Female	6	1.2674
3811	37	191	Male	0	1.2974
3812	37	191	Male	0	1.3234
3813	37	191	Male	0	1.3459
3814	38	191	Male	0	1.0843
3815	41	191	Female	7	1.0421
3816	41	191	Male	0	1.1672
3817	43	191	Female	7	1.3131
3818	43	191	Male	0	1.2503
3819	50	191	Female	6	1.2929
3820	50	191	Female	6	1.3231
3821	50	191	Male	0	1.3212
3822	50	191	Male	0	1.3315
3823	53	191	Female	6	1.1584
3824	53	191	Female	6	1.1983
3825	53	191	Male	0	1.3112
3826	59	191	Female	6	1.2321
3827	59	191	Male	0	0.9725
3828	60	191	Female	6	1.5638
3829	60	191	Female	6	1.6172
3830	60	191	Female	7	1.6976
3831	60	191	Female	7	1.7393
3832	60	191	Female	8	1.6059
3833	60	191	Female	8	2.0857
3834	60	191	Female	9	1.7291
3835	60	191	Male	0	1.5702
3836	60	191	Male	0	1.7610
3837	60	191	Male	0	1.9472
3838	62	191	Female	6	1.2328
3839	62	191	Female	6	1.2662
3840	62	191	Female	6	1.2761
3841	64	191	Female	6	1.3999
3842	65	191	Female	6	1.1089
3843	65	191	Male	0	1.2392
3844	67	191	Female	6	1.1026
3845	67	191	Female	7	1.4146
3846	67	191	Male	0	1.2076
3847	70	191	Female	7	1.3766
3848	73	191	Female	6	1.1779
3849	73	191	Female	6	1.2672
3850	73	191	Female	6	1.3084
3851	73	191	Female	6	1.3257

3852	73	191	Male	0	1.1309
3853	73	191	Male	0	1.2428
3854	76	191	Female	6	1.3562
3855	76	191	Male	0	1.0709
3856	87	191	Female	6	1.2530
3857	87	191	Male	0	1.1631
3858	87	191	Male	0	1.3813
3859	87	191	Male	0	1.4239
3860	90	191	Male	0	1.3577
3861	91	191	Female	6	1.4311
3862	91	191	Male	0	1.3947
3863	97	191	Female	6	1.2281
3864	97	191	Male	0	1.2638
3865	97	191	Male	0	1.2653
3866	98	191	Female	6	1.3011
3867	98	191	Female	7	1.2683
3868	99	191	Female	8	1.0982
3869	100	191	Female	6	1.1431
3870	102	191	Female	7	1.6769
3871	102	191	Male	0	1.5001
3872	102	191	Male	0	1.5391
3873	105	191	Female	6	1.1672
3874	105	191	Female	8	1.2093
3875	105	191	Male	0	1.2192
3876	106	191	Male	0	1.3763
3877	107	191	Male	0	1.2300
3878	200	191	Male	0	1.3965
3879	201	191	Male	0	1.2176
3880	204	191	Female	6	1.2901
3881	204	191	Female	7	1.5222
3882	204	191	Male	0	1.1792
3883	204	191	Male	0	1.4744
3884	204	191	Male	0	1.4750
3885	204	191	Male	0	1.4961
3886	204	191	Male	0	1.5337
3887	206	191	Female	8	1.4586
3888	206	191	Male	0	1.5475
3889	207	191	Female	6	1.3217
3890	207	191	Female	6	1.3898
3891	207	191	Female	6	1.4327
3892	207	191	Female	7	1.3945
3893	207	191	Female	7	1.4388
3894	207	191	Male	0	1.5712
3895	208	191	Female	6	1.2511

3896	208	191	Female	6	1.4445
3897	210	191	Female	7	1.3184
3898	210	191	Male	0	1.2111
3899	210	191	Male	0	1.2319
3900	210	191	Male	0	1.2486
3901	210	191	Male	0	1.3368
3902	211	191	Female	6	1.4170
3903	211	191	Female	7	1.3558
3904	211	191	Female	7	1.3943
3905	215	191	Female	6	1.4293
3906	215	191	Female	7	1.3892
3907	215	191	Female	8	1.4406
3908	215	191	Female	8	1.4801
3909	215	191	Male	0	1.4418
3910	215	191	Male	0	1.4477
3911	215	191	Male	0	1.5459
3912	217	191	Female	7	1.3934
3913	217	191	Female	8	1.3844
3914	217	191	Female	9	1.4013
3915	218	191	Female	7	1.2727
3916	218	191	Female	7	1.3522
3917	220	191	Female	6	1.2222
3918	220	191	Female	7	1.2963
3919	220	191	Female	8	1.2815
3920	220	191	Male	0	1.2737
3921	220	191	Male	0	1.4059
3922	222	191	Female	6	1.5768
3923	222	191	Female	7	1.1653
3924	222	191	Male	0	1.3915
3925	223	191	Female	6	1.1762
3926	223	191	Female	6	1.2751
3927	223	191	Female	7	1.5764
3928	223	191	Female	8	1.2285
3929	224	191	Female	6	1.4663
3930	224	191	Female	7	1.1346
3931	224	191	Female	7	1.2261
3932	224	191	Female	7	1.5131
3933	224	191	Female	7	1.5755
3934	224	191	Female	8	1.4053
3935	226	191	Female	8	1.4657
3936	226	191	Female	9	1.0496
3937	226	191	Male	0	1.2376
3938	226	191	Male	0	1.3886
3939	227	191	Female	7	1.1151

3940	227	191	Female	8	1.0251
3941	227	191	Female	8	1.2556
3942	227	191	Female	8	1.3227
3943	227	191	Female	9	1.3863
3944	227	191	Male	0	1.2140
3945	227	191	Male	0	1.2827
3946	227	191	Male	0	1.2870
3947	228	191	Female	7	1.0418
3948	228	191	Female	7	1.3461
3949	228	191	Female	7	1.4518
3950	228	191	Male	0	1.3787
3951	1	192	Female	6	1.3064
3952	1	192	Female	7	1.4161
3953	3	192	Female	7	1.0937
3954	3	192	Male	0	1.1609
3955	3	192	Male	0	1.3221
3956	5	192	Female	6	1.3003
3957	5	192	Female	7	1.3148
3958	5	192	Male	0	1.2314
3959	8	192	Female	7	1.2324
3960	11	192	Female	7	1.2445
3961	12	192	Female	6	1.2679
3962	12	192	Male	0	1.2626
3963	13	192	Female	6	1.2796
3964	15	192	Male	0	1.2972
3965	18	192	Male	0	1.2218
3966	19	192	Female	6	1.2901
3967	19	192	Male	0	1.3030
3968	19	192	Male	0	1.3849
3969	20	192	Female	6	1.3110
3970	20	192	Male	0	1.2682
3971	21	192	Female	7	1.3243
3972	21	192	Male	0	1.2211
3973	21	192	Male	0	1.2238
3974	24	192	Female	6	1.2310
3975	24	192	Female	6	1.4179
3976	26	192	Female	6	1.3799
3977	26	192	Male	0	1.3670
3978	31	192	Male	0	1.2923
3979	34	192	Female	7	1.2787
3980	34	192	Female	7	1.3442
3981	35	192	Female	6	1.2213
3982	35	192	Female	7	1.3256
3983	37	192	Female	6	1.1667

3984	37	192	Female	6	1.3312
3985	38	192	Female	7	1.3987
3986	38	192	Male	0	1.1165
3987	41	192	Male	0	1.0403
3988	41	192	Male	0	1.0647
3989	43	192	Male	0	0.9296
3990	51	192	Female	6	1.2393
3991	53	192	Female	6	1.2393
3992	53	192	Female	6	1.3242
3993	53	192	Male	0	1.1615
3994	53	192	Male	0	1.3163
3995	61	192	Female	7	1.3206
3996	61	192	Male	0	1.2218
3997	64	192	Female	6	1.3709
3998	65	192	Female	6	1.2063
3999	65	192	Female	9	1.3164
4000	65	192	Male	0	1.2323
4001	67	192	Female	6	1.3658
4002	69	192	Female	6	1.1682
4003	69	192	Female	6	1.2577
4004	70	192	Female	6	1.2454
4005	70	192	Male	0	1.2439
4006	70	192	Male	0	1.3481
4007	77	192	Male	0	0.9664
4008	77	192	Male	0	1.1428
4009	78	192	Male	0	1.3019
4010	79	192	Male	0	1.1233
4011	87	192	Male	0	1.2025
4012	87	192	Male	0	1.4006
4013	88	192	Female	7	1.3123
4014	88	192	Male	0	1.0335
4015	88	192	Male	0	1.1201
4016	88	192	Male	0	1.3180
4017	90	192	Female	6	1.4072
4018	90	192	Male	0	1.2740
4019	90	192	Male	0	1.4657
4020	91	192	Male	0	1.2650
4021	91	192	Male	0	1.4413
4022	96	192	Female	8	1.2783
4023	96	192	Male	0	1.1304
4024	96	192	Male	0	1.1326
4025	98	192	Female	6	1.2134
4026	99	192	Female	9	1.2033
4027	99	192	Male	0	1.2847

4028	101	192	Female	7	1.3433
4029	101	192	Male	0	1.2914
4030	101	192	Male	0	1.3810
4031	102	192	Female	7	1.4317
4032	106	192	Female	6	1.2279
4033	106	192	Female	9	1.3347
4034	107	192	Female	7	1.2464
4035	200	192	Male	0	1.3903
4036	200	192	Male	0	1.5891
4037	202	192	Female	6	1.2291
4038	204	192	Female	6	1.4969
4039	205	192	Female	6	1.1130
4040	207	192	Female	6	1.4760
4041	208	192	Male	0	1.4856
4042	210	192	Female	6	1.3355
4043	211	192	Female	6	1.3712
4044	211	192	Female	6	1.3972
4045	212	192	Female	8	1.2599
4046	213	192	Male	0	1.0835
4047	213	192	Male	0	1.2591
4048	215	192	Female	9	1.5106
4049	215	192	Male	0	1.4486
4050	218	192	Female	7	1.4287
4051	218	192	Female	9	1.3688
4052	219	192	Female	8	1.3074
4053	219	192	Male	0	1.2133
4054	219	192	Male	0	1.2912
4055	220	192	Male	0	1.2474
4056	220	192	Male	0	1.3124
4057	220	192	Male	0	1.3558
4058	222	192	Female	7	1.1773
4059	222	192	Female	7	1.3030
4060	222	192	Male	0	1.3930
4061	224	192	Female	6	1.2727
4062	224	192	Female	6	1.3518
4063	224	192	Male	0	1.3671
4064	226	192	Female	6	1.1681
4065	226	192	Female	7	1.3014
4066	226	192	Male	0	1.1935
4067	226	192	Male	0	1.3209
4068	227	192	Female	7	1.2404
4069	227	192	Female	7	1.4689
4070	227	192	Female	7	1.5098
4071	227	192	Female	8	1.1768

4072	227	192	Female	10	1.2601
4073	228	192	Female	7	1.1332
4074	228	192	Female	10	1.4476
4075	228	192	Male	0	1.1776
4076	1	194	Female	6	1.2738
4077	1	194	Female	8	1.3806
4078	1	194	Male	0	1.1093
4079	1	194	Male	0	1.5567
4080	4	194	Female	6	1.4374
4081	5	194	Female	6	1.2648
4082	5	194	Female	6	1.3158
4083	5	194	Male	0	1.3394
4084	5	194	Male	0	1.3867
4085	8	194	Female	6	0.9093
4086	11	194	Male	0	0.9652
4087	11	194	Male	0	1.1577
4088	11	194	Male	0	1.2880
4089	12	194	Female	6	1.4198
4090	12	194	Female	6	1.4223
4091	12	194	Female	9	1.2078
4092	12	194	Male	0	1.2227
4093	12	194	Male	0	1.3921
4094	14	194	Female	6	1.2505
4095	14	194	Male	0	1.3455
4096	15	194	Female	6	1.2036
4097	19	194	Female	8	1.3091
4098	19	194	Male	0	1.2617
4099	20	194	Female	6	1.0418
4100	20	194	Female	6	1.2313
4101	20	194	Male	0	1.1694
4102	20	194	Male	0	1.3267
4103	20	194	Male	0	1.7623
4104	21	194	Female	6	1.2096
4105	21	194	Female	6	1.3194
4106	21	194	Female	6	1.4024
4107	21	194	Male	0	1.2486
4108	22	194	Female	6	1.0231
4109	22	194	Male	0	1.3582
4110	24	194	Female	6	1.3293
4111	26	194	Female	6	1.2834
4112	26	194	Female	6	1.3257
4113	26	194	Male	0	1.2958
4114	26	194	Male	0	1.3709
4115	27	194	Female	7	1.2369

4116	29	194	Female	7	1.4328
4117	29	194	Male	0	1.1231
4118	32	194	Female	6	1.2112
4119	32	194	Male	0	1.2576
4120	32	194	Male	0	1.3137
4121	34	194	Female	5	1.1024
4122	37	194	Female	6	1.3092
4123	37	194	Female	6	1.3202
4124	37	194	Male	0	1.1123
4125	37	194	Male	0	1.1641
4126	37	194	Male	0	1.1998
4127	38	194	Female	6	1.2317
4128	38	194	Female	6	1.3307
4129	38	194	Male	0	1.0902
4130	38	194	Male	0	1.1219
4131	38	194	Male	0	1.1299
4132	38	194	Male	0	1.3102
4133	43	194	Female	7	1.2705
4134	46	194	Male	0	1.2313
4135	46	194	Male	0	1.2327
4136	46	194	Male	0	1.3217
4137	50	194	Female	7	1.3186
4138	50	194	Male	0	1.0039
4139	50	194	Male	0	1.0859
4140	53	194	Male	0	1.1920
4141	53	194	Male	0	1.2118
4142	54	194	Male	0	1.2443
4143	55	194	Female	6	1.2487
4144	60	194	Female	8	1.6297
4145	60	194	Female	8	1.7128
4146	60	194	Female	8	1.8054
4147	61	194	Male	0	0.8784
4148	61	194	Male	0	1.3531
4149	62	194	Male	0	1.0347
4150	63	194	Male	0	1.2614
4151	64	194	Female	6	1.3208
4152	64	194	Female	6	1.3994
4153	67	194	Female	6	1.3134
4154	67	194	Male	0	1.2842
4155	67	194	Male	0	1.3102
4156	67	194	Male	0	1.4181
4157	74	194	Female	6	1.1578
4158	74	194	Female	6	1.1698
4159	74	194	Female	6	1.3434

4160	74	194	Female	7	1.3668
4161	74	194	Female	9	1.3797
4162	74	194	Female	10	1.2701
4163	74	194	Male	0	1.1294
4164	74	194	Male	0	1.2825
4165	74	194	Male	0	1.3921
4166	75	194	Female	7	1.2578
4167	75	194	Female	8	1.2876
4168	75	194	Male	0	1.2351
4169	75	194	Male	0	1.2399
4170	76	194	Female	6	1.1657
4171	76	194	Male	0	1.0792
4172	78	194	Female	6	1.2436
4173	78	194	Female	6	1.3649
4174	78	194	Female	6	1.3753
4175	78	194	Male	0	1.3248
4176	78	194	Male	0	1.3321
4177	78	194	Male	0	1.3433
4178	79	194	Male	0	1.2174
4179	83	194	Male	0	1.2272
4180	86	194	Female	7	1.3071
4181	86	194	Male	0	1.2984
4182	87	194	Female	6	1.1521
4183	87	194	Male	0	1.1379
4184	87	194	Male	0	1.1384
4185	87	194	Male	0	1.2652
4186	90	194	Female	7	1.2941
4187	90	194	Male	0	1.1737
4188	90	194	Male	0	1.2129
4189	90	194	Male	0	1.2163
4190	90	194	Male	0	1.2536
4191	90	194	Male	0	1.3090
4192	90	194	Male	0	1.3385
4193	90	194	Male	0	1.3592
4194	90	194	Male	0	1.5174
4195	91	194	Female	6	1.3363
4196	91	194	Female	6	1.4322
4197	96	194	Male	0	1.2932
4198	97	194	Male	0	1.0918
4199	97	194	Male	0	1.0920
4200	99	194	Female	7	1.1524
4201	102	194	Female	7	1.4611
4202	106	194	Female	6	1.3401
4203	106	194	Female	7	1.4352

4204	107	194	Female	6	1.3335
4205	107	194	Female	6	1.3427
4206	200	194	Female	6	1.0785
4207	200	194	Female	6	1.3122
4208	200	194	Female	6	1.4867
4209	200	194	Female	6	1.5636
4210	200	194	Female	7	1.4603
4211	200	194	Female	8	1.5937
4212	200	194	Male	0	1.3890
4213	205	194	Female	6	1.0785
4214	206	194	Female	6	1.0589
4215	206	194	Female	6	1.1712
4216	206	194	Female	6	1.4361
4217	206	194	Female	7	1.4757
4218	207	194	Female	7	1.4394
4219	210	194	Female	6	1.3804
4220	211	194	Female	8	1.2835
4221	215	194	Female	7	1.4398
4222	215	194	Male	0	1.5583
4223	217	194	Female	8	1.4069
4224	219	194	Female	6	1.1783
4225	219	194	Female	6	1.5727
4226	219	194	Female	7	1.5108
4227	219	194	Male	0	1.1568
4228	219	194	Male	0	1.2421
4229	222	194	Female	6	1.5629
4230	222	194	Female	7	1.4244
4231	222	194	Female	8	1.1962
4232	222	194	Male	0	1.1497
4233	222	194	Male	0	1.2236
4234	223	194	Male	0	1.4067
4235	224	194	Female	6	1.2862
4236	224	194	Female	6	1.5219
4237	224	194	Female	7	1.2726
4238	224	194	Female	8	1.4612
4239	224	194	Male	0	1.1504
4240	224	194	Male	0	1.2307
4241	224	194	Male	0	1.3414
4242	224	194	Male	0	1.4146
4243	224	194	Male	0	1.4166
4244	226	194	Female	6	1.4505
4245	226	194	Female	7	1.3986
4246	226	194	Female	8	1.3558
4247	226	194	Female	8	1.4917

4248	3	195	Female	7	1.2486
4249	5	195	Female	7	1.2847
4250	5	195	Male	0	0.9633
4251	5	195	Male	0	1.0651
4252	5	195	Male	0	1.1582
4253	5	195	Male	0	1.1822
4254	5	195	Male	0	1.2520
4255	5	195	Male	0	1.2615
4256	5	195	Male	0	1.3074
4257	5	195	Male	0	1.3234
4258	5	195	Male	0	1.4065
4259	5	195	Male	0	1.4126
4260	5	195	Male	0	1.4273
4261	8	195	Male	0	1.3427
4262	21	195	Male	0	1.2195
4263	21	195	Male	0	1.3834
4264	22	195	Male	0	1.2466
4265	29	195	Female	6	1.1476
4266	29	195	Male	0	1.1233
4267	31	195	Male	0	1.3253
4268	31	195	Male	0	1.3472
4269	32	195	Male	0	1.3601
4270	35	195	Male	0	1.3392
4271	38	195	Female	6	1.3097
4272	38	195	Female	6	1.3825
4273	38	195	Male	0	1.0743
4274	43	195	Male	0	1.2289
4275	50	195	Female	7	1.5324
4276	50	195	Male	0	1.0234
4277	53	195	Female	7	1.1728
4278	53	195	Male	0	1.2910
4279	54	195	Male	0	1.2261
4280	54	195	Male	0	1.2440
4281	54	195	Male	0	1.2492
4282	55	195	Female	6	1.1001
4283	55	195	Male	0	1.3465
4284	55	195	Male	0	1.3583
4285	55	195	Male	0	1.3653
4286	64	195	Male	0	1.2160
4287	64	195	Male	0	1.3189
4288	66	195	Male	0	1.0170
4289	66	195	Male	0	1.2942
4290	67	195	Male	0	1.0995
4291	69	195	Female	6	1.1772

4292	70	195	Female	6	1.1984
4293	70	195	Female	6	1.3652
4294	74	195	Female	6	1.3779
4295	74	195	Male	0	0.9742
4296	74	195	Male	0	1.2526
4297	75	195	Female	7	1.2828
4298	76	195	Male	0	1.0818
4299	87	195	Male	0	1.2245
4300	90	195	Female	6	1.2118
4301	90	195	Male	0	1.1861
4302	90	195	Male	0	1.4717
4303	90	195	Male	0	1.5167
4304	98	195	Female	8	1.2087
4305	99	195	Male	0	1.0714
4306	101	195	Male	0	1.4017
4307	102	195	Male	0	1.4797
4308	105	195	Male	0	1.2154
4309	200	195	Female	6	1.0541
4310	204	195	Female	6	1.1576
4311	204	195	Female	6	1.4296
4312	204	195	Female	6	1.4416
4313	204	195	Male	0	1.1305
4314	204	195	Male	0	1.2403
4315	205	195	Female	7	1.3789
4316	205	195	Female	7	1.4516
4317	208	195	Male	0	1.4530
4318	212	195	Male	0	1.2945
4319	215	195	Female	7	1.3815
4320	215	195	Female	7	1.5268
4321	215	195	Female	7	1.5341
4322	215	195	Female	8	1.3862
4323	215	195	Male	0	1.3563
4324	219	195	Female	6	1.5521
4325	220	195	Male	0	1.3037
4326	222	195	Male	0	1.2938
4327	222	195	Male	0	1.2988
4328	223	195	Female	6	1.3646
4329	224	195	Male	0	1.3674
4330	226	195	Female	6	1.4777
4331	227	195	Female	8	1.4325
4332	228	195	Male	0	1.2013
4333	1	199	Female	8	1.3973
4334	5	199	Female	7	1.3606
4335	5	199	Male	0	1.1125

4336	5	199	Male	0	1.1989
4337	8	199	Female	6	1.2065
4338	11	199	Male	0	0.8441
4339	12	199	Female	6	1.2806
4340	12	199	Female	6	1.4048
4341	12	199	Male	0	1.3121
4342	12	199	Male	0	1.3558
4343	13	199	Male	0	1.3111
4344	13	199	Male	0	1.3287
4345	18	199	Female	7	1.2508
4346	18	199	Male	0	1.2744
4347	19	199	Female	7	1.2893
4348	19	199	Male	0	1.3776
4349	20	199	Female	6	1.1905
4350	20	199	Male	0	1.2676
4351	20	199	Male	0	1.8623
4352	21	199	Male	0	1.2523
4353	21	199	Male	0	1.3075
4354	26	199	Female	6	1.4551
4355	26	199	Male	0	1.3598
4356	26	199	Male	0	1.3992
4357	26	199	Male	0	1.4360
4358	26	199	Male	0	1.4628
4359	32	199	Male	0	1.2906
4360	32	199	Male	0	1.3269
4361	36	199	Female	6	1.2037
4362	36	199	Male	0	1.0563
4363	36	199	Male	0	1.1192
4364	36	199	Male	0	1.1866
4365	36	199	Male	0	1.2567
4366	36	199	Male	0	1.3880
4367	36	199	Male	0	1.4278
4368	50	199	Female	7	1.3453
4369	50	199	Male	0	1.2781
4370	51	199	Female	8	1.1912
4371	54	199	Female	6	1.1868
4372	54	199	Female	8	1.1589
4373	59	199	Male	0	1.0089
4374	60	199	Female	6	1.6516
4375	60	199	Female	6	1.8503
4376	60	199	Male	0	1.3052
4377	60	199	Male	0	1.3959
4378	60	199	Male	0	1.5905
4379	61	199	Female	7	1.2546

4380	61	199	Female	7	1.3994
4381	61	199	Male	0	1.2897
4382	61	199	Male	0	1.3102
4383	64	199	Female	6	1.2755
4384	64	199	Female	6	1.5098
4385	64	199	Male	0	1.2298
4386	64	199	Male	0	1.2583
4387	64	199	Male	0	1.2981
4388	64	199	Male	0	1.4305
4389	64	199	Male	0	1.4362
4390	64	199	Male	0	1.4373
4391	66	199	Female	7	1.2473
4392	66	199	Male	0	1.0129
4393	66	199	Male	0	1.0686
4394	66	199	Male	0	1.2849
4395	70	199	Male	0	1.3409
4396	71	199	Male	0	1.2201
4397	74	199	Female	6	1.3488
4398	74	199	Female	6	1.4246
4399	75	199	Female	6	1.4032
4400	75	199	Female	7	1.2476
4401	75	199	Female	7	1.2769
4402	75	199	Female	9	1.2766
4403	75	199	Male	0	1.3439
4404	75	199	Male	0	1.4350
4405	77	199	Male	0	1.2805
4406	77	199	Male	0	1.2892
4407	81	199	Female	7	1.3502
4408	81	199	Female	8	1.3234
4409	81	199	Female	8	1.3553
4410	81	199	Male	0	0.9917
4411	81	199	Male	0	1.0066
4412	81	199	Male	0	1.0422
4413	81	199	Male	0	1.0525
4414	81	199	Male	0	1.3427
4415	90	199	Female	7	1.4266
4416	91	199	Female	6	1.3271
4417	96	199	Female	7	1.2368
4418	96	199	Male	0	1.1388
4419	97	199	Female	6	1.3001
4420	97	199	Male	0	1.0930
4421	99	199	Male	0	1.2235
4422	101	199	Female	7	1.0281
4423	101	199	Female	7	1.2569

4424	101	199	Female	8	1.2732
4425	101	199	Male	0	1.2635
4426	101	199	Male	0	1.3653
4427	107	199	Female	7	1.2223
4428	107	199	Female	7	1.2517
4429	107	199	Female	7	1.3126
4430	107	199	Male	0	1.2577
4431	200	199	Male	0	1.4843
4432	204	199	Male	0	1.3725
4433	205	199	Female	6	1.0398
4434	207	199	Female	6	1.2317
4435	207	199	Female	6	1.2855
4436	207	199	Female	6	1.3072
4437	207	199	Female	6	1.3421
4438	207	199	Female	7	1.2360
4439	207	199	Female	7	1.2422
4440	207	199	Female	7	1.2843
4441	207	199	Female	7	1.4054
4442	207	199	Female	7	1.4670
4443	207	199	Female	7	1.4752
4444	207	199	Female	7	1.4873
4445	207	199	Male	0	1.4116
4446	207	199	Male	0	1.4621
4447	207	199	Male	0	1.4872
4448	210	199	Female	6	1.2703
4449	210	199	Female	6	1.3308
4450	210	199	Female	6	1.3903
4451	210	199	Female	6	1.3959
4452	210	199	Female	6	1.3998
4453	210	199	Female	6	1.4915
4454	210	199	Female	7	1.2368
4455	210	199	Female	7	1.2821
4456	210	199	Male	0	1.4344
4457	211	199	Male	0	1.3530
4458	211	199	Male	0	1.3576
4459	213	199	Male	0	1.0294
4460	213	199	Male	0	1.1533
4461	213	199	Male	0	1.1975
4462	213	199	Male	0	1.2077
4463	215	199	Female	6	1.1205
4464	215	199	Female	6	1.1421
4465	215	199	Female	8	1.4546
4466	215	199	Male	0	1.4245
4467	215	199	Male	0	1.5884

4468	217	199	Female	9	1.1091
4469	217	199	Male	0	1.1642
4470	217	199	Male	0	1.1808
4471	218	199	Female	7	1.2149
4472	218	199	Female	7	1.2673
4473	218	199	Male	0	1.2348
4474	219	199	Female	6	1.3209
4475	219	199	Female	6	1.4954
4476	219	199	Female	7	1.2445
4477	219	199	Female	7	1.4888
4478	219	199	Female	8	1.1655
4479	219	199	Female	8	1.2947
4480	219	199	Male	0	1.2108
4481	219	199	Male	0	1.2620
4482	219	199	Male	0	1.3381
4483	219	199	Male	0	1.3449
4484	220	199	Female	6	1.3759
4485	220	199	Female	6	1.5316
4486	220	199	Female	7	1.2143
4487	220	199	Male	0	1.1527
4488	220	199	Male	0	1.2383
4489	220	199	Male	0	1.2490
4490	220	199	Male	0	1.2507
4491	220	199	Male	0	1.2531
4492	220	199	Male	0	1.2625
4493	220	199	Male	0	1.4076
4494	222	199	Female	6	1.1621
4495	222	199	Female	6	1.2680
4496	222	199	Female	6	1.3182
4497	222	199	Female	7	1.2906
4498	222	199	Male	0	1.2429
4499	222	199	Male	0	1.4006
4500	224	199	Female	6	1.3060
4501	224	199	Female	6	1.3549
4502	224	199	Female	6	1.5771
4503	226	199	Female	6	1.1137
4504	226	199	Female	6	1.1431
4505	226	199	Female	6	1.3296
4506	226	199	Male	0	1.1703
4507	227	199	Female	6	1.0605
4508	227	199	Female	7	1.4220
4509	227	199	Male	0	1.1975
4510	227	199	Male	0	1.3322
4511	227	199	Male	0	1.3723

4512	1	200	Male	0	1.1498
4513	5	200	Female	6	1.4011
4514	5	200	Male	0	1.0301
4515	12	200	Female	6	1.2081
4516	12	200	Female	6	1.4199
4517	12	200	Male	0	1.4230
4518	13	200	Male	0	1.2781
4519	13	200	Male	0	1.2943
4520	19	200	Female	6	1.3986
4521	20	200	Male	0	0.9867
4522	21	200	Male	0	1.4047
4523	26	200	Female	6	1.3497
4524	26	200	Male	0	1.3224
4525	32	200	Male	0	1.2517
4526	32	200	Male	0	1.4189
4527	36	200	Male	0	1.1639
4528	36	200	Male	0	1.3757
4529	42	200	Male	0	1.2415
4530	50	200	Female	6	1.1990
4531	54	200	Female	6	1.1393
4532	54	200	Female	6	1.2038
4533	56	200	Female	8	1.2711
4534	59	200	Male	0	1.0588
4535	61	200	Female	6	1.3054
4536	61	200	Male	0	1.3174
4537	64	200	Female	6	1.4051
4538	64	200	Female	6	1.4974
4539	64	200	Male	0	1.3067
4540	64	200	Male	0	1.3357
4541	70	200	Male	0	1.2668
4542	70	200	Male	0	1.3226
4543	74	200	Female	6	1.3136
4544	74	200	Female	7	1.4286
4545	75	200	Female	7	1.2327
4546	75	200	Female	9	1.3547
4547	75	200	Male	0	1.1795
4548	75	200	Male	0	1.3307
4549	77	200	Male	0	1.0622
4550	77	200	Male	0	1.3146
4551	88	200	Male	0	1.0118
4552	88	200	Male	0	1.2692
4553	89	200	Female	7	1.2312
4554	96	200	Female	7	1.2833
4555	96	200	Male	0	1.1703

4556	97	200	Female	6	1.2888
4557	97	200	Male	0	1.1336
4558	99	200	Male	0	1.1255
4559	107	200	Male	0	1.2466
4560	200	200	Male	0	1.5556
4561	204	200	Female	6	1.3458
4562	204	200	Female	6	1.3976
4563	204	200	Female	6	1.4021
4564	204	200	Female	6	1.4316
4565	204	200	Female	6	1.4362
4566	204	200	Female	6	1.4692
4567	204	200	Male	0	1.4830
4568	207	200	Female	7	1.2775
4569	207	200	Male	0	1.5526
4570	207	200	Male	0	1.5648
4571	211	200	Male	0	1.4008
4572	211	200	Male	0	1.4662
4573	215	200	Female	6	1.3402
4574	215	200	Male	0	1.4523
4575	215	200	Male	0	1.4977
4576	219	200	Female	6	1.1500
4577	219	200	Female	9	1.3889
4578	219	200	Male	0	1.3584
4579	219	200	Male	0	1.3944
4580	220	200	Female	7	1.1481
4581	222	200	Female	7	1.4416
4582	222	200	Male	0	1.2061
4583	222	200	Male	0	1.3708
4584	224	200	Female	6	1.3544
4585	224	200	Female	6	1.4345
4586	224	200	Female	6	1.5413
4587	226	200	Female	6	1.4268
4588	226	200	Female	6	1.4417
4589	226	200	Female	6	1.4850
4590	226	200	Male	0	1.1976
4591	227	200	Male	0	1.3545
4592	227	200	Male	0	1.3832
4593	1	201	Female	7	1.2625
4594	3	201	Male	0	1.2781
4595	4	201	Female	6	1.4741
4596	8	201	Male	0	1.3612
4597	11	201	Female	6	1.0343
4598	11	201	Female	7	1.3205
4599	11	201	Male	0	1.0572

4600	11	201	Male	0	1.1011
4601	11	201	Male	0	1.2254
4602	11	201	Male	0	1.2684
4603	15	201	Female	6	1.4861
4604	20	201	Female	7	1.3205
4605	20	201	Male	0	1.2201
4606	20	201	Male	0	1.6332
4607	21	201	Female	6	1.0234
4608	21	201	Female	6	1.3114
4609	21	201	Female	6	1.3339
4610	21	201	Female	7	1.3026
4611	24	201	Female	6	1.4412
4612	24	201	Male	0	1.2114
4613	24	201	Male	0	1.3782
4614	26	201	Female	6	1.3563
4615	26	201	Male	0	1.3979
4616	26	201	Male	0	1.4089
4617	31	201	Male	0	1.3221
4618	31	201	Male	0	1.7480
4619	32	201	Male	0	1.1881
4620	38	201	Male	0	1.2962
4621	38	201	Male	0	1.3681
4622	43	201	Male	0	1.2062
4623	50	201	Female	6	1.2354
4624	51	201	Male	0	1.2211
4625	54	201	Female	6	1.2598
4626	54	201	Male	0	1.2051
4627	54	201	Male	0	1.2698
4628	55	201	Female	6	1.2688
4629	60	201	Male	0	1.2708
4630	61	201	Female	6	1.3258
4631	61	201	Female	6	1.3447
4632	61	201	Female	6	1.3543
4633	61	201	Male	0	1.2502
4634	63	201	Male	0	0.9544
4635	63	201	Male	0	0.9872
4636	63	201	Male	0	1.1138
4637	63	201	Male	0	1.4752
4638	64	201	Female	6	1.4798
4639	64	201	Male	0	1.4270
4640	66	201	Female	6	1.2321
4641	66	201	Female	6	1.2517
4642	66	201	Male	0	1.1419
4643	67	201	Male	0	1.0398

4644	69	201	Female	6	1.1297
4645	70	201	Female	6	1.2017
4646	70	201	Male	0	1.3050
4647	74	201	Male	0	1.1614
4648	75	201	Female	6	1.2964
4649	75	201	Female	6	1.2972
4650	75	201	Female	6	1.3414
4651	75	201	Female	6	1.4499
4652	75	201	Female	7	1.2558
4653	75	201	Male	0	1.2898
4654	77	201	Male	0	1.2454
4655	77	201	Male	0	1.2755
4656	77	201	Male	0	1.3065
4657	83	201	Male	0	1.2359
4658	86	201	Female	6	1.2567
4659	86	201	Male	0	1.1297
4660	86	201	Male	0	1.3019
4661	87	201	Female	6	1.2117
4662	87	201	Male	0	1.4144
4663	88	201	Female	8	1.3001
4664	88	201	Male	0	1.1159
4665	88	201	Male	0	1.1949
4666	91	201	Female	6	1.4068
4667	96	201	Female	7	1.3146
4668	97	201	Female	6	1.2219
4669	98	201	Female	6	1.1526
4670	98	201	Female	6	1.2310
4671	98	201	Female	6	1.2891
4672	98	201	Female	7	1.2574
4673	99	201	Female	8	1.0018
4674	99	201	Female	10	1.1951
4675	99	201	Male	0	1.0823
4676	99	201	Male	0	1.2542
4677	105	201	Female	7	1.2402
4678	105	201	Male	0	1.2193
4679	107	201	Female	7	1.2239
4680	204	201	Female	7	1.3709
4681	206	201	Female	6	1.2705
4682	206	201	Female	6	1.2711
4683	206	201	Male	0	1.4429
4684	207	201	Female	6	1.3572
4685	207	201	Female	7	1.4491
4686	207	201	Male	0	1.3626
4687	207	201	Male	0	1.5494

4688	207	201	Male	0	1.5671
4689	208	201	Female	6	1.3113
4690	208	201	Female	7	1.4045
4691	208	201	Male	0	1.3865
4692	208	201	Male	0	1.4384
4693	209	201	Male	0	1.3871
4694	210	201	Female	6	1.2710
4695	210	201	Female	6	1.4449
4696	213	201	Male	0	1.0385
4697	215	201	Female	6	1.4984
4698	215	201	Female	8	1.3977
4699	219	201	Female	6	1.2840
4700	219	201	Male	0	1.1294
4701	219	201	Male	0	1.1396
4702	219	201	Male	0	1.3993
4703	222	201	Female	6	1.4090
4704	223	201	Female	6	1.2263
4705	224	201	Female	6	1.0412
4706	224	201	Female	6	1.1278
4707	224	201	Female	6	1.2227
4708	224	201	Female	6	1.4486
4709	224	201	Female	7	1.2662
4710	224	201	Female	7	1.2999
4711	224	201	Female	7	1.5047
4712	224	201	Male	0	1.1669
4713	224	201	Male	0	1.2697
4714	227	201	Male	0	1.2495
4715	227	201	Male	0	1.3896
4716	4	202	Male	0	1.3712
4717	5	202	Female	7	1.1595
4718	5	202	Male	0	1.1118
4719	11	202	Female	6	1.2816
4720	12	202	Male	0	1.3175
4721	20	202	Male	0	1.9473
4722	31	202	Male	0	1.1178
4723	53	202	Male	0	1.2071
4724	55	202	Male	0	1.1834
4725	61	202	Male	0	1.3984
4726	61	202	Male	0	1.4315
4727	64	202	Male	0	1.2933
4728	67	202	Female	6	1.2325
4729	70	202	Male	0	1.2488
4730	71	202	Male	0	1.2671
4731	77	202	Male	0	1.1246

4732	77	202	Male	0	1.2226
4733	90	202	Female	7	1.2258
4734	90	202	Female	7	1.2814
4735	90	202	Male	0	1.5061
4736	96	202	Male	0	1.0643
4737	96	202	Male	0	1.1318
4738	97	202	Female	6	1.3276
4739	99	202	Female	7	1.1639
4740	99	202	Female	8	1.2432
4741	99	202	Male	0	1.1472
4742	205	202	Female	6	1.1755
4743	207	202	Female	7	1.2299
4744	207	202	Male	0	1.4005
4745	213	202	Female	6	1.4244
4746	213	202	Male	0	1.0241
4747	215	202	Female	6	1.1344
4748	215	202	Female	6	1.1612
4749	215	202	Male	0	1.3775
4750	215	202	Male	0	1.4475
4751	215	202	Male	0	1.4741
4752	219	202	Female	7	1.3463
4753	219	202	Male	0	1.1693
4754	219	202	Male	0	1.2912
4755	220	202	Female	6	1.2687
4756	220	202	Male	0	1.3297
4757	224	202	Female	6	1.3701
4758	224	202	Male	0	1.3705
4759	227	202	Male	0	1.2257
4760	1	207	Female	7	1.3674
4761	1	207	Female	8	1.3680
4762	4	207	Female	6	1.5217
4763	4	207	Female	7	1.0934
4764	4	207	Male	0	1.2116
4765	4	207	Male	0	1.2711
4766	4	207	Male	0	1.3117
4767	5	207	Male	0	0.9028
4768	5	207	Male	0	1.2515
4769	11	207	Female	6	1.2536
4770	12	207	Male	0	1.3735
4771	13	207	Female	6	1.2886
4772	13	207	Female	7	1.3273
4773	14	207	Female	6	1.3111
4774	14	207	Female	7	1.3042
4775	15	207	Female	9	1.3309

4776	15	207	Male	0	1.3678
4777	17	207	Female	9	1.3613
4778	20	207	Female	9	1.3192
4779	20	207	Male	0	1.1066
4780	25	207	Female	6	1.2005
4781	25	207	Female	6	1.2073
4782	25	207	Female	7	1.1086
4783	25	207	Female	8	1.0081
4784	25	207	Male	0	1.3217
4785	26	207	Female	6	1.3834
4786	26	207	Female	6	1.4192
4787	26	207	Female	8	1.3514
4788	26	207	Male	0	1.4649
4789	26	207	Male	0	1.5132
4790	26	207	Male	0	1.5252
4791	27	207	Female	7	1.1963
4792	27	207	Male	0	1.3121
4793	34	207	Male	0	1.2038
4794	34	207	Male	0	1.2713
4795	34	207	Male	0	1.3266
4796	34	207	Male	0	1.4184
4797	34	207	Male	0	1.4268
4798	35	207	Male	0	1.0432
4799	38	207	Male	0	1.3954
4800	50	207	Female	6	1.1891
4801	51	207	Female	6	1.0671
4802	52	207	Female	8	1.1780
4803	52	207	Male	0	1.2061
4804	55	207	Male	0	1.1856
4805	60	207	Female	6	1.7815
4806	60	207	Female	6	1.8198
4807	63	207	Female	6	1.2782
4808	63	207	Female	6	1.3144
4809	63	207	Male	0	1.2806
4810	63	207	Male	0	1.3417
4811	64	207	Male	0	1.3018
4812	66	207	Male	0	1.0962
4813	67	207	Female	6	1.1512
4814	67	207	Female	6	1.3944
4815	71	207	Female	6	1.1649
4816	71	207	Female	6	1.1746
4817	74	207	Male	0	1.4476
4818	75	207	Female	6	1.2607
4819	76	207	Female	6	1.2285

4820	78	207	Female	6	1.3803
4821	78	207	Female	7	1.3937
4822	86	207	Female	7	1.2554
4823	87	207	Female	8	1.3599
4824	87	207	Female	8	1.3704
4825	87	207	Male	0	1.4041
4826	88	207	Female	7	1.1386
4827	88	207	Female	8	1.2915
4828	89	207	Female	7	1.1133
4829	96	207	Female	7	1.2331
4830	97	207	Female	7	1.3232
4831	97	207	Male	0	1.1196
4832	98	207	Female	7	1.1599
4833	99	207	Female	8	1.1616
4834	101	207	Female	6	1.3379
4835	101	207	Female	7	1.2245
4836	101	207	Male	0	1.4705
4837	102	207	Male	0	1.3989
4838	102	207	Male	0	1.4603
4839	102	207	Male	0	1.4883
4840	102	207	Male	0	1.5068
4841	104	207	Female	7	1.2044
4842	105	207	Female	7	1.1640
4843	106	207	Female	7	1.2368
4844	106	207	Female	7	1.3099
4845	106	207	Female	7	1.4094
4846	106	207	Female	8	1.3114
4847	106	207	Male	0	1.4554
4848	107	207	Female	8	1.2042
4849	107	207	Female	8	1.2042
4850	210	207	Male	0	1.4632
4851	215	207	Female	6	1.3115
4852	215	207	Female	8	1.5181
4853	215	207	Male	0	1.4773
4854	217	207	Female	7	1.0523
4855	217	207	Male	0	1.0961
4856	218	207	Female	7	1.3216
4857	218	207	Male	0	1.1999
4858	219	207	Male	0	1.1253
4859	219	207	Male	0	1.2500
4860	219	207	Male	0	1.3051
4861	223	207	Female	7	1.1337
4862	223	207	Female	8	1.1268
4863	227	207	Female	6	1.0971

4864	227	207	Female	7	1.1801
4865	227	207	Female	7	1.3997
4866	227	207	Female	8	1.1995
4867	227	207	Female	8	1.4988
4868	227	207	Male	0	1.1375
4869	227	207	Male	0	1.2047
4870	1	208	Female	8	1.5206
4871	1	208	Male	0	1.3897
4872	5	208	Male	0	0.9020
4873	26	208	Male	0	1.3091
4874	26	208	Male	0	1.4481
4875	36	208	Female	6	1.2104
4876	36	208	Male	0	1.2558
4877	53	208	Female	7	1.1312
4878	54	208	Female	6	1.2079
4879	54	208	Male	0	1.1910
4880	55	208	Female	6	1.0424
4881	55	208	Female	6	1.3248
4882	55	208	Female	8	1.2517
4883	55	208	Male	0	1.2835
4884	56	208	Female	7	1.2423
4885	70	208	Female	6	1.2232
4886	70	208	Female	6	1.3085
4887	70	208	Male	0	1.3160
4888	73	208	Female	6	1.2152
4889	73	208	Female	6	1.2746
4890	73	208	Male	0	1.2257
4891	73	208	Male	0	1.2731
4892	75	208	Male	0	1.2735
4893	76	208	Male	0	0.9831
4894	76	208	Male	0	1.2467
4895	77	208	Female	6	1.0452
4896	77	208	Female	6	1.1271
4897	77	208	Male	0	0.9754
4898	78	208	Male	0	1.3496
4899	90	208	Male	0	1.3338
4900	90	208	Male	0	1.3580
4901	90	208	Male	0	1.4224
4902	90	208	Male	0	1.4919
4903	97	208	Female	6	1.1447
4904	98	208	Male	0	1.1830
4905	99	208	Male	0	1.3318
4906	101	208	Female	7	1.0622
4907	204	208	Male	0	1.0313

4908	204	208	Male	0	1.4699
4909	204	208	Male	0	1.5969
4910	208	208	Female	6	1.3737
4911	219	208	Female	7	1.5185
4912	222	208	Male	0	1.1284
4913	222	208	Male	0	1.1626
4914	227	208	Female	7	1.3136
4915	1	212	Female	7	1.3439
4916	3	212	Female	6	1.1071
4917	3	212	Female	6	1.2138
4918	4	212	Female	7	1.4162
4919	5	212	Female	6	1.3221
4920	5	212	Female	7	1.3381
4921	5	212	Male	0	1.0373
4922	5	212	Male	0	1.1399
4923	11	212	Male	0	1.3326
4924	27	212	Male	0	1.2313
4925	29	212	Female	6	1.4097
4926	31	212	Male	0	1.2811
4927	32	212	Male	0	1.1341
4928	35	212	Female	6	1.2673
4929	51	212	Female	6	1.0342
4930	53	212	Female	7	1.1647
4931	53	212	Male	0	1.0883
4932	60	212	Female	7	2.1680
4933	61	212	Male	0	1.3032
4934	61	212	Male	0	1.3231
4935	62	212	Male	0	0.9802
4936	62	212	Male	0	1.4752
4937	64	212	Female	6	1.3843
4938	64	212	Male	0	1.3318
4939	73	212	Female	7	1.2982
4940	73	212	Male	0	1.1390
4941	73	212	Male	0	1.4206
4942	75	212	Male	0	1.1110
4943	75	212	Male	0	1.2200
4944	76	212	Male	0	0.9982
4945	76	212	Male	0	1.0788
4946	79	212	Female	6	1.1728
4947	79	212	Male	0	1.2319
4948	81	212	Male	0	1.1345
4949	83	212	Male	0	1.2393
4950	83	212	Male	0	1.2403
4951	86	212	Male	0	0.9431

4952	86	212	Male	0	1.2088
4953	86	212	Male	0	1.3296
4954	90	212	Female	7	1.3701
4955	96	212	Male	0	0.9076
4956	96	212	Male	0	1.2123
4957	97	212	Female	6	1.1616
4958	97	212	Female	6	1.3220
4959	105	212	Male	0	1.2191
4960	105	212	Male	0	1.2306
4961	106	212	Female	7	1.2521
4962	106	212	Male	0	1.3066
4963	206	212	Female	6	1.0657
4964	207	212	Male	0	1.4786
4965	207	212	Male	0	1.5664
4966	214	212	Male	0	1.1188
4967	214	212	Male	0	1.2367
4968	216	212	Female	6	1.4167
4969	216	212	Female	7	1.5441
4970	219	212	Male	0	1.1332
4971	219	212	Male	0	1.2407
4972	222	212	Male	0	1.1994
4973	222	212	Male	0	1.3826
4974	223	212	Female	7	1.2265
4975	224	212	Male	0	1.3768
4976	227	212	Female	6	1.1971
4977	227	212	Male	0	1.1897
4978	31	213	Female	6	1.3544
4979	36	213	Male	0	1.3163
4980	42	213	Female	7	1.2769
4981	43	213	Male	0	1.2709
4982	61	213	Male	0	0.9050
4983	64	213	Female	6	1.3338
4984	65	213	Male	0	1.2619
4985	67	213	Female	6	1.2993
4986	69	213	Male	0	1.3111
4987	75	213	Female	6	1.2117
4988	78	213	Female	6	1.3227
4989	78	213	Female	7	1.3957
4990	78	213	Male	0	1.2966
4991	78	213	Male	0	1.4091
4992	90	213	Female	6	1.1889
4993	90	213	Male	0	1.1878
4994	97	213	Female	6	1.1437
4995	97	213	Male	0	1.2323

4996	99	213	Female	7	1.2615
4997	5	214	Male	0	0.9524
4998	59	214	Male	0	1.2719
4999	61	214	Male	0	1.0553
5000	75	214	Male	0	1.1921
5001	96	214	Male	0	0.9492
5002	99	214	Female	7	1.2615
5003	99	214	Male	0	1.1730
5004	12	215	Male	0	1.2722
5005	16	215	Female	6	1.2618
5006	16	215	Female	9	1.1904
5007	24	215	Female	7	1.1826
5008	35	215	Female	5	1.1211
5009	53	215	Male	0	1.1276
5010	53	215	Male	0	1.1684
5011	54	215	Male	0	1.2625
5012	55	215	Female	7	1.3643
5013	61	215	Male	0	1.4041
5014	66	215	Male	0	1.0696
5015	75	215	Male	0	1.3097
5016	76	215	Female	6	1.3578
5017	77	215	Male	0	1.2682
5018	78	215	Male	0	1.2458
5019	91	215	Female	6	1.4797
5020	107	215	Male	0	1.2660
5021	29	219	Female	6	1.3475
5022	63	219	Female	6	1.2311
5023	96	219	Female	6	1.1939
5024	224	219	Female	7	1.1209
5025	35	220	Male	0	1.2528
5026	53	220	Male	0	1.1745
5027	53	220	Male	0	1.2344
5028	53	220	Male	0	1.2941
5029	65	220	Male	0	1.2352
5030	70	220	Female	6	1.3413
5031	73	220	Male	0	1.1576
5032	76	220	Male	0	1.0321
5033	78	220	Male	0	1.3083
5034	96	220	Male	0	1.1968
5035	98	220	Male	0	1.1845
5036	215	220	Female	7	1.4976
5037	215	220	Male	0	1.3450
5038	222	220	Male	0	1.3103
5039	223	220	Male	0	1.3307

5040	24	222	Male	0	1.2903
5041	60	222	Male	0	1.9301
5042	97	222	Male	0	1.2112
5043	98	222	Male	0	1.0340
5044	18	226	Female	6	1.3021
5045	61	226	Female	6	1.3163
5046	61	226	Female	6	1.3537
5047	69	226	Male	0	1.2852
5048	70	226	Male	0	1.3101
5049	76	226	Female	7	1.3024
5050	107	226	Female	6	1.0962
5051	107	226	Male	0	1.2603
5052	107	226	Male	0	1.2850
5053	107	226	Male	0	1.3113
5054	70	233	Male	0	1.2900
5055	77	233	Female	6	1.1621
5056	81	233	Male	0	1.2837
5057	106	233	Female	6	1.1182
5058	107	233	Male	0	1.2808
5059	107	233	Male	0	1.2976
