# Supplementary material for: Effect of Temperature and Genetic Inheritance on the Number of Mycangium Pits in Female Platypus quercivorus (Coleoptera: Curculionidae: Platypodinae)
Source: Insects. 2026 May 22;17(6):536. doi: 10.3390/insects17060536 (PMC13300474; doi:10.3390/insects17060536)

Figure S1. Temporal trajectories of the within-brood mean number of mycangial pits in *Platypus quercivorus*, calculated from female individuals in Table S1 collected from Japanese Oak Wilt–infested *Quercus serrata* host trees at the University of Tokyo Tanashi Forest in 2023, illustrating the progression of cumulative mean estimates over time and enabling comparison and classification of broods at a common time point. The cumulative mean was recalculated at each sampling event as the mean number of mycangial pits across all individuals collected up to that point. Each broken line represents the temporal changes in the mean number of pits of a single brood (111 broods in total) as additional individuals were incorporated over the sampling period (Julian dates 160–233), allowing comparison among broods. Individuals were collected on weekdays, excluding weekends and holidays. The red vertical line (Day 180) indicates the time point selected based on visual inspection of the trajectories, where cumulative mean values showed minimal change with additional samples, and used for brood classification. Broods were classified into three groups based on cumulative mean values at Day 180: small (≤ 6.57), medium (> 6.57 and < 7.90), and large (≥ 7.90). The thick black horizontal lines indicate the boundaries of these groups. To evaluate the genetic effects on the number of mycangium pits, we paired a male and a female from different broods within the same size category. These pairs were inoculated into logs starting July 3, 2023. Broods categorized into medium were not used for the experiment.

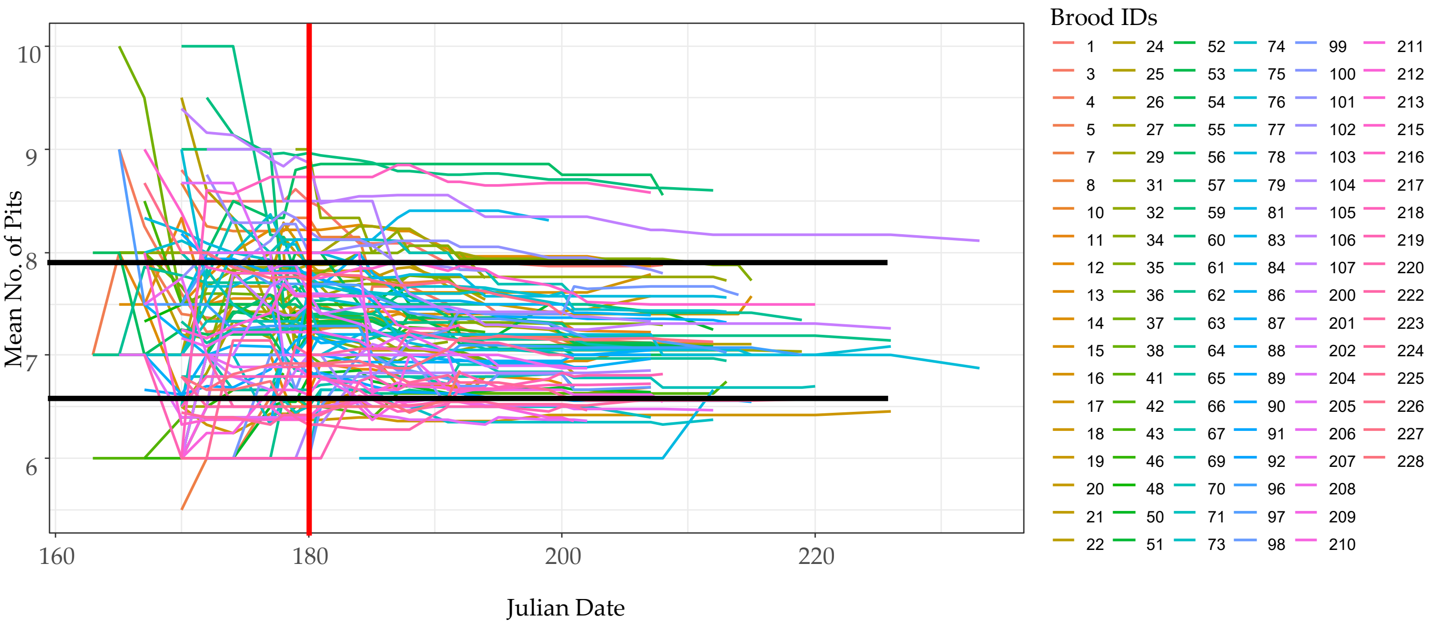

Supplement: Supplementary file 1 [file insects-17-00536-s001.zip › Figure_S1.pdf]
